# Use of anthracyclines and trastuzumab for breast cancer in women with and without a history of cardiovascular disease in Sweden: a national cross-sectional study

**DOI:** 10.1186/s40959-025-00356-z

**Published:** 2025-06-20

**Authors:** Helena Carreira, Helen Strongman, Maria Feychting, Laila Hubbert, Elham Hedayati, Patrick Bidulka, Anthony Matthews, Krishnan Bhaskaran

**Affiliations:** 1 Electronic Health Records Research Group, Department of Non-Communicable Diseases Epidemiology, Faculty of Epidemiology and Population Health, London School of Hygiene and Tropical Medicine, London, WC1E 7HT UK; 2 Unit of Epidemiology, Institute of Environmental Medicine, Karolinska Institutet, Stockholm, 171 77 Sweden; 3 Department of Cardiology and Department of Health, Medicine and Caring Sciences, Linkoping University, Norrkoping, Sweden

## Abstract

**Background:**

Cardiovascular toxicity concerns have limited the use of anthracyclines and trastuzumab among breast cancer patients with cardiovascular disease (CVD) but evidence on real-world prescribing patterns is scarce. We aimed to describe the use of these drugs in women with and without CVD when diagnosed with non-metastatic breast cancer in Sweden.

**Methods:**

Using Swedish national registers (2010–15), we identified breast cancer treatment and prior CVD from hospital and prescription data. We calculated prevalence of anthracycline and trastuzumab use in women with and without prior CVD, and estimated prevalence ratios (PR) comparing these groups, adjusted for age, stage, and other patient and tumour-related factors.

**Results:**

Among 32,590 women with breast cancer, 10,702 (33%) had prior CVD. Anthracycline use was lower in those with vs without prior CVD (2,169/10,702 [20.3%] vs 8,654/21,888 [39.5%], crude PR 0.51, 0.49–0.53); the PR attenuated after adjustment for age and other factors (adj-PR 0.90, 0.87–0.93). There was substantial variation by type of CVD: patients with heart failure were much less likely to receive anthracyclines (adj-PR 0.46, 0.35–0.57) while prior venous thromboembolism (VTE) had no impact (adj-PR 0.98, 0.88–1.09). Among HER2 + patients, trastuzumab use showed similar patterns, with prevalence of 630/1,100 [57.3%] vs 2,279/2,866 [79.5%] for any vs no prior CVD (crude PR = 0.72, 0.68–0.76, adjusted PR = 0.95, 0.90–0.99); adjusted PRs for specific outcomes ranged from 0.77 (0.61–0.93) for heart failure, to 1.04 (0.92–1.15) for VTE.

**Conclusion:**

While prior CVD was associated with lower use of potentially cardiotoxic breast cancer therapies, substantial numbers of patients with CVD still received these treatments, with marked variation by type of CVD. These real-world data suggest variable cardiovascular toxicity risk stratification before anticancer therapy and highlight the need for evidence-based guidance on negotiating the risk–benefit balance in these patients.

**Supplementary Information:**

The online version contains supplementary material available at 10.1186/s40959-025-00356-z.

## Introduction

Breast cancer is the most common cancer diagnosed in women worldwide, with 2.2 million newly diagnosed cases in 2022 alone [1]. Prognosis from breast cancer improved remarkably in high-income settings over recent decades. In Sweden, 5-year age-standardised relative survival from breast cancer increased from 82% in 1990 to 90% in 2015 [2]. Improved breast cancer outcomes are largely the results of earlier detection and better treatments. However, some highly effective anti-cancer drugs such as anthracyclines and trastuzumab carry important cardiovascular toxicity [3, 4], and breast cancer management guidelines have recommended against the use of anthracycline-based regimens in patients with low cardiac function and/or high risk of adverse cardiovascular outcomes [5, 6].

The extent to which anthracyclines and trastuzumab have been used in breast cancer patients with comorbid cardiovascular disease (CVD) in routine clinical practice is currently unclear. Understanding real-world prescribing patterns is crucial given the evolving evidence base and changing risk–benefit considerations. While treatment guidance historically has suggested complete exclusion of high-risk cardiovascular patients from receiving these therapies, the focus more recently has shifted towards tailoring both oncological and cardiovacular therapies to the patient, to ensure maximum anti-cancer benefit whilst minimising short- and long-term cardiac toxicities [7]. However, without data on prescribing patterns, it is not possible to assess how clinical practice aligns with the evolving recommendations, nor evaluate whether certain patient groups might be unnecessarily denied effective treatment options. Conversely, there may be subgroups of patients receiving these agents despite having risk factors that could warrant alternative approaches. Real-world evidence on drug utilisation among higher-risk populations are also necessary, as randomized trials have typically excluded patients with pre-existing cardiac comorbidities.

The national collection of clinical and treatment data for breast cancer patients in Sweden provides an opportunity to study these questions at a population level. We aimed to investigate the associations between the history of CVD and the use of anthracyclines and trastuzumab in a national cohort of women diagnosed with non-metastatic breast cancer between 2010 and 2015 in Sweden, and to describe the characteristics of the women with CVD who received such treatments.

## Methods

### Study design and data sources

This was a population-based cross-sectional study that used individually linked data from five registers in Sweden: the National Quality Register for Breast Cancer [8]; the Prescribed Drug Register [9]; the National Patient Register [10]; the National Cause of Death Register [11]; the Total Population Register [12]; and the Longitudinal Integrated Database for Health Insurance and Labor market studies (LISA) [13].

The National Quality Register for Breast Cancer includes data on diagnoses, treatment and outcomes of all patients diagnosed with breast tumours in Sweden since 2008 [8]. Validation against the National Cancer Register showed 99.9% completeness [8]. Less than 1% of the records have missing data on stage at diagnosis, hormone and human epidermal growth factor receptor 2 (HER2) status, and adjuvant treatments [8]. Data in the register have good agreement with data in the clinical records (> 80%) [8].

The National Quality Register for Breast Cancer data were linked to the other Registers to obtain data on socio-demographics, comorbidities and out of hospital treatments. All linkages were based on the Personal Identity Number (PIN), a unique identifier for individuals in Sweden that is used in all databases [14]. Supplementary Table 1 provides detailed information on the data abstracted from each database.

### Study population

The study population included women with breast cancer identified from the National Quality Register for Breast Cancer. All women aged 18 or above, diagnosed with unilateral non-metastatic breast cancer between 01–01–2010 and 31–12–2015 were eligible. This period was chosen because a validation study showed high data quality after 2010 [8] and all other data sources covered the period up to 2015. Exclusion criteria were synchronous bilateral breast cancer, tumours with metastatic, in situ or uncertain behaviour, and another cancer diagnosis at any point in time before their breast cancer (to exclude women with CVD that may have been caused by previous cancer treatment).

### Study variables

CVDs were identified from the National Patient Register [10], using International Classification of Diseases 10th revision (ICD-10) codes for diseases of the circulatory system (I chapter). The National Patient Register includes data on hospitalisations (since 1998) and outpatient specialist care appointments. We searched all primary and secondary diagnoses fields for relevant diagnoses up to the date of breast cancer diagnosis. Specific CVDs of interest included coronary heart disease (CAD), heart failure, primary hypertension, stroke and venous thromboembolism (VTE). The National Patient Register was also used to identify other chronic conditions, including chronic kidney disease (CKD), chronic obstructive pulmonary disease (COPD), and diabetes. We further supplemented the definition of diabetes with data on antidiabetic prescriptions from the Swedish Prescribed Drug Register [Anatomical Therapeutic Chemical (ATC) codes A10] to minimise misclassification, as most cases of diabetes are managed in primary care. ICD-10 code lists for all conditions are shown in Supplementary Table 2.

The tumour characteristics used in this study [i.e., oestrogen receptor (ER) status, progesterone receptor (PR) status, human epidermal growth factor receptor 2 (HER2) status, grade, and TNM stage at diagnosis] were those recorded in the National Quality Register for Breast Cancer, ascertained through histological analysis and pathological verification. The demographic (i.e., age, region, civil status) and socio-economic characteristics (i.e., education, disposable income) of all participants were obtained by record linkage to the Total Population Register and LISA, and refer to the year before the breast cancer diagnosis.

Data on breast cancer treatments in the year after breast cancer diagnosis were obtained from the National Quality Register for Breast Cancer. Information on use of endocrine therapy in the year after diagnosis was further supplemented by prescriptions in the National Prescribed Drug Register [9]. We ascertained the treatment modalities received (i.e. radiotherapy, chemotherapy, immunotherapy, hormone therapy), and classified the type of chemotherapy drugs used (i.e. anthracyclines, docetaxel, paclitaxel, and others); the type of immunotherapies used (i.e. trastuzumab vs. pertuzumab); and the type of hormonal therapies used (i.e., tamoxifen, aromatase inhibitors, gonadotropin-releasing hormone (GNRH) agonists, and other anti-oestrogens). Treatments were further classified as neo-adjuvant when the date of first administration preceded the date of surgery, and adjuvant when administered after surgery.

### Statistical analysis

Numbers and proportions were used to describe the characteristics of breast cancer patients with and without CVD at diagnosis, their tumours and treatments received in the first year after diagnosis. Results were also presented as mean and standard deviation (SD) or median and interquartile range (IQR), as deemed appropriate after visual inspection of histograms.

We calculated the crude prevalence and prevalence ratios of anthracycline and trastuzumab use among women with and without CVD, overall [composite outcome of coronary heart disease (CAD), heart failure, primary hypertension and stroke], overall adding VTE, overall excluding hypertension, and by type of CVD (i.e., CAD, heart failure, hypertension, stroke, and VTE). In analyses by type of CVD, the comparison group include all patients without that specific CVD. We further calculated prevalence ratios stratified by setting (neo-adjuvant vs. adjuvant) and molecular subtype of breast cancer (luminal, HER2+ and triple negative) to understand whether associations were similar in these subgroups. Analyses of trastuzumab were restricted to patients with HER2+ tumours.

Adjusted prevalence ratios were estimated comparing anthracycline and trastuzumab use (separately) in those with versus without prior CVD. Multivariable logistic regression models were used and adjusted for age only (5-year age groups, minimally adjusted model), and then further adjusted for region, socio-economic status (education, civil status, and disposable income), comorbidities (CKD, COPD and diabetes), and tumour-related variables (stage at diagnosis, HER2 receptor status for anthracyclines, tumour grade and diagnostic subtype) (fully adjusted model). We then calculated predicted probabilities of medication (anthracycline, trastuzumab) use for both CVD groups standardised to the observed distribution of other characteristics in our study population. Prevalence ratios were calculated as the ratio of these predicted probabilities, with 95% confidence intervals estimated using the delta method [15].

The proportion of patients with missing data was very low for most variables (<1% for socio-demographics; <3% for treatment), except for tumour grade that was missing for 15% of patients. Adjusted models are based on a complete case analysis.

To assess variations in treatment over time, breast cancer patients were grouped into those with or without a history of CVD and studied regarding treatment with chemotherapy, anthracyclines, trastuzumab or both, for each calendar year between 2010 and 2015. We also plotted the overall proportion of patients that received radiotherapy, endocrine therapy, and immunotherapy, per calendar year, to assess general trends in treatment use in the data.

### Ethics

The protocol of this study had favourable ethical approval from the Regional Ethical Review Board in Stockholm (2011/634–31/4, 2019–05224) and the Research Ethics Committee at the London School of Hygiene & Tropical Medicine (ref. 25,076).

## Results

### Characteristics of the study cohort

A total of 32,590 women with unilateral non-metastatic breast cancer diagnosed between 2010 and 2015 were included in the study (flowchart provided in Supplementary Fig. 1). Of these, 10,702 (32.8%) had a history of CVD (Table 1). Breast cancer patients with comorbid CVD tended to be older and diagnosed at more advanced stage compared to those without CVD. CKD, COPD and diabetes were also more common in those with CVD. Breast cancer hormone and HER2 receptor status were around 85% or more complete, but slightly less likely to be known/recorded for women with history of CVD. In total, 3,966 (13.6%) had tumours that overexpressed HER2 + and 1,100 (27.7%) of these had prior CVD.

**Table 1:** Characteristics of the study population (*N* = 32,590) by history of cardiovascular disease at breast cancer diagnosis

	No CVD history	CVD history^a^
Number of women	21,888 (67.2%)	10,702 (32.8%)
Demographics		
Age at diagnosis (years)		
Mean (SD)	59.5 (13.1)	71.3 (12.4)
Median (IQR)	60.0 (49.0, 68.0)	71.0 (64.0, 81.0)
Age group at diagnosis (years), *n* (%)		
18 to 39	1,302 (5.9)	101 (0.9)
40 to 59	9,379 (42.8)	1,624 (15.2)
60 to 79	9,685 (44.2)	5,944 (55.5)
80 plus	1,522 (7.0)	3,033 (28.3)
Year of breast cancer diagnosis, *n* (%)		
2010 to 2011	6,252 (28.6)	2,659 (24.8)
2012 to 2013	7,086 (32.4)	3,533 (33.0)
2014 to 2015	8,550 (39.1)	4,510 (42.1)
Region of Sweden, *n* (%)		
North	1,639 (7.5)	850 (7.9)
South	3,980 (18.2)	2,031 (19.0)
Southeast	2,116 (9.7)	1,077 (10.1)
Stockholm/Gotland	4,875 (22.3)	2,266 (21.2)
Uppsala/Örebro	4,653 (21.3)	2,273 (21.2)
West	4,625 (21.1)	2,205 (20.6)
Civil status, *n* (%)		
Unmarried	4,257 (19.4)	1,046 (9.8)
Married	11,568 (52.9)	4,828 (45.1)
Divorced/separated	3,924 (17.9)	1,995 (18.6)
Widow	2,133 (9.6)	2,833 (26.5)
Unknown	6 (0.0)	0 (0.0)
Level of education, *n* (%)		
Compulsory education or less	4,220 (19.3)	3,678 (34.4)
Upper secondary	9,145 (41.8)	4,249 (39.7)
College/University/Research	8,359 (38.2)	2,655 (24.8)
Unknown	164 (0.7)	120 (1.1)
Disposable income SEK, *n* (%)		
1 to 1,000	1,945 (8.9)	1,421 (13.3)
1,001 to 2,000	8,651 (39.5)	6,333 (59.2)
2,001 to 3,000	7,027 (32.1)	1,875 (17.5)
> 3,000	4,048 (18.5)	1,048 (9.8)
Unknown	217 (1.0)	25 (0.2)
Tumour characteristics, *n* (%)		
TNM stage		
Stage 1	12,456 (56.9)	5,509 (51.5)
Stage 2	8,565 (39.1)	4,697 (49.3)
Stage 3	867 (4.0)	496 (4.6)
Histological grade		
Grade 1	3,593 (16.4)	1,648 (15.4)
Grade 2	9,542 (43.6)	4,485 (41.9)
Grade 3	6,099 (27.9)	2,659 (24.8)
Unknown/unrecorded	2,654 (12.1)	1,910 (17.8)
HER2 status		
Negative	17,037 (77.8)	8,115 (75.8)
Positive	2,866 (13.1)	1,100 (10.3)
Unknown/unrecorded	1,985 (9.1)	1,487 (13.9)
ER status		
Negative	17,604 (80.4)	8,414 (78.6)
Positive	3,017 (13.8)	1,306 (12.2)
Unknown/unrecorded	1,267 (5.8)	982 (9.2)
PR status		
Negative	14,951 (68.3)	7,071 (66.1)
Positive	5,644 (25.8)	2,630 (24.6)
Unknown/unrecorded	1,293 (5.9)	1,001 (9.4)
Molecular subtype		
Luminal	15,168 (69.3)	7,294 (68.2)
HER2 +	2,886 (13.1)	1,100 (10.3)
Triple negative	1,869 (8.5)	821 (7.7)
Unknown	1,985 (9.1)	1,487 (13.9)
Menopausal status		
Pre-menopausal	5,891 (26.9)	698 (6.5)
Post-menopausal	14,140 (64.6)	9,436 (88.2)
Unknown	1857 (8.5)	568 (5.3)
Comorbidities at breast cancer diagnosis, *n* (%)		
Chronic kidney disease	22 (0.1)	208 (1.9)
COPD	285 (1.3)	655 (6.1)
Diabetes	840 (3.8)	1,660 (15.5)
Breast cancer treatment in the year after diagnosis, *n* (%)		
Surgery		
Primary operation	19,731 (90.1)	9,014 (84.2)
Pre-op oncological or conservative treatment	1,913 (8.7)	1,174 (11.0)
No surgery	242 (1.1)	511 (4.8)
Unknown	2 (0.0)	3 (0.0)
Chemotherapy		
Anthracyclines	8,654 (39.5)	2,169 (20.3)
Docetaxel	2,154 (9.8)	538 (5.0)
Paclitaxel	768 (3.5)	285 (2.7)
Other chemotherapy	473 (2.2)	163 (1.5)
Antibody therapy		
Trastuzumab	2,474 (11.3)	675 (6.3)
Pertuzumab	5 (0.0)	2 (0.0)
Endocrine therapy		
Outpatient tamoxifen	9,018 (41.2)	2,935 (27.4)
Outpatient GNRH	596 (2.7)	91 (0.9)
Outpatient AI	8,627 (39.4)	6,057 (56.6)
Radiotherapy		
Not recorded	6,761 (30.9)	5,170 (48.3)
Right breast	7,465 (34.1)	2,704 (25.3)
Left breast	7,662 (35.0)	2,828 (26.4)

*AI* aromatase inhibitors, *COPD* chronic obstructive pulmonary disease, *ER* oestrogen receptor, *GNRH* gonadotropin-releasing hormone, *HER2* human epidermal growth factor receptor 2, *IQR* interquartile range, *PR* progesterone receptor, *SEK* Swedish crown, *SD* Standard deviation, *TNM* UICC TNM Classification of Malignant Tumours

^a^CVD history defined as having one or more of the following conditions at breast cancer diagnosis: coronary heart disease (CAD), heart failure, primary hypertension and stroke. Venous thromboembolism was not considered in the composite outcome of CVD, but separate analyses are still presented

### Characteristics of patients with and without prior CVD that received and did not receive anthracyclines or trastuzumab

Table 2 shows the characteristics of breast cancer patients that received or did not receive anthracyclines stratified by history of CVD. Of the 10,702 women with a history of CVD at diagnosis, 2,169 (20.3%) received anthracyclines. Hypertension (48.8%) and CAD (9.1%) were the most common CVDs. There was little difference in the characteristics of the patients with and without CVD that used anthracyclines. In both groups, women who received anthracyclines were younger and of higher socio-economic status, had more advanced stage at diagnosis, higher grade tumours, and more often HER2, oestrogen, or progesterone receptor-positive tumours. Patterns by individual types of CVD were similar (Supplementary tables 3 to 8). Among those with CVD history, a minority of women had anthracyclines in the neo-adjuvant setting (*n* = 285, 13%); these tended to be younger, pre-menopausal, of higher socio-economic status, diagnosed with hormone-receptor positive cancers and at more advanced disease, compared to women with CVD that had anthracyclines in the adjuvant setting only (Supplementary Table 9). A slightly higher proportion of women without CVD at diagnosis used anthracyclines in the neo-adjuvant setting (*n* = 1,430, 16.5%); the patterns of anthracycline use were similar to those described for women with history of CVD. There were no meaningful differences in the patient characteristics with and without CVD that received anthracyclines by molecular subtype (Supplementary Table 10).

**Table 2:** Characteristics of patients with and without prior CVD that received/did not receive anthracyclines

	Breast cancer patients with a history of CVD at diagnosis^a (^*N* = 10,702)		Breast cancer patients with no history of CVD at diagnosis^a^ (*N* = 21,888)	
	No anthracyclines (*N* = 8,533, 79.7%)	Anthracyclines (*N* = 2,169, 20.3%)	No anthracyclines (*N* = 13,234, 60.5%)	Anthracyclines (*N* = 8,654, 39.5%)
Type of cardiovascular disease, *n* (%)				
CAD	1,661 (19.5)	198 (9.1)	0 (0.0)	0 (0.0)
Heart failure	1,110 (13.0)	42 (1.9)	0 (0.0)	0 (0.0)
Hypertension	5,397 (63.2)	1,059 (48.8)	0 (0.0)	0 (0.0)
Stroke	1,009 (11.8)	131 (6.0)	0 (0.0)	0 (0.0)
Demographics				
Age at diagnosis (years)				
Mean (SD)	73.8 (11.6)	61.1 (10.2)	63.2 (12.8)	53.7 (11.4)
Median (IQR)	74.0 (67.0, 83.0)	63.0 (54.0, 69.0)	64.0 (53.0, 72.0)	54.0 (45.0, 63.0)
Age group at diagnosis (years), *n* (%)				
18 to 39	26 (0.3)	75 (3.5)	326 (2.5)	976 (11.3)
40 to 59	884 (10.4)	740 (34.1)	4,630 (35.0)	4,749 (54.9)
60 to 79	4,609 (54.0)	1,335 (61.5)	6,772 (51.2)	2,913 (33.7)
80 plus	3,014 (35.3)	19 (0.9)	1,506 (11.4)	16 (0.2)
Year of breast cancer diagnosis, *n* (%)				
2010 to 2011	2,215 (26.0)	444 (20.5)	4,184 (31.6)	2,068 (23.9)
2012 to 2013	2,780 (32.6)	753 (34.7)	3,971 (30.0)	3,115 (36.0)
2014 to 2015	3,538 (41.5)	972 (44.8)	5,079 (38.4)	3,471 (40.1)
Region of Sweden, *n* (%)				
North	644 (7.5)	206 (9.5)	930 (7.0)	709 (8.2)
South	1,604 (18.8)	427 (19.7)	2,362 (17.8)	1,618 (18.7)
Southeast	834 (9.8)	243 (11.2)	1,230 (9.3)	886 (10.2)
Stockholm/Gotland	1,720 (20.2)	546 (25.2)	2,562 (19.4)	2,313 (26.7)
Uppsala/Örebro	1,887 (22.1)	386 (17.8)	3,124 (23.6)	1,529 (17.7)
West	1,844 (21.6)	361 (16.6)	3,026 (22.9)	1,599 (18.5)
Civil status, *n* (%)				
Unmarried	743 (8.7)	303 (14.0)	2,180 (16.5)	2,077 (24.0)
Married	3,601 (42.2)	1,227 (56.6)	6,839 (51.7)	4,729 (54.6)
Divorced/separated	1,540 (18.0)	455 (21.0)	2,452 (18.5)	1,472 (17.0)
Widow	2,649 (31.0)	184 (8.5)	1,760 (13.3)	373 (4.3)
Unknown	743 (8.7)	303 (14.0)	3 (0.0)	3 (0.0)
Level of education, *n* (%)				
Compulsory education or less	3,269 (38.3)	409 (18.9)	3,047 (23.0)	1,173 (13.6)
Upper secondary	3,243 (38.0)	1,006 (46.4)	5,468 (41.3)	3,677 (42.5)
College/University/Research	1,915 (22.4)	740 (34.1)	4,597 (34.7)	3,762 (43.5)
Unknown	106 (1.2)	14 (0.6)	122 (0.9)	42 (0.5)
Disposable income SEK, *n* (%)				
1 to 1,000	1,214 (14.2)	207 (9.5)	1,336 (10.1)	609 (7.0)
1,001 to 2,000	5,380 (63.0)	953 (43.9)	5,915 (44.7)	2,736 (31.6)
2,001 to 3,000	1,226 (14.4)	649 (29.9)	3,737 (28.2)	3,290 (38.0)
> 3,000	697 (8.2)	351 (16.2)	2,137 (16.1)	1,911 (22.1)
Unknown	16 (0.2)	9 (0.4)	109 (0.8)	108 (1.2)
Tumour characteristics, *n* (%)				
TNM stage				
Stage 1	4,635 (54.3)	874 (40.3)	8,878 (67.1)	3,578 (41.3)
Stage 2	3,533 (41.4)	1,164 (53.7)	4,022 (30.4)	4,543 (52.5)
Stage 3	365 (4.3)	131 (6.0)	334 (2.5)	533 (6.2)
Histological grade				
Grade 1	1,573 (18.4)	75 (3.5)	3,256 (24.6)	337 (3.9)
Grade 2	3,773 (44.2)	712 (32.8)	6,736 (50.9)	2,806 (32.4)
Grade 3	1,580 (18.5)	1,079 (49.7)	2,081 (15.7)	4,018 (46.4)
Unknown	1,607 (18.8)	303 (14.0)	1,161 (8.8)	1,493 (17.3)
HER2 status				
Negative	6,580 (77.1)	1,535 (70.8)	10,840 (81.9)	6,197 (71.6)
Positive	544 (6.4)	556 (25.6)	705 (5.3)	2,161 (25.0)
Unknown/unrecorded	1,409 (16.5)	78 (3.6)	1,689 (12.8)	296 (3.4)
ER status				
Negative	6,887 (80.7)	1,527 (70.4)	11,297 (85.4)	6,307 (72.9)
Positive	685 (8.0)	621 (28.6)	779 (5.9)	2,238 (25.9)
Unknown	961 (11.3)	21 (1.0)	1,158 (8.8)	109 (1.3)
PR status				
Negative	5,852 (68.6)	1,219 (56.2)	9,802 (74.1)	5,149 (59.5)
Positive	1,708 (20.0)	922 (42.5)	2,257 (17.1)	3,387 (39.1)
Unknown	973 (11.4)	28 (1.3)	1,175 (8.9)	118 (1.4)
Menopausal status				
Pre-menopausal	334 (3.9)	364 (16.8)	2,487 (18.8)	3,404 (39.3)
Post-menopausal	7,825 (91.7)	1,611 (74.3)	9,682 (73.2)	4,458 (51.5)
Unknown	374 (4.4)	194 (8.9)	1065 (8.0)	792 (9.2)
Comorbidities at breast cancer diagnosis, *n* (%)				
Diabetes	1,390 (16.3)	270 (12.4)	563 (4.3)	277 (3.2)
CKD	196 (2.3)	12 (0.6)	15 (0.1)	7 (0.1)
COPD	588 (6.9)	67 (3.1)	216 (1.6)	69 (0.8)
Breast cancer treatment in the year after diagnosis, *n* (%)				
Surgery				
Primary operation	7,144 (83.7)	1,870 (86.2)	12,536 (94.7)	7,195 (83.1)
Pre-op oncological or conservative treatment	876 (10.3)	298 (13.7)	467 (3.5)	1,446 (16.7)
No surgery	510 (6.0)	1 (0.0)	229 (1.7)	13 (0.2)
Unknown	3 (0.0)	0 (0.0)	2 (0.0)	0 (0.0)
Chemotherapy				
Anthracyclines	0 (0.0)	2,169 (100.0)	0 (0.0)	8,654 (100.0)
Docetaxel	30 (0.4)	508 (23.4)	60 (0.5)	2,094 (24.2)
Paclitaxel	58 (0.7)	227 (10.5)	101 (0.8)	667 (7.7)
Other chemotherapy	77 (0.9)	86 (4.0)	136 (1.0)	337 (3.9)
Antibody therapy				
Trastuzumab	119 (1.4)	556 (25.6)	252 (1.9)	2,222 (25.7)
Pertuzumab	0 (0.0)	2 (0.1)	0 (0.0)	5 (0.1)
Endocrine therapy				
Outpatient tamoxifen	2,527 (29.6)	408 (18.8)	5,924 (44.8)	3,094 (35.8)
Outpatient GNRH	36 (0.4)	55 (2.5)	144 (1.1)	452 (5.2)
Outpatient AI	4,861 (57.0)	1,196 (55.1)	5,028 (38.0)	3,599 (41.6)
Radiotherapy				
Not recorded	4,780 (56.0)	390 (18.0)	5,339 (40.3)	1,422 (16.4)
Right breast	1,847 (21.6)	857 (39.5)	3,851 (29.1)	3,614 (41.8)
Left breast	1,906 (22.3)	922 (42.5)	4,044 (30.6)	3,618 (41.8)

*AI* aromatase inhibitors, *COPD* chronic obstructive pulmonary disease, *CKD* chronic kidney disease, *CAD* coronary artery disease, *ER* oestrogen receptor, *GNRH* gonadotropin-releasing hormone, *HER2* human epidermal growth factor receptor 2, *IQR* interquartile range, *n/a* not applicable, *PR* progesterone receptor, *SD* standard deviation, *TNM* UICC TNM Classification of Malignant Tumours

^a^CVD history defined as having one or more of the following conditions at breast cancer diagnosis: coronary heart disease (CAD), heart failure, primary hypertension and stroke

Table 3 describes the characteristics of those diagnosed with HER2+ tumours by history of CVD and trastuzumab treatment. Supplementary tables 11 to 16 provide characteristics for each type of CVD. In both women with and without history of CVD, trastuzumab was more often used in younger women and in those of higher socio-economic status. The were no meaningful differences between the patients that received trastuzumab in the neoadjuvant and adjuvant settings (Supplementary Table 17).

**Table 3:** Patient and tumour characteristics of the 3,966 patients with HER2 + tumours, and without prior CVD that received/did not receive trastuzumab

	HER2 + breast cancer patients with CVD^a^ at diagnosis (*N* = 1,100)		HER2 + breast cancer patients without CVD^a^ at diagnosis (*N* = 2,866)	
	No trastuzumab (*N* = 470, 42.7%)	Trastuzumab (*N* = 630, 57.3%)	No trastuzumab (*N* = 587, 20.5%)	Trastuzumab (*N* = 2,279, 79.5%)
Type of cardiovascular disease, *n* (%)				
CAD	109 (23.2)	64 (10.2)	0 (0.0)	0 (0.0)
Heart failure	63 (13.4)	22 (3.5)	0 (0.0)	0 (0.0)
Hypertension	326 (69.4)	302 (47.9)	0 (0.0)	0 (0.0)
Stroke	68 (14.5)	37 (5.9)	0 (0.0)	0 (0.0)
Demographics				
Age at diagnosis (years)				
Mean (SD)	76.5 (10.9)	62.6 (10.4)	61.9 (15.3)	54.1 (12.0)
Median (IQR)	78.0 (70.0, 84.0)	64.0 (56.0, 70.0)	62.0 (50.0, 72.0)	54.0 (46.0, 63.0)
Age group at diagnosis (years),*n* (%)				
18 to 39	1 (0.2)	19 (3.0)	45 (7.7)	279 (12.2)
40 to 59	33 (7.0)	192 (30.5)	219 (37.3)	1,217 (53.4)
60 to 79	228 (48.5)	398 (63.2)	225 (38.3)	763 (33.5)
80 plus	208 (44.3)	21 (3.3)	98 (16.7)	20 (0.9)
Year of breast cancer diagnosis,*n* (%)				
2010 to 2011	152 (32.3)	131 (20.8)	271 (46.2)	543 (23.8)
2012 to 2013	129 (27.4)	199 (31.6)	119 (20.3)	809 (35.5)
2014 to 2015	189 (40.2)	300 (47.6)	197 (33.6)	927 (40.7)
Region of Sweden, *n* (%)				
North	34 (7.2)	48 (7.6)	37 (6.3)	203 (8.9)
South	80 (17.0)	118 (18.7)	71 (12.1)	421 (18.5)
Southeast	46 (9.8)	62 (9.8)	70 (11.9)	241 (10.6)
Stockholm/Gotland	58 (12.3)	155 (24.6)	73 (12.4)	571 (25.1)
Uppsala/Örebro	154 (32.8)	120 (19.0)	210 (35.8)	402 (17.6)
West	98 (20.9)	127 (20.2)	126 (21.5)	441 (19.4)
Civil status,*n* (%)				
Unmarried	39 (8.3)	85 (13.5)	120 (20.4)	561 (24.6)
Married	170 (36.2)	357 (56.7)	268 (45.7)	1,213 (53.2)
Divorced/separated	87 (18.5)	116 (18.4)	103 (17.5)	392 (17.2)
Widow	174 (37.0)	72 (11.4)	96 (16.4)	113 (5.0)
Unknown	39 (8.3)	85 (13.5)	0 (0.0)	0 (0.0)
Level of education, *n* (%)				
Compulsory education or less	202 (43.0)	134 (21.3)	137 (23.3)	312 (13.7)
Upper secondary	166 (35.3)	296 (47.0)	258 (44.0)	978 (42.9)
College/University/Research	100 (21.3)	195 (31.0)	190 (32.4)	976 (42.8)
Unknown	2 (0.4)	5 (0.8)	2 (0.3)	13 (0.6)
Disposable income SEK, *n* (%)				
1 to 1,000	71 (15.1)	62 (9.8)	65 (11.1)	151 (6.6)
1,001 to 2,000	322 (68.5)	287 (45.6)	288 (49.1)	731 (32.1)
2,001 to 3,000	49 (10.4)	177 (28.1)	157 (26.7)	857 (37.6)
> 3,000	25 (5.3)	100 (15.9)	71 (12.1)	511 (22.4)
Unknown	3 (0.6)	4 (0.6)	6 (1.0)	29 (1.3)
Tumour characteristics, *n* (%)				
TNM stage				
Stage 1	172 (36.6)	254 (40.3)	266 (45.3)	938 (41.2)
Stage 2	263 (56.0)	336 (53.3)	284 (48.4)	1,175 (51.6)
Stage 3	35 (7.4)	40 (6.3)	37 (6.3)	166 (7.3)
Histological grade				
Grade 1	13 (2.8)	13 (2.1)	56 (9.5)	39 (1.7)
Grade 2	148 (31.5)	153 (24.3)	188 (32.0)	553 (24.3)
Grade 3	257 (54.7)	365 (57.9)	277 (47.2)	1,217 (53.4)
Unknown	52 (11.1)	99 (15.7)	66 (11.2)	470 (20.6)
ER status				
Negative	329 (70.0)	395 (62.7)	430 (73.3)	1,498 (65.7)
Positive	141 (30.0)	235 (37.3)	157 (26.7)	781 (34.3)
Unknown	0 (0.0)	0 (0.0)	0 (0.0)	0 (0.0)
PR status				
Negative	233 (49.6)	266 (42.2)	318 (54.2)	1,104 (48.4)
Positive	237 (50.4)	364 (57.8)	269 (45.8)	1,175 (51.6)
Unknown	0 (0.0)	0 (0.0)	0 (0.0)	0 (0.0)
Menopausal status				
Pre-menopausal	17 (3.6)	84 (13.3)	135 (23.0)	842 (36.9)
Post-menopausal	437 (93.0)	489 (77.6)	407 (69.3)	1,209 (53.0)
Unknown	16 (3.4)	57 (9.1)	45 (7.6)	228 (10.0)
Comorbidities at breast cancer diagnosis, *n* (%)				
Diabetes	90 (19.1)	87 (13.8)	35 (6.0)	71 (3.1)
CKD	8 (1.7)	4 (0.6)	0 (0.0)	0 (0.0)
COPD	38 (8.1)	17 (2.7)	8 (1.4)	21 (0.9)
Breast cancer treatment in the year after diagnosis, *n* (%)				
Surgery				
Primary operation	422 (89.8)	534 (84.8)	539 (91.8)	1,817 (79.7)
Pre-op oncological or conservative treatment	27 (5.7)	95 (15.1)	34 (5.8)	458 (20.1)
No surgery	21 (4.5)	1 (0.2)	13 (2.2)	4 (0.2)
Unknown	427 (89.9)	0 (0.0)	1 (0.2)	0 (0.0)
Chemotherapy				
Anthracyclines	37 (7.9)	519 (82.4)	119 (20.3)	2,042 (89.6)
Docetaxel	6 (1.3)	145 (23.0)	26 (4.4)	563 (24.7)
Paclitaxel	3 (0.6)	88 (14.0)	8 (1.4)	207 (9.1)
Other chemotherapy	7 (1.5)	55 (8.7)	12 (2.0)	125 (5.5)
Antibody therapy				
Trastuzumab	0 (0.0)	630 (100.0)	0 (0.0)	2,279 (100.0)
Pertuzumab	0 (0.0)	2 (0.3)	1 (0.2)	4 (0.2)
Endocrine therapy				
Outpatient tamoxifen	64 (13.6)	85 (13.5)	169 (28.8)	707 (31.0)
Outpatient GNRH	0 (0.0)	9 (1.4)	11 (1.9)	122 (5.4)
Outpatient AI	278 (59.1)	313 (49.7)	233 (39.7)	886 (38.9)
Radiotherapy				
Not recorded	319 (67.9)	155 (24.6)	347 (59.1)	482 (21.1)
Right breast	71 (15.1)	234 (37.1)	129 (22.0)	873 (38.3)
Left breast	80 (17.0)	241 (38.3)	111 (18.9)	924 (40.5)

*AI* aromatase inhibitors, *COPD* chronic obstructive pulmonary disease, *CKD* chronic kidney disease, *CAD* coronary artery disease, *ER* oestrogen receptor, *GNRH* gonadotropin-releasing hormone, *HER2* human epidermal growth factor receptor 2, *IQR* interquartile range, *n/a* not applicable, *PR* progesterone receptor, *SD* standard deviation, *TNM* UICC TNM Classification of Malignant Tumours

^a^CVD history defined as having one or more of the following conditions at breast cancer diagnosis coronary heart disease (CAD), heart failure, primary hypertension and stroke

### Associations between anthracycline and trastuzumab use and history of CVD, overall and by subtype of breast cancer

**Fig. 1:**
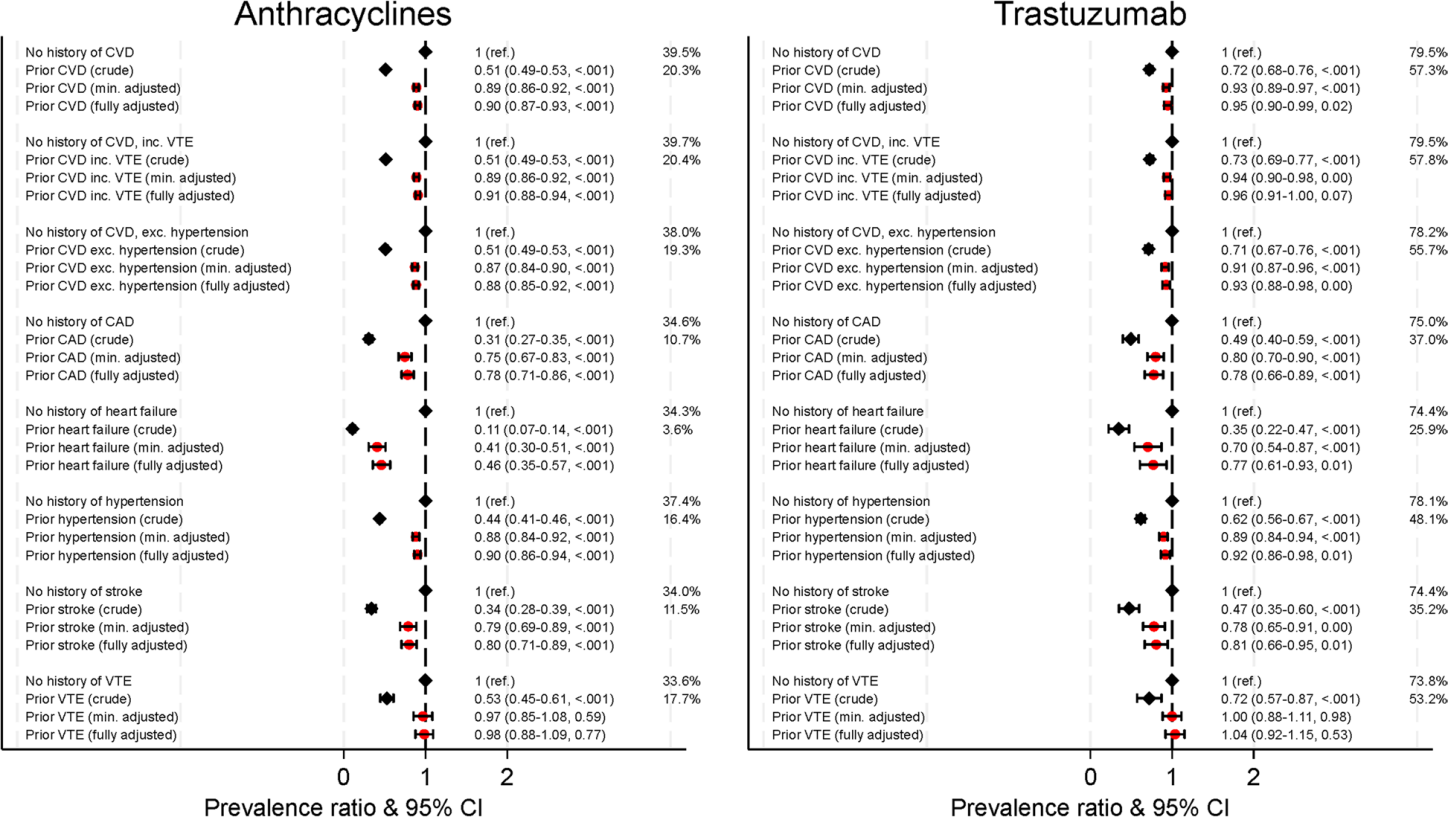
Association between cardiovascular diseases and anthracyclines and trastuzumab† use. Min. adjusted = Prevalence ratios adjusted for age (5-year age groups). Fully adjusted = Prevalence ratios adjusted for age (5-year age groups), region, socio-economic variables (education, civil status, and disposable income), comorbidities (chronic kidney disease, chronic obstructive pulmonary disease and diabetes), and tumour-related variables (stage at diagnosis, HER2 receptor status for anthracyclines, tumour grade and diagnostic subtype). † Analyses of trastuzumab include only breast cancer patients with HER2 + tumours. Crude prevalences are shown. CVD = cardiovascular disease, includes coronary heart disease (CAD), heart failure, primary hypertension and stroke; CAD = coronary artery disease; CI = confidence interval; Ref. = reference; VTE = venous thromboembolism

Figure 1 shows the crude and adjusted prevalence ratios of anthracycline and trastuzumab use in breast cancer patients with history of CVD, compared to those with no history of CVD. The prevalences of anthracycline use were lower in women with prior CVD compared with women without prior CVD, but associations varied by CVD type; crude prevalence ratios ranged from 0.11 (0.07–0.14) for heart failure, to 0.53 (0.45–0.61) for history of VTE. Adjusting for age resulted in weaker associations in all instances, and prior history of VTE was no longer associated with differences in anthracycline use. Further adjustment for socio-demographics, tumour characteristics and other comorbidities did not meaningfully change the associations. In fully adjusted models, prevalence ratios ranged from 0.46 (0.35–0.57) for heart failure to 0.98 (0.88–1.09) for history of VTE.

Among women with HER2+ breast cancer, trastuzumab was used in 57.8% of women with any prior CVD, in 25.9% of women with heart failure, 37.0% of those with CAD, 48.1% with hypertension, 35.2% with stroke, and 53.2% with history of VTE (Fig. 1). Again, these prevalences were lower than among women without prior CVD. Crude prevalence ratios ranged from 0.35 (0.22–0.47) for heart failure to 0.72 (0.57–0.87) for VTE. Age adjustment attenuated the prevalence ratios, with further adjustment having little effect; fully adjusted prevalence ratios ranged from 0.77 (0.61–0.93) for heart failure to 1.04 (0.92–1.15) for VTE.

**Fig. 2:**
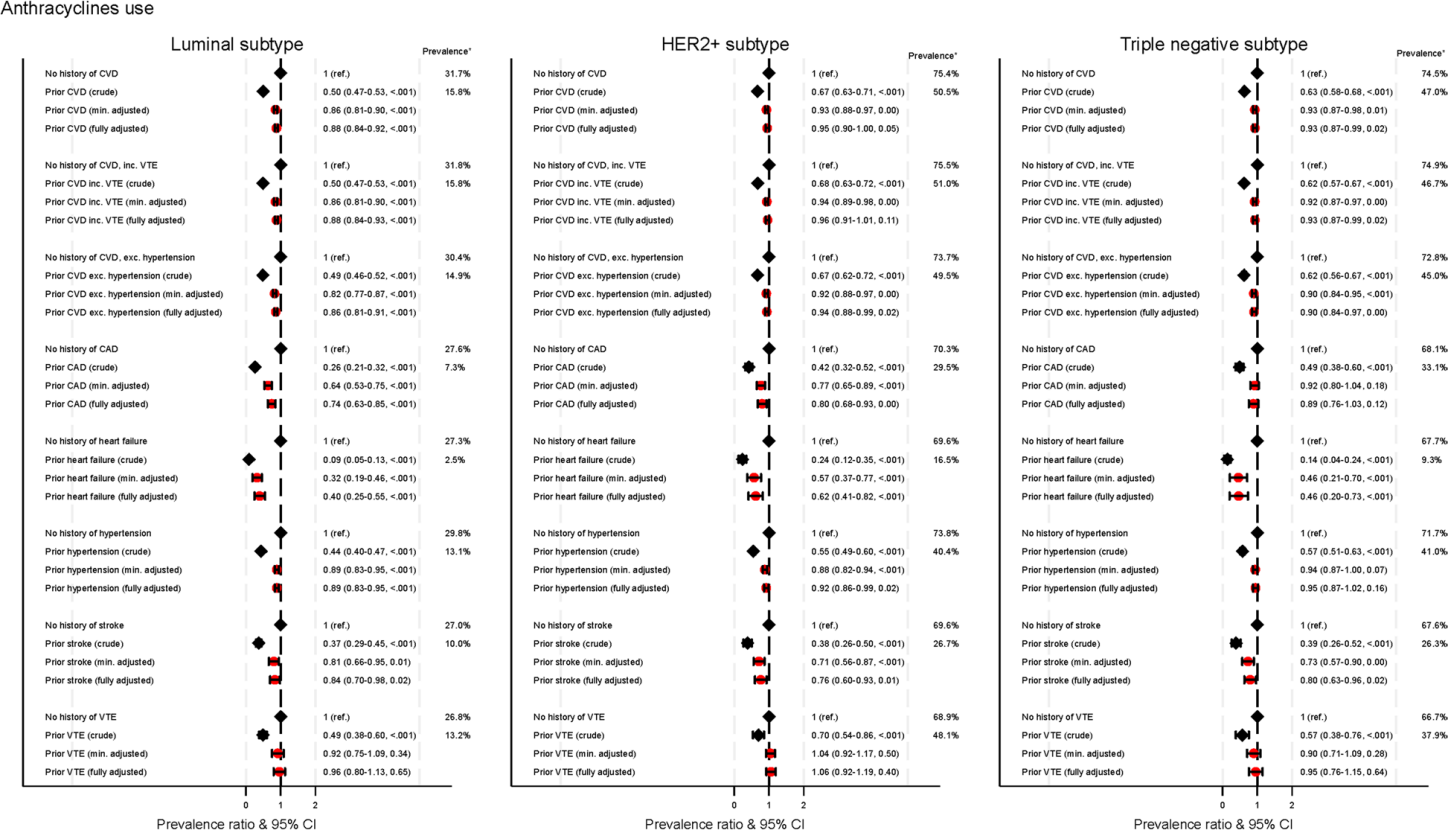
Association between cardiovascular diseases and anthracyclines use stratified by subtype of breast cancer. Min. adjusted = Prevalence ratios adjusted for age (5-year age groups). Fully adjusted = Prevalence ratios adjusted for age (5-year age groups), region, socio-economic variables (education, civil status, and disposable income), comorbidities (chronic kidney disease, chronic obstructive pulmonary disease and diabetes), and tumour-related variables (stage at diagnosis, HER2 receptor status for anthracyclines, tumour grade and diagnostic subtype). * Crude prevalences are shown. CVD = cardiovascular disease, includes coronary heart disease (CAD), heart failure, primary hypertension and stroke; CAD = coronary artery disease; CI = confidence interval; Ref. = reference; VTE = venous thromboembolism

Figure 2 shows the association between cardiovascular diseases and anthracyclines use stratified by subtype of breast cancer. In all types, women with CVD had lower prevalence of anthracycline use compared to women without CVD. Women with luminal cancer had the lowest use of anthracyclines (15.8% for CVD history vs 31.7% for no CVD). Use of anthracyclines was higher among those with HER2 + (50.5% for CVD history vs 75.4% for no CVD) and triple negative (47.0% for CVD history vs 74.5% for no CVD) cancers. In fully adjusted models, the prevalence ratio for the association between history of CVD (overall) and anthracycline use was similar across subtypes, ranging from 0.88 (0.84–0.92) for luminal cancer to 0.95 (0.87–0.99) for triple negative tumours. The association for types of CVD varied by cancer subtype: a history of CAD and a history of hypertension were associated with lower anthracycline use in luminal and HER2 + cancers, but not in triple negative cancer (0.89, 0.76–1.03); a history of stroke and heart failure was significantly associated with lower anthracycline use in all subtypes; and a history of VTE had no impact on anthracycline use for any subtype.

There were no meaningful differences in the association between history of CVD in breast patients and use of anthracyclines and trastuzumab in the neo-adjuvant or adjuvant setting (Supplementary Figs. 2 and 3).

### Use of breast cancer treatments by history of CVD over calendar time

**Fig. 3:**
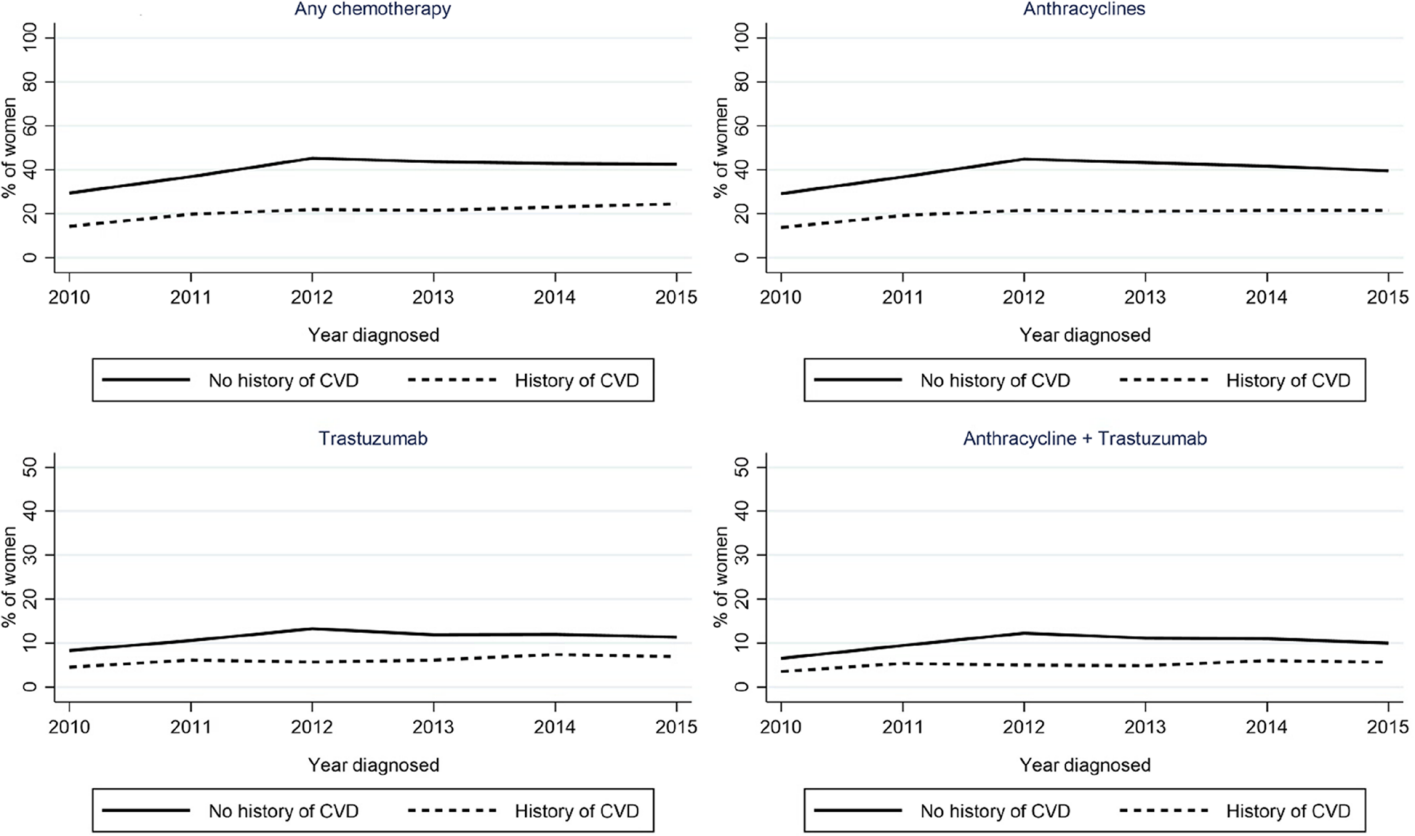
Proportion of breast cancer patients treated with chemotherapy, anthracyclines, trastuzumab, and both an anthracycline and trastuzumab, by history of cardiovascular disease, between 210 and 2015. Prevalence of trastuzumab use among all breast cancers, for comparison purposes; treatment categories not mutually exclusive

Figure 3 shows the proportion of breast cancer patients treated with chemotherapy, anthracyclines, trastuzumab, and both an anthracycline and trastuzumab, by history of CVD over time. Approximately 40% of the breast cancer patients with no prior CVD received chemotherapy, compared to ~ 20% of those with history of CVD. Trastuzumab was also more commonly used in those with no history of CVD than in those without. During the study period, there was a modest increase in the proportion of women treated with anthracyclines and trastuzumab both in women with and without CVD.

## Discussion

### Summary of key findings

This study described the real-world use of anthracyclines and trastuzumab in women diagnosed with non-metastatic breast cancer in 2010–15 in Sweden, providing the first comprehensive picture of how cardiotoxic breast cancer therapies are being used in patients with pre-existing CVD in routine clinical practice. We showed that one in five women who had a history of CVD at breast cancer diagnosis during this period received anthracyclines as part of their treatment, and one in two women with CVD and HER2 tumours received trastuzumab. As expected, history of CVD was associated with lower use of anthracyclines and trastuzumab, but the extent varied largely by type of pre-existing CVD. Women with heart failure were the least likely to receive anthracyclines and trastuzumab, while history of VTE had little impact. Anthracyclines were more often used in women with CVD if these had triple negative or HER2+ tumours. Both in women with and without CVD, cardiotoxic drugs were more commonly used in younger women and in those of higher socio-economic status.

### Discussion of findings in the context of previous literature

While patients with comorbid CVD were generally less likely to receive anthracyclines and trastuzumab, in line with recommendations for anthracycline avoidance in high risk patients [5, 6], the findings reveal considerable nuance in prescribing patterns. This shows that clinicians implemented some form of risk stratification in decision-making, rather than categorically excluding all CVD patients from these treatments. The varying patterns of use across different cardiovascular conditions suggest clinicians are weighing specific cardiovascular risks against the potential benefits of these highly effective cancer therapies, aligned with recent recommendations for individualised decision-making [7].

However, the substantial variation observed also raises questions about whether current practice patterns are consistent and optimal, and whether some patient groups might be unnecessarily excluded from effective treatments while others might be exposed to excessive risk.

The observed variation in use of anthracyclines and trastuzumab by type of CVD is expected given that clinical decisions are based on the risk–benefit ratio of the cancer and the side effects of its treatment, and the potential benefits in terms of survival and mortality. We excluded history of VTE from our main composite CVD outcome, for consistency with prior literature [16]. However, we also investigated the role of VTE history as a single exposure and by including in the composite CVD outcome, as studies suggested a substantial burden of thromboembolism in breast cancer patients treated with anthracyclines [17]. We found that VTE did not impact on the use of anthracyclines and trastuzumab, which may be explained by the regular use low molecular weight heparin or direct oral anticoagulants for prevention of thromboembolic events in breast cancer patients [7, 18], which likely eases concerns about subsequent events. Hypertension also had little impact on the use of cardiotoxic drugs, which is compatible with the asymptomatic nature of the condition and potential normal cardiac function. Although at the time such studies were still in early phases, there is now some evidence that use of angiotensin converting enzyme inhibitors (ACE inhibitors), angiotensin II receptor blockers (ARBs), or beta-blockers as cardioprotective drugs during trastuzumab treatments, and possibly during anthracyclines, attenuate declines in left ventricular ejection fraction [19]. The current study did not contain data on hypertension severity or blood pressure monitoring. A recent study showed that women with severe hypertension receiving chemotherapy for breast cancer had a steeper decline in cardiac function, compared to women with normal blood pressure levels [20]. Women with history of stroke, CAD and heart failure were less likely to have trastuzumab and anthracyclines, compatible with the likelihood of non-reversible cardiac dysfunction of these drugs. We did not have further data on the use of cardioprotective drugs during treatment for patients with history of CVD.

### Implications of this study for research

This study provides essential data on the real-world prescribing patterns of potentially cardiotoxic breast cancer therapies in patients with pre-existing CVD. The substantial number of patients with CVD receiving these treatments, along with the marked variation in use across different cardiovascular conditions, highlight both the feasibility of treating selected high-risk patients, and the need for additional evidence to guide consistent practice. Our findings demonstrate that follow up data have been accumulating on women with CVD receiving these treatments; this should enable the important next step of investigating subsequent cardiovascular and cancer outcomes in such patients. Our study also suggests a need for application of specific guidance to aid treatment decisions in this clinical scenario (which did not exist at the time [7]), and evaluation of whether current prescribing patterns adequately optimise the balance between cancer outcomes and cardiovascular risk. This evidence will be crucial for moving towards more evidence-based individualised decision-making in this complex patient population.

### Strengths and limitations

Strengths of this study include the population-based nature of the data, which include virtually all patients diagnosed with breast cancer in Sweden during 2010–2015. The data resources used for this study are well established, subject to extensive quality controls, and regarded of very good quality for research. The comprehensiveness of the information collected allowed us to describe in detail the associations between history of several cardiovascular outcomes and use of systemic treatments for breast cancer, while accounting for important variables such as socio-economic indicators. However, this study has limitations. Importantly, the lack of data on the decision-making process and specific factors that influenced treatment choices in individual cases. This limits our ability to assess whether observed patterns reflect appropriate risk stratification or potential over or under-use of these therapies. We estimated associations separately for the neo-adjuvant and adjuvant setting, however, the small number of patients with breast cancer and CVD that received anthracyclines or trastuzumab in the neo-adjuvant setting should warrant caution in the interpretation of these results. Data on comorbidities were ascertained from hospital in-patient data, which is likely incomplete for conditions that are primarily managed in primary care, such as diabetes and hypertension. We used antidiabetic prescriptions to supplement our definition of diabetes, which is likely to have minimised the information bias in this variable. For hypertension, however, the myriad of indications of most anti-hypertensive drugs precluded a similar approach. Although the quality of the data is very good, some misclassification of anthracycline exposure may have occurred, as previous studies showed that some postoperative chemotherapy with anthracyclines are missed [8]. There were also some records with missing HER2, PR, ER status. Some missingness for these variables is plausible, as sometimes this is not measured and thus is unknown. It is also possible that these variables were measured but not recorded (particularly for HER2 as results arrive later and the clinician needs to enter these in the system [8]). Finally, we did not have data on other indicators of cardiac function that might have been used in the decision to administer anthracyclines and trastuzumab to women with pre-existing CVD.

## Conclusion

Women diagnosed with non-metastatic breast cancer in 2010–2015 in Sweden were less likely to be treated with anthracyclines and trastuzumab if they had comorbid CVD. However, one in five women with comorbid CVD received anthracyclines, and one in two women with CVD and HER2+ breast cancer had trastuzumab, with substantial variation by type of CVD. These findings provide an important foundation for future research to optimise patient selection and inform evidence-based guidance on the use of these effective but potentially cardiotoxic therapies in patients with pre-existing CVD.

## Supplementary Information

Supplementary Material 1: Supplementary Table 1. Description of the data sources. Supplementary Table 2. Definition of the study variables. Supplementary Table 3. Patient and tumour characteristics of patients with and without prior coronary artery disease (CAD) that received/did not receive anthracyclines. Supplementary Table 4. Patient and tumour characteristics of patients with and without prior heart failure that received/did not receive anthracyclines>. Supplementary Table 5. Patient and tumour characteristics of patients with and without prior hypertension that received/did not receive anthracyclines. Supplementary Table 6. Patient and tumour characteristics of patients with and without prior strokethat received/did not receive anthracyclines. Supplementary Table 7. Patient and tumour characteristics of patients with and without prior venous thromboembolism (VTE) that received/did not receive anthracyclines. Supplementary Table 8. Patient and tumour characteristics of patients with and without prior excluding hypertension that received/did not receive anthracyclines. Supplementary Table 9.Characteristics of patients with and without prior CVD that received/did not receive anthracyclines, stratified by neo-adjuvant and adjuvant setting. Supplementary Table 10. Characteristics of patients with and without prior CVD that received/did not receive anthracyclines, stratified by breast cancer subtype. Supplementary Table 11. Patient and tumour characteristics of patients with and without prior coronary artery disease (CAD) that received/did not receive trastuzumab. Supplementary Table 12. Patient and tumour characteristics of patients with and without prior heart failure that received/did not receive trastuzumab. Supplementary Table 13. Patient and tumour characteristics of patients with and without prior hypertension that received/did not receive trastuzumab. Supplementary Table 14. Patient and tumour characteristics of patients with and without prior strokethat received/did not receive trastuzumab. Supplementary Table 15. Patient and tumour characteristics of patients with and without prior venous thromboembolism (VTE) that received/did not receive trastuzumab. Supplementary Table 16. Patient and tumour characteristics of patients with and without prior CVD excluding hypertension that received/did not receive trastuzumab. Supplementary Table 17. Patient and tumour characteristics of the 3,966 patients with HER2+ tumours, and without prior CVD that received/did not receive trastuzumab, stratified by neo-adjuvant and adjuvant setting. Supplementary Figure 1. Selection of study population. Supplementary Figure 2 Association between cardiovascular diseases and anthracyclines use stratified by neo-adjuvant and adjuvant setting. Supplementary Figure 3 Association between cardiovascular diseases and trastuzumab use stratified by neo-adjuvant and adjuvant setting.

## Data Availability

No datasets were generated or analysed during the current study.
