# Supplementary material for: Use of anthracyclines and trastuzumab for breast cancer in women with and without a history of cardiovascular disease in Sweden: a national cross-sectional study
Source: Cardiooncology. 2025 Jun 20;11:56. doi: 10.1186/s40959-025-00356-z (PMC12180225; doi:10.1186/s40959-025-00356-z)
Supplement: Supplementary file 1 — Supplementary Material 1: Supplementary Table 1. Description of the data sources. Supplementary Table 2. Definition of the study variables. Supplementary Table 3. Patient and tumour characteristics of patients with and without prior coronary artery disease (CAD) that received/did not receive anthracyclines. Supplementary Table 4. Patient and tumour characteristics of patients with and without prior heart failure that received/did not receive anthracyclines>. Supplementary Table 5. Patient and tumour characteristics of patients with and without prior hypertension that received/did not receive anthracyclines. Supplementary Table 6. Patient and tumour characteristics of patients with and without prior strokethat received/did not receive anthracyclines. Supplementary Table 7. Patient and tumour characteristics of patients with and without prior venous thromboembolism (VTE) that received/did not receive anthracyclines. Supplementary Table 8. Patient and tumour characteristics of patients with and without prior excluding hypertension that received/did not receive anthracyclines. Supplementary Table 9.Characteristics of patients with and without prior CVD that received/did not receive anthracyclines, stratified by neo-adjuvant and adjuvant setting. Supplementary Table 10. Characteristics of patients with and without prior CVD that received/did not receive anthracyclines, stratified by breast cancer subtype. Supplementary Table 11. Patient and tumour characteristics of patients with and without prior coronary artery disease (CAD) that received/did not receive trastuzumab. Supplementary Table 12. Patient and tumour characteristics of patients with and without prior heart failure that received/did not receive trastuzumab. Supplementary Table 13. Patient and tumour characteristics of patients with and without prior hypertension that received/did not receive trastuzumab. Supplementary Table 14. Patient and tumour characteristics of patients with and without prior strokethat rec [file 40959_2025_356_MOESM1_ESM.docx]

**Supplementary file**

**Paper: Use of anthracyclines and trastuzumab for breast cancer in women with and without a history of cardiovascular disease in Sweden: a national cross-sectional study**

**List of Supplementary tables and Figures**

[**Supplementary Table 1** Description of the data sources. 3](#_Toc199531807)

[**Supplementary Table 2**  Definition of the study variables. 5](#_Toc199531808)

[**Supplementary** **Table 3** Patient and tumour characteristics of patients with and without prior coronary artery disease (CAD) that received/did not receive anthracyclines. 6](#_Toc199531809)

[**Supplementary Table 4** Patient and tumour characteristics of patients with and without prior heart failure (HF) that received/did not receive anthracyclines. 8](#_Toc199531810)

[**Supplementary** **Table 5** Patient and tumour characteristics of patients with and without prior hypertension that received/did not receive anthracyclines. 10](#_Toc199531811)

[**Supplementary** **Table 6** Patient and tumour characteristics of patients with and without prior stroke that received/did not receive anthracyclines. 12](#_Toc199531812)

[**Supplementary** **Table 7** Patient and tumour characteristics of patients with and without prior venous thromboembolism (VTE) that received/did not receive anthracyclines. 14](#_Toc199531813)

[**Supplementary** **Table 8** Patient and tumour characteristics of patients with and without prior CVD, except for hypertension, that received/did not receive anthracyclines. (Please note that patients with hypertension as well as other CVDs are still included.) 16](#_Toc199531814)

[**Supplementary** **Table 9** Characteristics of patients with and without prior CVD that received/did not receive anthracyclines, stratified by neo-adjuvant and adjuvant setting. 18](#_Toc199531815)

[**Supplementary** **Table 10** Characteristics of patients with and without prior CVD that received/did not receive anthracyclines, stratified by breast cancer subtype. 21](#_Toc199531816)

[**Supplementary** **Table 11** Patient and tumour characteristics of patients with and without prior coronary artery disease (CAD) that received/did not receive trastuzumab. 23](#_Toc199531817)

[**Supplementary** **Table 12** Patient and tumour characteristics of patients with and without prior heart failure (HF) that received/did not receive trastuzumab. 25](#_Toc199531818)

[**Supplementary** **Table 13** Patient and tumour characteristics of patients with and without prior hypertension that received/did not receive trastuzumab. 27](#_Toc199531819)

[**Supplementary** **Table 14** Patient and tumour characteristics of patients with and without prior stroke that received/did not receive trastuzumab. 29](#_Toc199531820)

[**Supplementary** **Table 15** Patient and tumour characteristics of patients with and without prior venous thromboembolism (VTE) that received/did not receive trastuzumab. 31](#_Toc199531821)

[**Supplementary** **Table 16** Patient and tumour characteristics of patients with and without prior CVD, excluding hypertension, that received/did not receive trastuzumab. (Please note that patients with hypertension as well as other CVDs are still included.) 33](#_Toc199531822)

[**Supplementary** **Table** **17** Patient and tumour characteristics of the 3,966 patients with HER2+ tumours, and without prior CVD that received/did not receive trastuzumab, stratified by neo-adjuvant and adjuvant setting. 35](#_Toc199531823)

[**Supplementary** **Figure** **1** Selection of study population. 38](#_Toc199531824)

[**Supplementary** **Figure** **2** Association between cardiovascular diseases and anthracyclines use stratified by neo-adjuvant and adjuvant setting. 39](#_Toc199531825)

[**Supplementary** **Figure** **3** Association between cardiovascular diseases and trastuzumab use stratified by neo-adjuvant and adjuvant setting. 41](#_Toc199531826)

## Supplementary Table 1 Description of the data sources.

| ***Resource*** | ***Purpose*** | ***Time period*** |
| --- | --- | --- |
| National Quality Register for Breast Cancer [1] | National database covering diagnosis, treatment, and outcomes data for all primary invasive and *in situ* breast cancer cases.  To identify women with a history of unilateral invasive breast cancer, and to identify tumour characteristics (e.g.: stage/grade; oestrogen and progesterone receptor status, and human epidermal growth factor receptor 2 (HER-2) status), covariates (e.g.: menstrual status at diagnosis and time from diagnosis to treatment), and planned and delivered in hospital treatments (surgery, chemotherapy, and radiotherapy). | 2008-present |
| Swedish Prescribed Drug Register [2] | National register of drugs dispensed by pharmacies.  To identify dispensed prescriptions for drugs that are not used in hospitals or administered in day care in hospitals (e.g. tamoxifen). | 2005-present |
| Swedish Cause of Death Register [3] | National database of death certificates data including causes of death.  To identify fatal cardiovascular events and contributing cardiovascular causes, and to censor follow-up at death. | 1952-present |
| Swedish Total Population Register [4] | National register of Swedish population.  To define age at diagnosis, calculate follow-up time, and identify socioeconomic variables. | 1968-present |
| National Patient Register [5] | National dataset of hospitalisations.  To identify cardiovascular disease outcomes and other comorbidities. | 1987-present |
| Longitudinal integrated database for health insurance and labour market studies (LISA) [6] | Statistics Sweden longitudinal database that integrates existing data from the labour market, educational and social sectors and is updated each year with a new annual register.  To identify education and disposable income variables. | 1990-present |

1. Lofgren L, Eloranta S, Krawiec K, Asterkvist A, Lonnqvist C, Sandelin K, steering group of the National Register for Breast C: **Validation of data quality in the Swedish National Register for Breast Cancer**. *BMC Public Health* 2019, **19**(1):495.

2. Wallerstedt SM, Wettermark B, Hoffmann M: **The First Decade with the Swedish Prescribed Drug Register - A Systematic Review of the Output in the Scientific Literature**. *Basic Clin Pharmacol Toxicol* 2016, **119**(5):464-469.

3. Brooke HL, Talback M, Hornblad J, Johansson LA, Ludvigsson JF, Druid H, Feychting M, Ljung R: **The Swedish cause of death register**. *Eur J Epidemiol* 2017, **32**(9):765-773.

4. Ludvigsson JF, Almqvist C, Bonamy AK, Ljung R, Michaelsson K, Neovius M, Stephansson O, Ye W: **Registers of the Swedish total population and their use in medical research**. *Eur J Epidemiol* 2016, **31**(2):125-136.

5. Ludvigsson JF, Andersson E, Ekbom A, Feychting M, Kim JL, Reuterwall C, Heurgren M, Olausson PO: **External review and validation of the Swedish national inpatient register**. *BMC Public Health* 2011, **11**:450.

6. Ludvigsson JF, Svedberg P, Olen O, Bruze G, Neovius M: **The longitudinal integrated database for health insurance and labour market studies (LISA) and its use in medical research**. *Eur J Epidemiol* 2019, **34**(4):423-437.

## Supplementary Table 2 Definition of the study variables.

| **Variable** | **Definition** | **Use in this study** |
| --- | --- | --- |
| Chronic kidney disease | ICD10 code N18 | Covariate |
| Chronic obstructive pulmonary disease | ICD10 codes J41, J42, J43, J44 | Covariate |
| Coronary artery disease | ICD10 codes I20, I21, I46 | Exposure |
| Diabetes | ICD10 codes E10, E11, E12, E13, E14; ATC code A10 | Covariate |
| Heart failure | ICD10 codes I50, I255, I42, I43 | Exposure |
| Hypertension | ICD10 codes I10 | Exposure |
| Stroke | ICD10 codes I60, I61, I62, I63, I64 | Exposure |
| Venous thromboembolism | ICD10 codes I26, I801, I802, I803 | Exposure |

ICD = International Classification of Diseases, 10^th^ revision

ATC = Anatomical Therapeutic Chemical Classification System

## Supplementary Table 3 Patient and tumour characteristics of patients with and without prior coronary artery disease (CAD) that received/did not receive anthracyclines.

|  | **Breast cancer patients with a history of CAD at diagnosis** (N=1,859) | | |  | **Breast cancer patients with no history of CAD at diagnosis** (N=30,731) | | |
| --- | --- | --- | --- | --- | --- | --- | --- |
|  | **Anthracyclines**  (N=198, 10.7%) |  | **No anthracyclines**  (N=1,661, 89.3%) |  | **Anthracyclines**  (N=10,625, 34.6%) |  | **No anthracyclines**  (N=20,106, 65.4%) |
| **Type of cardiovascular disease*, n (%)*** | | | | |  |  |  |
| Heart failure | 18 (9.1) |  | 455 (27.4) |  | 24 (0.2) |  | 655 (3.3) |
| Hypertension | 111 (56.1) |  | 1,126 (67.8) |  | 948 (8.9) |  | 4,271 (21.2) |
| Stroke | 9 (4.5) |  | 181 (10.9) |  | 122 (1.1) |  | 828 (4.1) |
| VTE | 5 (2.5) |  | 100 (6.0) |  | 131 (1.2) |  | 532 (2.6) |
| **Demographics** |  |  |  |  |  |  |  |
| **Age at diagnosis (years)** |  |  |  |  |  |  |  |
| Mean (SD) | 65.7 (7.3) |  | 77.8 (10.2) |  | 55.0 (11.5) |  | 66.5 (13.3) |
| Median (IQR) | 67.0 (62.0, 71.0) |  | 79.0 (71.0, 86.0) |  | 55.0 (47.0, 64.0) |  | 67.0 (57.0, 76.0) |
| **Age group at diagnosis (years), *n (%)*** |  |  |  |  |  |  |  |
| 18 to 39 | 0 (0.0) |  | 0 (0.0) |  | 1,051 (9.9) |  | 352 (1.8) |
| 40 to 59 | 41 (20.7) |  | 73 (4.4) |  | 5,448 (51.3) |  | 5,441 (27.1) |
| 60 to 79 | 153 (77.3) |  | 773 (46.5) |  | 4,095 (38.5) |  | 10,608 (52.8) |
| 80 plus | 4 (2.0) |  | 815 (49.1) |  | 31 (0.3) |  | 3,705 (18.4) |
| **Year of breast cancer diagnosis, *n (%)*** |  |  |  |  |  |  |  |
| 2010 to 2011 | 43 (21.7) |  | 467 (28.1) |  | 2,469 (23.2) |  | 5,932 (29.5) |
| 2012 to 2013 | 68 (34.3) |  | 523 (31.5) |  | 3,800 (35.8) |  | 6,228 (31.0) |
| 2014 to 2015 | 87 (43.9) |  | 671 (40.4) |  | 4,356 (41.0) |  | 7,946 (39.5) |
| **Region of Sweden, *n (%)*** |  |  |  |  |  |  |  |
| North | 20 (10.1) |  | 151 (9.1) |  | 895 (8.4) |  | 1,423 (7.1) |
| South | 43 (21.7) |  | 301 (18.1) |  | 2,002 (18.8) |  | 3,665 (18.2) |
| Southeast | 30 (15.2) |  | 160 (9.6) |  | 1,099 (10.3) |  | 1,904 (9.5) |
| Stockholm/Gotland | 43 (21.7) |  | 344 (20.7) |  | 2,816 (26.5) |  | 3,938 (19.6) |
| Uppsala/Örebro | 33 (16.7) |  | 383 (23.1) |  | 1,882 (17.7) |  | 4,628 (23.0) |
| West | 29 (14.6) |  | 322 (19.4) |  | 1,931 (18.2) |  | 4,548 (22.6) |
| **Civil status, *n (%)*** |  |  |  |  |  |  |  |
| Unmarried | 13 (6.6) |  | 106 (6.4) |  | 2,367 (22.3) |  | 2,817 (14.0) |
| Married | 116 (58.6) |  | 605 (36.4) |  | 5,840 (55.0) |  | 9,835 (48.9) |
| Divorced/separated | 50 (25.3) |  | 289 (17.4) |  | 1,877 (17.7) |  | 3,703 (18.4) |
| Widow | 19 (9.6) |  | 661 (39.8) |  | 538 (5.1) |  | 3,748 (18.6) |
| Unknown | 13 (6.6) |  | 106 (6.4) |  | 3 (0.0) |  | 3 (0.0) |
| **Level of education, *n (%)*** |  |  |  |  |  |  |  |
| Compulsory education or less | 53 (26.8) |  | 743 (44.7) |  | 1,529 (14.4) |  | 5,573 (27.7) |
| Upper secondary | 95 (48.0) |  | 631 (38.0) |  | 4,588 (43.2) |  | 8,080 (40.2) |
| College/ University/ Research | 50 (25.3) |  | 256 (15.4) |  | 4,452 (41.9) |  | 6,256 (31.1) |
| Unknown | 0 (0.0) |  | 31 (1.9) |  | 56 (0.5) |  | 197 (1.0) |
| **Disposable income SEK, *n (%)*** |  |  |  |  |  |  |  |
| 1 to 1,000 | 21 (10.6) |  | 270 (16.3) |  | 795 (7.5) |  | 2,280 (11.3) |
| 1,001 to 2,000 | 114 (57.6) |  | 1,148 (69.1) |  | 3,575 (33.6) |  | 10,147 (50.5) |
| 2,001 to 3,000 | 41 (20.7) |  | 160 (9.6) |  | 3,898 (36.7) |  | 4,803 (23.9) |
| > 3,000 | 21 (10.6) |  | 79 (4.8) |  | 2,241 (21.1) |  | 2,755 (13.7) |
| Unknown | 1 (0.5) |  | 4 (0.2) |  | 116 (1.1) |  | 121 (0.6) |
| **Tumour characteristics, *n (%)*** | | | | |  |  |  |
| **TNM stage** |  |  |  |  |  |  |  |
| Stage 1 | 83 (41.9) |  | 771 (46.4) |  | 4,369 (41.1) |  | 12,742 (63.4) |
| Stage 2 | 105 (53.0) |  | 808 (48.6) |  | 5,602 (52.7) |  | 6,747 (33.6) |
| Stage 3 | 10 (5.1) |  | 82 (4.9) |  | 654 (6.2) |  | 617 (3.1) |
| **Histological grade** |  |  |  |  |  |  |  |
| Grade 1 | 6 (3.0) |  | 260 (15.7) |  | 406 (3.8) |  | 4,569 (22.7) |
| Grade 2 | 67 (33.8) |  | 662 (39.9) |  | 3,451 (32.5) |  | 9,847 (49.0) |
| Grade 3 | 99 (50.0) |  | 311 (18.7) |  | 4,998 (47.0) |  | 3,350 (16.7) |
| Unknown | 26 (13.1) |  | 428 (25.8) |  | 1,770 (16.7) |  | 2,340 (11.6) |
| **HER2 status** |  |  |  |  |  |  |  |
| Negative | 138 (69.7) |  | 1,220 (73.4) |  | 7,594 (71.5) |  | 16,200 (80.6) |
| Positive | 51 (25.8) |  | 122 (7.3) |  | 2,666 (25.1) |  | 1,127 (5.6) |
| Unknown/ unrecorded | 9 (4.5) |  | 319 (19.2) |  | 365 (3.4) |  | 2,779 (13.8) |
| **ER status** |  |  |  |  |  |  |  |
| Negative | 126 (63.6) |  | 1,271 (76.5) |  | 7,708 (72.5) |  | 16,913 (84.1) |
| Positive | 70 (35.4) |  | 168 (10.1) |  | 2,789 (26.2) |  | 1,296 (6.4) |
| Unknown | 2 (1.0) |  | 222 (13.4) |  | 128 (1.2) |  | 1,897 (9.4) |
| **PR status** |  |  |  |  |  |  |  |
| Negative | 94 (47.5) |  | 1,081 (65.1) |  | 6,274 (59.0) |  | 14,573 (72.5) |
| Positive | 101 (51.0) |  | 355 (21.4) |  | 4,208 (39.6) |  | 3,610 (18.0) |
| Unknown | 3 (1.5) |  | 225 (13.5) |  | 143 (1.3) |  | 1,923 (9.6) |
| **Menopausal status** |  |  |  |  |  |  |  |
| Pre-menopausal | 7 (3.5) |  | 15 (0.9) |  | 3,761 (35.4) |  | 2,806 (14.0) |
| Post-menopausal | 173 (87.4) |  | 1,603 (96.5) |  | 5,896 (55.5) |  | 15,904 (79.1) |
| Uncertain (e.g., hysterectomy) | 11 (5.6) |  | 22 (1.3) |  | 535 (5.0) |  | 754 (3.8) |
| Unknown | 7 (3.5) |  | 21 (1.3) |  | 433 (4.1) |  | 642 (3.2) |
| **Comorbidities at breast cancer diagnosis, *n (%)*** |  |  |  |  |  |  |  |
| Diabetes | 41 (20.7) |  | 400 (24.1) |  | 506 (4.8) |  | 1,553 (7.7) |
| CKD | 1 (0.5) |  | 63 (3.8) |  | 18 (0.2) |  | 148 (0.7) |
| COPD | 8 (4.0) |  | 175 (10.5) |  | 128 (1.2) |  | 629 (3.1) |
| **Treatment** |  |  |  |  |  |  |  |
| **Surgery** |  |  |  |  |  |  |  |
| Primary operation | 172 (86.9) |  | 1,265 (76.2) |  | 8,893 (83.7) |  | 18,415 (91.6) |
| Pre-op oncological or conservative treatment | 26 (13.1) |  | 259 (15.6) |  | 1,718 (16.2) |  | 1,084 (5.4) |
| No surgery | 0 (0.0) |  | 137 (8.2) |  | 14 (0.1) |  | 602 (3.0) |
| Unknown | 0 (0.0) |  | 0 (0.0) |  | 0 (0.0) |  | 5 (0.0) |
| **Chemotherapy** |  |  |  |  |  |  |  |
| Anthracyclines | 198 (100.0) |  | 0 (0.0) |  | 10,625 (100.0) |  | 0 (0.0) |
| Docetaxel | 43 (21.7) |  | 4 (0.2) |  | 2,559 (24.1) |  | 86 (0.4) |
| Paclitaxel | 19 (9.6) |  | 10 (0.6) |  | 875 (8.2) |  | 149 (0.7) |
| Other chemotherapy | 6 (3.0) |  | 17 (1.0) |  | 417 (3.9) |  | 196 (1.0) |
| **Antibody therapy** | | | | | | | |
| Trastuzumab | 48 (24.2) |  | 23 (1.4) |  | 2,730 (25.7) |  | 348 (1.7) |
| Pertuzumab | 0 (0.0) |  | 0 (0.0) |  | 7 (0.1) |  | 0 (0.0) |
| **Endocrine therapy** | | | | | | | |
| Outpatient tamoxifen | 16 (8.1) |  | 359 (21.6) |  | 3,486 (32.8) |  | 8,092 (40.2) |
| Outpatient GNRH | 1 (0.5) |  | 5 (0.3) |  | 506 (4.8) |  | 175 (0.9) |
| Outpatient AI | 111 (56.1) |  | 1,041 (62.7) |  | 4,684 (44.1) |  | 8,848 (44.0) |
| **Radiotherapy** | | | | | | | |
| Not recorded | 43 (21.7) |  | 1,132 (68.2) |  | 1,769 (16.6) |  | 8,987 (44.7) |
| Right breast | 84 (42.4) |  | 253 (15.2) |  | 4,387 (41.3) |  | 5,445 (27.1) |
| Left breast | 71 (35.9) |  | 276 (16.6) |  | 4,469 (42.1) |  | 5,674 (28.2) |

AI = aromatase inhibitors; COPD = chronic obstructive pulmonary disease; CKD = chronic kidney disease; CAD = coronary artery disease; ER = oestrogen receptor; GNRH = Gonadotropin-releasing hormone; HER2 = Human Epidermal Growth Factor Receptor 2; IQR = interquartile range; n/a = not applicable; PR = progesterone receptor; SD = standard deviation; TNM = UICC TNM Classification of Malignant Tumours; VTE = venous thromboembolism.

## Supplementary Table 4 Patient and tumour characteristics of patients with and without prior heart failure (HF) that received/did not receive anthracyclines.

|  | **Breast cancer patients with a history of HF at diagnosis** (N=1,152) | | |  | **Breast cancer patients with no history of HF at diagnosis** (N=31,438) | | |
| --- | --- | --- | --- | --- | --- | --- | --- |
|  | **Anthracyclines**  (N=42, 3.6%) |  | **No anthracyclines**  (N=1,110, 96.4%) |  | **Anthracyclines**  (N=10,781, 34.3%) |  | **No anthracyclines**  (N=20,657, 65.7%) |
| **Type of cardiovascular disease, *n (%)*** | | | | |  |  |  |
| CAD | 18 (42.9) |  | 455 (41.0) |  | 180 (1.7) |  | 1,206 (5.8) |
| Hypertension | 26 (61.9) |  | 773 (69.6) |  | 1,033 (9.6) |  | 4,624 (22.4) |
| Stroke | 2 (4.8) |  | 154 (13.9) |  | 129 (1.2) |  | 855 (4.1) |
| VTE | 1 (2.4) |  | 105 (9.5) |  | 135 (1.3) |  | 527 (2.6) |
| **Demographics** |  |  |  |  |  |  |  |
| **Age at diagnosis (years)** |  |  |  |  |  |  |  |
| Mean (SD) | 64.5 (9.7) |  | 80.4 (9.8) |  | 55.2 (11.6) |  | 66.7 (13.2) |
| Median (IQR) | 68.0 (61.0, 71.0) |  | 82.0 (74.0, 87.0) |  | 56.0 (47.0, 64.0) |  | 67.0 (58.0, 76.0) |
| **Age group at diagnosis (years), *n (%)*** |  |  |  |  |  |  |  |
| 18 to 39 | 1 (2.4) |  | 1 (0.1) |  | 1,050 (9.7) |  | 351 (1.7) |
| 40 to 59 | 9 (21.4) |  | 33 (3.0) |  | 5,480 (50.8) |  | 5,481 (26.5) |
| 60 to 79 | 31 (73.8) |  | 400 (36.0) |  | 4,217 (39.1) |  | 10,981 (53.2) |
| 80 plus | 1 (2.4) |  | 676 (60.9) |  | 34 (0.3) |  | 3,844 (18.6) |
| **Year of breast cancer diagnosis, *n (%)*** |  |  |  |  |  |  |  |
| 2010 to 2011 | 6 (14.3) |  | 302 (27.2) |  | 2,506 (23.2) |  | 6,097 (29.5) |
| 2012 to 2013 | 18 (42.9) |  | 371 (33.4) |  | 3,850 (35.7) |  | 6,380 (30.9) |
| 2014 to 2015 | 18 (42.9) |  | 437 (39.4) |  | 4,425 (41.0) |  | 8,180 (39.6) |
| **Region of Sweden, *n (%)*** |  |  |  |  |  |  |  |
| North | 4 (9.5) |  | 83 (7.5) |  | 911 (8.5) |  | 1,491 (7.2) |
| South | 6 (14.3) |  | 216 (19.5) |  | 2,039 (18.9) |  | 3,750 (18.2) |
| Southeast | 6 (14.3) |  | 96 (8.6) |  | 1,123 (10.4) |  | 1,968 (9.5) |
| Stockholm/Gotland | 8 (19.0) |  | 263 (23.7) |  | 2,851 (26.4) |  | 4,019 (19.5) |
| Uppsala/Örebro | 8 (19.0) |  | 254 (22.9) |  | 1,907 (17.7) |  | 4,757 (23.0) |
| West | 10 (23.8) |  | 198 (17.8) |  | 1,950 (18.1) |  | 4,672 (22.6) |
| **Civil status, *n (%)*** |  |  |  |  |  |  |  |
| Unmarried | 6 (14.3) |  | 106 (9.5) |  | 2,374 (22.0) |  | 2,817 (13.6) |
| Married | 18 (42.9) |  | 299 (26.9) |  | 5,938 (55.1) |  | 10,141 (49.1) |
| Divorced/separated | 15 (35.7) |  | 194 (17.5) |  | 1,912 (17.7) |  | 3,798 (18.4) |
| Widow | 3 (7.1) |  | 511 (46.0) |  | 554 (5.1) |  | 3,898 (18.9) |
| Unknown | 6 (14.3) |  | 106 (9.5) |  | 3 (0.0) |  | 3 (0.0) |
| **Level of education, *n (%)*** |  |  |  |  |  |  |  |
| Compulsory education or less | 13 (31.0) |  | 555 (50.0) |  | 1,569 (14.6) |  | 5,761 (27.9) |
| Upper secondary | 20 (47.6) |  | 371 (33.4) |  | 4,663 (43.3) |  | 8,340 (40.4) |
| College/ University/ Research | 9 (21.4) |  | 154 (13.9) |  | 4,493 (41.7) |  | 6,358 (30.8) |
| Unknown | 0 (0.0) |  | 30 (2.7) |  | 56 (0.5) |  | 198 (1.0) |
| **Disposable income SEK, *n (%)*** |  |  |  |  |  |  |  |
| 1 to 1,000 | 5 (11.9) |  | 177 (15.9) |  | 811 (7.5) |  | 2,373 (11.5) |
| 1,001 to 2,000 | 28 (66.7) |  | 801 (72.2) |  | 3,661 (34.0) |  | 10,494 (50.8) |
| 2,001 to 3,000 | 6 (14.3) |  | 86 (7.7) |  | 3,933 (36.5) |  | 4,877 (23.6) |
| > 3,000 | 3 (7.1) |  | 44 (4.0) |  | 2,259 (21.0) |  | 2,790 (13.5) |
| Unknown | 0 (0.0) |  | 2 (0.2) |  | 117 (1.1) |  | 123 (0.6) |
| **Tumour characteristics, *n (%)*** | | | | |  |  |  |
| **TNM stage** |  |  |  |  |  |  |  |
| Stage 1 | 14 (33.3) |  | 430 (38.7) |  | 4,438 (41.2) |  | 13,083 (63.3) |
| Stage 2 | 26 (61.9) |  | 603 (54.3) |  | 5,681 (52.7) |  | 6,952 (33.7) |
| Stage 3 | 2 (4.8) |  | 77 (6.9) |  | 662 (6.1) |  | 622 (3.0) |
| **Histological grade** |  |  |  |  |  |  |  |
| Grade 1 | 1 (2.4) |  | 128 (11.5) |  | 411 (3.8) |  | 4,701 (22.8) |
| Grade 2 | 12 (28.6) |  | 340 (30.6) |  | 3,506 (32.5) |  | 10,169 (49.2) |
| Grade 3 | 23 (54.8) |  | 208 (18.7) |  | 5,074 (47.1) |  | 3,453 (16.7) |
| Unknown | 6 (14.3) |  | 434 (39.1) |  | 1,790 (16.6) |  | 2,334 (11.3) |
| **HER2 status** |  |  |  |  |  |  |  |
| Negative | 24 (57.1) |  | 739 (66.6) |  | 7,708 (71.5) |  | 16,681 (80.8) |
| Positive | 14 (33.3) |  | 71 (6.4) |  | 2,703 (25.1) |  | 1,178 (5.7) |
| Unknown/ unrecorded | 4 (9.5) |  | 300 (27.0) |  | 370 (3.4) |  | 2,798 (13.5) |
| **ER status** |  |  |  |  |  |  |  |
| Negative | 26 (61.9) |  | 785 (70.7) |  | 7,808 (72.4) |  | 17,399 (84.2) |
| Positive | 15 (35.7) |  | 107 (9.6) |  | 2,844 (26.4) |  | 1,357 (6.6) |
| Unknown | 1 (2.4) |  | 218 (19.6) |  | 129 (1.2) |  | 1,901 (9.2) |
| **PR status** |  |  |  |  |  |  |  |
| Negative | 19 (45.2) |  | 670 (60.4) |  | 6,349 (58.9) |  | 14,984 (72.5) |
| Positive | 21 (50.0) |  | 222 (20.0) |  | 4,288 (39.8) |  | 3,743 (18.1) |
| Unknown | 2 (4.8) |  | 218 (19.6) |  | 144 (1.3) |  | 1,930 (9.3) |
| **Menopausal status** |  |  |  |  |  |  |  |
| Pre-menopausal | 3 (7.1) |  | 10 (0.9) |  | 3,765 (34.9) |  | 2,811 (13.6) |
| Post-menopausal | 33 (78.6) |  | 1,079 (97.2) |  | 6,036 (56.0) |  | 16,428 (79.5) |
| Uncertain (e.g., hysterectomy) | 3 (7.1) |  | 8 (0.7) |  | 543 (5.0) |  | 768 (3.7) |
| Unknown | 3 (7.1) |  | 13 (1.2) |  | 437 (4.1) |  | 650 (3.1) |
| **Comorbidities at breast cancer diagnosis, *n (%)*** |  |  |  |  |  |  |  |
| Diabetes | 8 (19.0) |  | 298 (26.8) |  | 539 (5.0) |  | 1,655 (8.0) |
| CKD | 1 (2.4) |  | 85 (7.7) |  | 18 (0.2) |  | 126 (0.6) |
| COPD | 4 (9.5) |  | 161 (14.5) |  | 132 (1.2) |  | 643 (3.1) |
| **Breast cancer treatment in the year after diagnosis, *n (%)*** |  |  |  |  |  |  |  |
| **Surgery** |  |  |  |  |  |  |  |
| Primary operation | 36 (85.7) |  | 694 (62.5) |  | 9,029 (83.7) |  | 18,986 (91.9) |
| Pre-op oncological or conservative treatment | 6 (14.3) |  | 262 (23.6) |  | 1,738 (16.1) |  | 1,081 (5.2) |
| No surgery | 0 (0.0) |  | 154 (13.9) |  | 14 (0.1) |  | 585 (2.8) |
| Unknown | 36 (85.7) |  | 694 (62.5) |  | 0 (0.0) |  | 5 (0.0) |
| **Chemotherapy** |  |  |  |  |  |  |  |
| Anthracyclines | 42 (100.0) |  | 0 (0.0) |  | 10,781 (100.0) |  | 0 (0.0) |
| Docetaxel | 6 (14.3) |  | 5 (0.5) |  | 2,596 (24.1) |  | 85 (0.4) |
| Paclitaxel | 3 (7.1) |  | 5 (0.5) |  | 891 (8.3) |  | 154 (0.7) |
| Other chemotherapy | 1 (2.4) |  | 12 (1.1) |  | 422 (3.9) |  | 201 (1.0) |
| **Antibody therapy** | | | | | | | |
| Trastuzumab | 14 (33.3) |  | 10 (0.9) |  | 2,764 (25.6) |  | 361 (1.7) |
| Pertuzumab | 0 (0.0) |  | 0 (0.0) |  | 7 (0.1) |  | 0 (0.0) |
| **Endocrine therapy** | | | | | | | |
| Outpatient tamoxifen | 4 (9.5) |  | 214 (19.3) |  | 3,498 (32.4) |  | 8,237 (39.9) |
| Outpatient GNRH | 2 (4.8) |  | 1 (0.1) |  | 505 (4.7) |  | 179 (0.9) |
| Outpatient AI | 24 (57.1) |  | 748 (67.4) |  | 4,771 (44.3) |  | 9,141 (44.3) |
| **Radiotherapy** | | | | | | | |
| Not recorded | 3 (7.1) |  | 861 (77.6) |  | 1,809 (16.8) |  | 9,258 (44.8) |
| Right breast | 22 (52.4) |  | 132 (11.9) |  | 4,449 (41.3) |  | 5,566 (26.9) |
| Left breast | 17 (40.5) |  | 117 (10.5) |  | 4,523 (42.0) |  | 5,833 (28.2) |

AI = aromatase inhibitors; COPD = chronic obstructive pulmonary disease; CKD = chronic kidney disease; CAD = coronary artery disease; ER = oestrogen receptor; GNRH = Gonadotropin-releasing hormone; HER2 = Human Epidermal Growth Factor Receptor 2; IQR = interquartile range; n/a = not applicable; PR = progesterone receptor; SD = standard deviation; TNM = UICC TNM Classification of Malignant Tumours; VTE = venous thromboembolism.

## Supplementary Table 5 Patient and tumour characteristics of patients with and without prior hypertension that received/did not receive anthracyclines.

|  | **Breast cancer patients with hypertension at diagnosis** (N=6,456) | | |  | **Breast cancer patients with no hypertension at diagnosis** (N=26,134) | | |
| --- | --- | --- | --- | --- | --- | --- | --- |
|  | **Anthracyclines**  (N=1,059, 16.4%) |  | **No anthracyclines**  (N=5,397, 83.6%) |  | **Anthracyclines**  (N=9,764, 37.4%) |  | **No anthracyclines**  (N=16,370, 62.6%) |
| **Type of cardiovascular disease, *n (%)*** | | | | |  |  |  |
| CAD | 111 (10.5) |  | 1,126 (20.9) |  | 87 (0.9) |  | 535 (3.3) |
| Heart failure | 26 (2.5) |  | 773 (14.3) |  | 16 (0.2) |  | 337 (2.1) |
| Hypertension | 1,059 (100.0) |  | 5,397 (100.0) |  | 0 (0.0) |  | 0 (0.0) |
| Stroke | 67 (6.3) |  | 703 (13.0) |  | 64 (0.7) |  | 306 (1.9) |
| VTE | 38 (3.6) |  | 313 (5.8) |  | 98 (1.0) |  | 319 (1.9) |
| **Demographics** |  |  |  |  |  |  |  |
| **Age at diagnosis (years)** |  |  |  |  |  |  |  |
| Mean (SD) | 64.4 (8.4) |  | 75.8 (10.3) |  | 54.2 (11.4) |  | 64.6 (13.1) |
| Median (IQR) | 66.0 (60.0, 70.0) |  | 77.0 (69.0, 84.0) |  | 54.0 (46.0, 63.0) |  | 65.0 (55.0, 73.0) |
| **Age group at diagnosis (years), *n (%)*** |  |  |  |  |  |  |  |
| 18 to 39 | 10 (0.9) |  | 3 (0.1) |  | 1,041 (10.7) |  | 349 (2.1) |
| 40 to 59 | 250 (23.6) |  | 323 (6.0) |  | 5,239 (53.7) |  | 5,191 (31.7) |
| 60 to 79 | 783 (73.9) |  | 2,884 (53.4) |  | 3,465 (35.5) |  | 8,497 (51.9) |
| 80 plus | 16 (1.5) |  | 2,187 (40.5) |  | 19 (0.2) |  | 2,333 (14.3) |
| **Year of breast cancer diagnosis, *n (%)*** |  |  |  |  |  |  |  |
| 2010 to 2011 | 203 (19.2) |  | 1,321 (24.5) |  | 2,309 (23.6) |  | 5,078 (31.0) |
| 2012 to 2013 | 372 (35.1) |  | 1,756 (32.5) |  | 3,496 (35.8) |  | 4,995 (30.5) |
| 2014 to 2015 | 484 (45.7) |  | 2,320 (43.0) |  | 3,959 (40.5) |  | 6,297 (38.5) |
| **Region of Sweden, *n (%)*** |  |  |  |  |  |  |  |
| North | 129 (12.2) |  | 430 (8.0) |  | 786 (8.0) |  | 1,144 (7.0) |
| South | 185 (17.5) |  | 996 (18.5) |  | 1,860 (19.0) |  | 2,970 (18.1) |
| Southeast | 119 (11.2) |  | 536 (9.9) |  | 1,010 (10.3) |  | 1,528 (9.3) |
| Stockholm/Gotland | 257 (24.3) |  | 1,062 (19.7) |  | 2,602 (26.6) |  | 3,220 (19.7) |
| Uppsala/Örebro | 204 (19.3) |  | 1,186 (22.0) |  | 1,711 (17.5) |  | 3,825 (23.4) |
| West | 165 (15.6) |  | 1,187 (22.0) |  | 1,795 (18.4) |  | 3,683 (22.5) |
| **Civil status, *n (%)*** |  |  |  |  |  |  |  |
| Unmarried | 134 (12.7) |  | 431 (8.0) |  | 2,246 (23.0) |  | 2,492 (15.2) |
| Married | 601 (56.8) |  | 2,179 (40.4) |  | 5,355 (54.8) |  | 8,261 (50.5) |
| Divorced/separated | 205 (19.4) |  | 913 (16.9) |  | 1,722 (17.6) |  | 3,079 (18.8) |
| Widow | 119 (11.2) |  | 1,874 (34.7) |  | 438 (4.5) |  | 2,535 (15.5) |
| Unknown | 134 (12.7) |  | 431 (8.0) |  | 3 (0.0) |  | 3 (0.0) |
| **Level of education, *n (%)*** |  |  |  |  |  |  |  |
| Compulsory education or less | 233 (22.0) |  | 2,263 (41.9) |  | 1,349 (13.8) |  | 4,053 (24.8) |
| Upper secondary | 501 (47.3) |  | 2,013 (37.3) |  | 4,182 (42.8) |  | 6,698 (40.9) |
| College/ University/ Research | 317 (29.9) |  | 1,051 (19.5) |  | 4,185 (42.9) |  | 5,461 (33.4) |
| Unknown | 8 (0.8) |  | 70 (1.3) |  | 48 (0.5) |  | 158 (1.0) |
| **Disposable income SEK, *N (%)*** |  |  |  |  |  |  |  |
| 1 to 1,000 | 121 (11.4) |  | 820 (15.2) |  | 695 (7.1) |  | 1,730 (10.6) |
| 1,001 to 2,000 | 541 (51.1) |  | 3,593 (66.6) |  | 3,148 (32.2) |  | 7,702 (47.0) |
| 2,001 to 3,000 | 253 (23.9) |  | 619 (11.5) |  | 3,686 (37.8) |  | 4,344 (26.5) |
| > 3,000 | 140 (13.2) |  | 357 (6.6) |  | 2,122 (21.7) |  | 2,477 (15.1) |
| Unknown | 4 (0.4) |  | 8 (0.1) |  | 113 (1.2) |  | 117 (0.7) |
| **Tumour characteristics, *n (%)*** | | | | |  |  |  |
| **TNM stage** |  |  |  |  |  |  |  |
| Stage 1 | 386 (36.4) |  | 2,744 (50.8) |  | 4,066 (41.6) |  | 10,769 (65.8) |
| Stage 2 | 607 (57.3) |  | 2,397 (44.4) |  | 5,100 (52.2) |  | 5,158 (31.5) |
| Stage 3 | 66 (6.2) |  | 256 (4.7) |  | 598 (6.1) |  | 443 (2.7) |
| **Histological grade** |  |  |  |  |  |  |  |
| Grade 1 | 34 (3.2) |  | 887 (16.4) |  | 378 (3.9) |  | 3,942 (24.1) |
| Grade 2 | 343 (32.4) |  | 2,332 (43.2) |  | 3,175 (32.5) |  | 8,177 (50.0) |
| Grade 3 | 545 (51.5) |  | 1,066 (19.8) |  | 4,552 (46.6) |  | 2,595 (15.9) |
| Unknown | 137 (12.9) |  | 1,112 (20.6) |  | 1,659 (17.0) |  | 1,656 (10.1) |
| **HER2 status** |  |  |  |  |  |  |  |
| Negative | 777 (73.4) |  | 4,115 (76.2) |  | 6,955 (71.2) |  | 13,305 (81.3) |
| Positive | 254 (24.0) |  | 374 (6.9) |  | 2,463 (25.2) |  | 875 (5.3) |
| Unknown/ unrecorded | 28 (2.6) |  | 908 (16.8) |  | 346 (3.5) |  | 2,190 (13.4) |
| **ER status** |  |  |  |  |  |  |  |
| Negative | 752 (71.0) |  | 4,302 (79.7) |  | 7,082 (72.5) |  | 13,882 (84.8) |
| Positive | 302 (28.5) |  | 466 (8.6) |  | 2,557 (26.2) |  | 998 (6.1) |
| Unknown | 5 (0.5) |  | 629 (11.7) |  | 125 (1.3) |  | 1,490 (9.1) |
| **PR status** |  |  |  |  |  |  |  |
| Negative | 601 (56.8) |  | 3,599 (66.7) |  | 5,767 (59.1) |  | 12,055 (73.6) |
| Positive | 451 (42.6) |  | 1,164 (21.6) |  | 3,858 (39.5) |  | 2,801 (17.1) |
| Unknown | 7 (0.7) |  | 634 (11.7) |  | 139 (1.4) |  | 1,514 (9.2) |
| **Menopausal status** |  |  |  |  |  |  |  |
| Pre-menopausal | 79 (7.5) |  | 85 (1.6) |  | 3,689 (37.8) |  | 2,736 (16.7) |
| Post-menopausal | 888 (83.9) |  | 5,115 (94.8) |  | 5,181 (53.1) |  | 12,392 (75.7) |
| Uncertain (e.g., hysterectomy) | 54 (5.1) |  | 107 (2.0) |  | 492 (5.0) |  | 669 (4.1) |
| Unknown | 38 (3.6) |  | 90 (1.7) |  | 402 (4.1) |  | 573 (3.5) |
| **Comorbidities at breast cancer diagnosis, *n (%)*** |  |  |  |  |  |  |  |
| Diabetes | 220 (20.8) |  | 1,133 (21.0) |  | 327 (3.3) |  | 820 (5.0) |
| CKD | 11 (1.0) |  | 169 (3.1) |  | 8 (0.1) |  | 42 (0.3) |
| COPD | 48 (4.5) |  | 425 (7.9) |  | 88 (0.9) |  | 379 (2.3) |
| **Breast cancer treatment in the year after diagnosis, *n (%)*** |  |  |  |  |  |  |  |
| **Surgery** |  |  |  |  |  |  |  |
| Primary operation | 923 (87.2) |  | 4,411 (81.7) |  | 8,142 (83.4) |  | 15,269 (93.3) |
| Pre-op oncological or conservative treatment | 136 (12.8) |  | 636 (11.8) |  | 1,608 (16.5) |  | 707 (4.3) |
| No surgery | 0 (0.0) |  | 348 (6.4) |  | 14 (0.1) |  | 391 (2.4) |
| Unknown | 0 (0.0) |  | 2 (0.0) |  | 0 (0.0) |  | 3 (0.0) |
| **Chemotherapy** |  |  |  |  |  |  |  |
| Anthracyclines | 1,059 (100.0) |  | 0 (0.0) |  | 9,764 (100.0) |  | 0 (0.0) |
| Docetaxel | 231 (21.8) |  | 16 (0.3) |  | 2,371 (24.3) |  | 74 (0.5) |
| Paclitaxel | 119 (11.2) |  | 33 (0.6) |  | 775 (7.9) |  | 126 (0.8) |
| Other chemotherapy | 40 (3.8) |  | 47 (0.9) |  | 383 (3.9) |  | 166 (1.0) |
| **Antibody therapy** | | | | | | | |
| Trastuzumab | 251 (23.7) |  | 72 (1.3) |  | 2,527 (25.9) |  | 299 (1.8) |
| Pertuzumab | 1 (0.1) |  | 0 (0.0) |  | 6 (0.1) |  | 0 (0.0) |
| **Endocrine therapy** | | | | | | | |
| Outpatient tamoxifen | 130 (12.3) |  | 1,477 (27.4) |  | 3,372 (34.5) |  | 6,974 (42.6) |
| Outpatient GNRH | 12 (1.1) |  | 9 (0.2) |  | 495 (5.1) |  | 171 (1.0) |
| Outpatient AI | 656 (61.9) |  | 3,213 (59.5) |  | 4,139 (42.4) |  | 6,676 (40.8) |
| **Radiotherapy** | | | | | | | |
| Not recorded | 187 (17.7) |  | 3,188 (59.1) |  | 1,625 (16.6) |  | 6,931 (42.3) |
| Right breast | 418 (39.5) |  | 1,080 (20.0) |  | 4,053 (41.5) |  | 4,618 (28.2) |
| Left breast | 454 (42.9) |  | 1,129 (20.9) |  | 4,086 (41.8) |  | 4,821 (29.5) |

AI = aromatase inhibitors; COPD = chronic obstructive pulmonary disease; CKD = chronic kidney disease; CAD = coronary artery disease; ER = oestrogen receptor; GNRH = Gonadotropin-releasing hormone; HER2 = Human Epidermal Growth Factor Receptor 2; IQR = interquartile range; n/a = not applicable; PR = progesterone receptor; SD = standard deviation; TNM = UICC TNM Classification of Malignant Tumours; VTE = venous thromboembolism.

## Supplementary Table 6 Patient and tumour characteristics of patients with and without prior stroke that received/did not receive anthracyclines.

|  | **Breast cancer patients with a history of stroke at diagnosis** (N=1,140) | | |  | **Breast cancer patients with no history of stroke at diagnosis** (N=31,450) | | |
| --- | --- | --- | --- | --- | --- | --- | --- |
|  | **Anthracyclines**  (N=131, 11.5%) |  | **No anthracyclines**  (N=1,009, 88.5%) |  | **Anthracyclines**  (N=10,692, 34.0%) |  | **No anthracyclines**  (N=20,758, 66.0%) |
| **Type of cardiovascular disease, *n (%)*** | | | | |  |  |  |
| CAD | 9 (6.9) |  | 181 (17.9) |  | 189 (1.8) |  | 1,480 (7.1) |
| Heart failure | 2 (1.5) |  | 154 (15.3) |  | 40 (0.4) |  | 956 (4.6) |
| Hypertension | 67 (51.1) |  | 703 (69.7) |  | 992 (9.3) |  | 4,694 (22.6) |
| Stroke | 131 (100.0) |  | 1,009 (100.0) |  | 0 (0.0) |  | 0 (0.0) |
| VTE | 4 (3.1) |  | 60 (5.9) |  | 132 (1.2) |  | 572 (2.8) |
| **Demographics** |  |  |  |  |  |  |  |
| **Age at diagnosis (years)** |  |  |  |  |  |  |  |
| Mean (SD) | 63.2 (8.6) |  | 77.9 (10.2) |  | 55.1 (11.6) |  | 66.9 (13.3) |
| Median (IQR) | 64.0 (58.0, 70.0) |  | 80.0 (71.0, 86.0) |  | 56.0 (47.0, 64.0) |  | 67.0 (58.0, 77.0) |
| **Age group at diagnosis (years), *n (%)*** |  |  |  |  |  |  |  |
| 18 to 39 | 2 (1.5) |  | 1 (0.1) |  | 1,049 (9.8) |  | 351 (1.7) |
| 40 to 59 | 36 (27.5) |  | 46 (4.6) |  | 5,453 (51.0) |  | 5,468 (26.3) |
| 60 to 79 | 92 (70.2) |  | 455 (45.1) |  | 4,156 (38.9) |  | 10,926 (52.6) |
| 80 plus | 1 (0.8) |  | 507 (50.2) |  | 34 (0.3) |  | 4,013 (19.3) |
| **Year of breast cancer diagnosis, *n (%)*** |  |  |  |  |  |  |  |
| 2010 to 2011 | 23 (17.6) |  | 271 (26.9) |  | 2,489 (23.3) |  | 6,128 (29.5) |
| 2012 to 2013 | 62 (47.3) |  | 333 (33.0) |  | 3,806 (35.6) |  | 6,418 (30.9) |
| 2014 to 2015 | 46 (35.1) |  | 405 (40.1) |  | 4,397 (41.1) |  | 8,212 (39.6) |
| **Region of Sweden, *n (%)*** |  |  |  |  |  |  |  |
| North | 16 (12.2) |  | 82 (8.1) |  | 899 (8.4) |  | 1,492 (7.2) |
| South | 29 (22.1) |  | 188 (18.6) |  | 2,016 (18.9) |  | 3,778 (18.2) |
| Southeast | 14 (10.7) |  | 110 (10.9) |  | 1,115 (10.4) |  | 1,954 (9.4) |
| Stockholm/Gotland | 26 (19.8) |  | 195 (19.3) |  | 2,833 (26.5) |  | 4,087 (19.7) |
| Uppsala/Örebro | 25 (19.1) |  | 211 (20.9) |  | 1,890 (17.7) |  | 4,800 (23.1) |
| West | 21 (16.0) |  | 223 (22.1) |  | 1,939 (18.1) |  | 4,647 (22.4) |
| **Civil status, *n (%)*** |  |  |  |  |  |  |  |
| Unmarried | 12 (9.2) |  | 69 (6.8) |  | 2,368 (22.1) |  | 2,854 (13.7) |
| Married | 67 (51.1) |  | 322 (31.9) |  | 5,889 (55.1) |  | 10,118 (48.7) |
| Divorced/separated | 39 (29.8) |  | 193 (19.1) |  | 1,888 (17.7) |  | 3,799 (18.3) |
| Widow | 13 (9.9) |  | 425 (42.1) |  | 544 (5.1) |  | 3,984 (19.2) |
| Unknown | 12 (9.2) |  | 69 (6.8) |  | 3 (0.0) |  | 3 (0.0) |
| **Level of education, *n (%)*** |  |  |  |  |  |  |  |
| Compulsory education or less | 33 (25.2) |  | 458 (45.4) |  | 1,549 (14.5) |  | 5,858 (28.2) |
| Upper secondary | 65 (49.6) |  | 368 (36.5) |  | 4,618 (43.2) |  | 8,343 (40.2) |
| College/ University/ Research | 32 (24.4) |  | 163 (16.2) |  | 4,470 (41.8) |  | 6,349 (30.6) |
| Unknown | 1 (0.8) |  | 20 (2.0) |  | 55 (0.5) |  | 208 (1.0) |
| **Disposable income SEK, *N (%)*** |  |  |  |  |  |  |  |
| 1 to 1,000 | 21 (16.0) |  | 164 (16.3) |  | 795 (7.4) |  | 2,386 (11.5) |
| 1,001 to 2,000 | 70 (53.4) |  | 706 (70.0) |  | 3,619 (33.8) |  | 10,589 (51.0) |
| 2,001 to 3,000 | 24 (18.3) |  | 83 (8.2) |  | 3,915 (36.6) |  | 4,880 (23.5) |
| > 3,000 | 16 (12.2) |  | 56 (5.6) |  | 2,246 (21.0) |  | 2,778 (13.4) |
| Unknown | 21 (16.0) |  | 164 (16.3) |  | 117 (1.1) |  | 125 (0.6) |
| **Tumour characteristics, *n (%)*** | | | | |  |  |  |
| **TNM stage** |  |  |  |  |  |  |  |
| Stage 1 | 50 (38.2) |  | 431 (42.7) |  | 4,402 (41.2) |  | 13,082 (63.0) |
| Stage 2 | 68 (51.9) |  | 514 (50.9) |  | 5,639 (52.7) |  | 7,041 (33.9) |
| Stage 3 | 13 (9.9) |  | 64 (6.3) |  | 651 (6.1) |  | 635 (3.1) |
| **Histological grade** |  |  |  |  |  |  |  |
| Grade 1 | 7 (5.3) |  | 125 (12.4) |  | 405 (3.8) |  | 4,704 (22.7) |
| Grade 2 | 42 (32.1) |  | 342 (33.9) |  | 3,476 (32.5) |  | 10,167 (49.0) |
| Grade 3 | 69 (52.7) |  | 208 (20.6) |  | 5,028 (47.0) |  | 3,453 (16.6) |
| Unknown | 13 (9.9) |  | 334 (33.1) |  | 1,783 (16.7) |  | 2,434 (11.7) |
| **HER2 status** |  |  |  |  |  |  |  |
| Negative | 96 (73.3) |  | 706 (70.0) |  | 7,636 (71.4) |  | 16,714 (80.5) |
| Positive | 28 (21.4) |  | 77 (7.6) |  | 2,689 (25.1) |  | 1,172 (5.6) |
| Unknown/ unrecorded | 7 (5.3) |  | 226 (22.4) |  | 367 (3.4) |  | 2,872 (13.8) |
| **ER status** |  |  |  |  |  |  |  |
| Negative | 92 (70.2) |  | 726 (72.0) |  | 7,742 (72.4) |  | 17,458 (84.1) |
| Positive | 38 (29.0) |  | 108 (10.7) |  | 2,821 (26.4) |  | 1,356 (6.5) |
| Unknown | 1 (0.8) |  | 175 (17.3) |  | 129 (1.2) |  | 1,944 (9.4) |
| **PR status** |  |  |  |  |  |  |  |
| Negative | 76 (58.0) |  | 604 (59.9) |  | 6,292 (58.8) |  | 15,050 (72.5) |
| Positive | 54 (41.2) |  | 228 (22.6) |  | 4,255 (39.8) |  | 3,737 (18.0) |
| Unknown | 1 (0.8) |  | 177 (17.5) |  | 145 (1.4) |  | 1,971 (9.5) |
| **Menopausal status** |  |  |  |  |  |  |  |
| Pre-menopausal | 14 (10.7) |  | 13 (1.3) |  | 3,754 (35.1) |  | 2,808 (13.5) |
| Post-menopausal | 106 (80.9) |  | 958 (94.9) |  | 5,963 (55.8) |  | 16,549 (79.7) |
| Uncertain (e.g., hysterectomy) | 8 (6.1) |  | 20 (2.0) |  | 538 (5.0) |  | 756 (3.6) |
| Unknown | 3 (2.3) |  | 18 (1.8) |  | 437 (4.1) |  | 645 (3.1) |
| **Comorbidities at breast cancer diagnosis, *n (%)*** |  |  |  |  |  |  |  |
| Diabetes | 24 (18.3) |  | 212 (21.0) |  | 523 (4.9) |  | 1,741 (8.4) |
| CKD | 2 (1.5) |  | 33 (3.3) |  | 17 (0.2) |  | 178 (0.9) |
| COPD | 5 (3.8) |  | 84 (8.3) |  | 131 (1.2) |  | 720 (3.5) |
| **Breast cancer treatment in the year after diagnosis, *n (%)*** |  |  |  |  |  |  |  |
| **Surgery** |  |  |  |  |  |  |  |
| Primary operation | 118 (90.1) |  | 701 (69.5) |  | 8,947 (83.7) |  | 18,979 (91.4) |
| Pre-op oncological or conservative treatment | 13 (9.9) |  | 186 (18.4) |  | 1,731 (16.2) |  | 1,157 (5.6) |
| No surgery | 0 (0.0) |  | 122 (12.1) |  | 14 (0.1) |  | 617 (3.0) |
| Unknown | 118 (90.1) |  | 701 (69.5) |  | 0 (0.0) |  | 5 (0.0) |
| **Chemotherapy** |  |  |  |  |  |  |  |
| Anthracyclines | 131 (100.0) |  | 0 (0.0) |  | 10,692 (100.0) |  | 0 (0.0) |
| Docetaxel | 21 (16.0) |  | 2 (0.2) |  | 2,581 (24.1) |  | 88 (0.4) |
| Paclitaxel | 9 (6.9) |  | 4 (0.4) |  | 885 (8.3) |  | 155 (0.7) |
| Other chemotherapy | 1 (0.8) |  | 8 (0.8) |  | 422 (3.9) |  | 205 (1.0) |
| **Antibody therapy** | | | | | | | |
| Trastuzumab | 30 (22.9) |  | 13 (1.3) |  | 2,748 (25.7) |  | 358 (1.7) |
| Pertuzumab | 0 (0.0) |  | 0 (0.0) |  | 7 (0.1) |  | 0 (0.0) |
| **Endocrine therapy** | | | | | | | |
| Outpatient tamoxifen | 12 (9.2) |  | 158 (15.7) |  | 3,490 (32.6) |  | 8,293 (40.0) |
| Outpatient GNRH | 2 (1.5) |  | 4 (0.4) |  | 505 (4.7) |  | 176 (0.8) |
| Outpatient AI | 76 (58.0) |  | 671 (66.5) |  | 4,719 (44.1) |  | 9,218 (44.4) |
| **Radiotherapy** | | | | | | | |
| Not recorded | 23 (17.6) |  | 698 (69.2) |  | 1,789 (16.7) |  | 9,421 (45.4) |
| Right breast | 53 (40.5) |  | 150 (14.9) |  | 4,418 (41.3) |  | 5,548 (26.7) |
| Left breast | 55 (42.0) |  | 161 (16.0) |  | 4,485 (41.9) |  | 5,789 (27.9) |

AI = aromatase inhibitors; COPD = chronic obstructive pulmonary disease; CKD = chronic kidney disease; CAD = coronary artery disease; ER = oestrogen receptor; GNRH = Gonadotropin-releasing hormone; HER2 = Human Epidermal Growth Factor Receptor 2; IQR = interquartile range; n/a = not applicable; PR = progesterone receptor; SD = standard deviation; TNM = UICC TNM Classification of Malignant Tumours; VTE = venous thromboembolism.

## Supplementary Table 7 Patient and tumour characteristics of patients with and without prior venous thromboembolism (VTE) that received/did not receive anthracyclines.

|  | **Breast cancer patients with a history of VTE at diagnosis** (N=768) | | |  | **Breast cancer patients with no history of VTE at diagnosis** (N=31,822) | | |
| --- | --- | --- | --- | --- | --- | --- | --- |
|  | **Anthracyclines**  (N=136, 17.7%) |  | **No anthracyclines**  (N=632, 82.3%) |  | **Anthracyclines**  (N=10,687, 33.6%) |  | **No anthracyclines**  (N=21,135, 66.4%) |
| **Type of cardiovascular disease, *n (%)*** | | | | |  |  |  |
| CAD | 5 (3.7) |  | 100 (15.8) |  | 193 (1.8) |  | 1,561 (7.4) |
| Heart failure | 1 (0.7) |  | 105 (16.6) |  | 41 (0.4) |  | 1,005 (4.8) |
| Hypertension | 38 (27.9) |  | 313 (49.5) |  | 1,021 (9.6) |  | 5,084 (24.1) |
| Stroke | 4 (2.9) |  | 60 (9.5) |  | 127 (1.2) |  | 949 (4.5) |
| VTE | 136 (100.0) |  | 632 (100.0) |  | 0 (0.0) |  | 0 (0.0) |
| **Demographics** |  |  |  |  |  |  |  |
| **Age at diagnosis (years)** |  |  |  |  |  |  |  |
| Mean (SD) | 60.8 (10.9) |  | 76.7 (11.1) |  | 55.1 (11.5) |  | 67.1 (13.4) |
| Median (IQR) | 62.5 (56.0, 69.0) |  | 79.0 (70.0, 85.0) |  | 56.0 (47.0, 64.0) |  | 68.0 (58.0, 77.0) |
| **Age group at diagnosis (years), *n (%)*** |  |  |  |  |  |  |  |
| 18 to 39 | 7 (5.1) |  | 1 (0.2) |  | 1,044 (9.8) |  | 351 (1.7) |
| 40 to 59 | 44 (32.4) |  | 45 (7.1) |  | 5,445 (50.9) |  | 5,469 (25.9) |
| 60 to 79 | 84 (61.8) |  | 287 (45.4) |  | 4,164 (39.0) |  | 11,094 (52.5) |
| 80 plus | 1 (0.7) |  | 299 (47.3) |  | 34 (0.3) |  | 4,221 (20.0) |
| **Year of breast cancer diagnosis, *n (%)*** |  |  |  |  |  |  |  |
| 2010 to 2011 | 33 (24.3) |  | 165 (26.1) |  | 2,479 (23.2) |  | 6,234 (29.5) |
| 2012 to 2013 | 37 (27.2) |  | 205 (32.4) |  | 3,831 (35.8) |  | 6,546 (31.0) |
| 2014 to 2015 | 66 (48.5) |  | 262 (41.5) |  | 4,377 (41.0) |  | 8,355 (39.5) |
| **Region of Sweden, *n (%)*** |  |  |  |  |  |  |  |
| North | 10 (7.4) |  | 43 (6.8) |  | 905 (8.5) |  | 1,531 (7.2) |
| South | 26 (19.1) |  | 117 (18.5) |  | 2,019 (18.9) |  | 3,849 (18.2) |
| Southeast | 22 (16.2) |  | 67 (10.6) |  | 1,107 (10.4) |  | 1,997 (9.4) |
| Stockholm/Gotland | 35 (25.7) |  | 125 (19.8) |  | 2,824 (26.4) |  | 4,157 (19.7) |
| Uppsala/Örebro | 22 (16.2) |  | 138 (21.8) |  | 1,893 (17.7) |  | 4,873 (23.1) |
| West | 21 (15.4) |  | 142 (22.5) |  | 1,939 (18.1) |  | 4,728 (22.4) |
| **Civil status, *n (%)*** |  |  |  |  |  |  |  |
| Unmarried | 17 (12.5) |  | 60 (9.5) |  | 2,363 (22.1) |  | 2,863 (13.5) |
| Married | 80 (58.8) |  | 227 (35.9) |  | 5,876 (55.0) |  | 10,213 (48.3) |
| Divorced/separated | 28 (20.6) |  | 101 (16.0) |  | 1,899 (17.8) |  | 3,891 (18.4) |
| Widow | 11 (8.1) |  | 244 (38.6) |  | 546 (5.1) |  | 4,165 (19.7) |
| Unknown | 17 (12.5) |  | 60 (9.5) |  | 3 (0.0) |  | 3 (0.0) |
| **Level of education, *n (%)*** |  |  |  |  |  |  |  |
| Compulsory education or less | 32 (23.5) |  | 304 (48.1) |  | 1,550 (14.5) |  | 6,012 (28.4) |
| Upper secondary | 69 (50.7) |  | 212 (33.5) |  | 4,614 (43.2) |  | 8,499 (40.2) |
| College/ University/ Research | 35 (25.7) |  | 114 (18.0) |  | 4,467 (41.8) |  | 6,398 (30.3) |
| Unknown | 0 (0.0) |  | 2 (0.3) |  | 56 (0.5) |  | 226 (1.1) |
| **Disposable income SEK, *N (%)*** |  |  |  |  |  |  |  |
| 1 to 1,000 | 16 (11.8) |  | 91 (14.4) |  | 800 (7.5) |  | 2,459 (11.6) |
| 1,001 to 2,000 | 63 (46.3) |  | 452 (71.5) |  | 3,626 (33.9) |  | 10,843 (51.3) |
| 2,001 to 3,000 | 42 (30.9) |  | 65 (10.3) |  | 3,897 (36.5) |  | 4,898 (23.2) |
| > 3,000 | 15 (11.0) |  | 22 (3.5) |  | 2,247 (21.0) |  | 2,812 (13.3) |
| Unknown | 0 (0.0) |  | 2 (0.3) |  | 117 (1.1) |  | 123 (0.6) |
| **Tumour characteristics, *n (%)*** | | | | |  |  |  |
| **TNM stage** |  |  |  |  |  |  |  |
| Stage 1 | 53 (39.0) |  | 304 (48.1) |  | 4,399 (41.2) |  | 13,209 (62.5) |
| Stage 2 | 77 (56.6) |  | 298 (47.2) |  | 5,630 (52.7) |  | 7,257 (34.3) |
| Stage 3 | 6 (4.4) |  | 30 (4.7) |  | 658 (6.2) |  | 669 (3.2) |
| **Histological grade** |  |  |  |  |  |  |  |
| Grade 1 | 4 (2.9) |  | 108 (17.1) |  | 408 (3.8) |  | 4,721 (22.3) |
| Grade 2 | 41 (30.1) |  | 256 (40.5) |  | 3,477 (32.5) |  | 10,253 (48.5) |
| Grade 3 | 73 (53.7) |  | 115 (18.2) |  | 5,024 (47.0) |  | 3,546 (16.8) |
| Unknown | 18 (13.2) |  | 153 (24.2) |  | 1,778 (16.6) |  | 2,615 (12.4) |
| **HER2 status** |  |  |  |  |  |  |  |
| Negative | 90 (66.2) |  | 482 (76.3) |  | 7,642 (71.5) |  | 16,938 (80.1) |
| Positive | 38 (27.9) |  | 41 (6.5) |  | 2,679 (25.1) |  | 1,208 (5.7) |
| Unknown/ unrecorded | 8 (5.9) |  | 109 (17.2) |  | 366 (3.4) |  | 2,989 (14.1) |
| **ER status** |  |  |  |  |  |  |  |
| Negative | 95 (69.9) |  | 500 (79.1) |  | 7,739 (72.4) |  | 17,684 (83.7) |
| Positive | 37 (27.2) |  | 56 (8.9) |  | 2,822 (26.4) |  | 1,408 (6.7) |
| Unknown | 4 (2.9) |  | 76 (12.0) |  | 126 (1.2) |  | 2,043 (9.7) |
| **PR status** |  |  |  |  |  |  |  |
| Negative | 74 (54.4) |  | 429 (67.9) |  | 6,294 (58.9) |  | 15,225 (72.0) |
| Positive | 58 (42.6) |  | 127 (20.1) |  | 4,251 (39.8) |  | 3,838 (18.2) |
| Unknown | 4 (2.9) |  | 76 (12.0) |  | 142 (1.3) |  | 2,072 (9.8) |
| **Menopausal status** |  |  |  |  |  |  |  |
| Pre-menopausal | 24 (17.6) |  | 17 (2.7) |  | 3,744 (35.0) |  | 2,804 (13.3) |
| Post-menopausal | 102 (75.0) |  | 593 (93.8) |  | 5,967 (55.8) |  | 16,914 (80.0) |
| Uncertain (e.g., hysterectomy) | 8 (5.9) |  | 13 (2.1) |  | 538 (5.0) |  | 763 (3.6) |
| Unknown | 2 (1.5) |  | 9 (1.4) |  | 438 (4.1) |  | 654 (3.1) |
| **Comorbidities at breast cancer diagnosis, *n (%)*** |  |  |  |  |  |  |  |
| Diabetes | 13 (9.6) |  | 95 (15.0) |  | 534 (5.0) |  | 1,858 (8.8) |
| CKD | 1 (0.7) |  | 19 (3.0) |  | 18 (0.2) |  | 192 (0.9) |
| COPD | 6 (4.4) |  | 56 (8.9) |  | 130 (1.2) |  | 748 (3.5) |
| **Breast cancer treatment in the year after diagnosis, *n (%)*** |  |  |  |  |  |  |  |
| **Surgery** |  |  |  |  |  |  |  |
| Primary operation | 116 (85.3) |  | 490 (77.5) |  | 8,949 (83.7) |  | 19,190 (90.8) |
| Pre-op oncological or conservative treatment | 20 (14.7) |  | 82 (13.0) |  | 1,724 (16.1) |  | 1,261 (6.0) |
| No surgery | 0 (0.0) |  | 60 (9.5) |  | 14 (0.1) |  | 679 (3.2) |
| Unknown | 0 (0.0) |  | 0 (0.0) |  | 0 (0.0) |  | 5 (0.0) |
| **Chemotherapy** |  |  |  |  |  |  |  |
| Anthracyclines | 136 (100.0) |  | 0 (0.0) |  | 10,687 (100.0) |  | 0 (0.0) |
| Docetaxel | 25 (18.4) |  | 1 (0.2) |  | 2,577 (24.1) |  | 89 (0.4) |
| Paclitaxel | 16 (11.8) |  | 3 (0.5) |  | 878 (8.2) |  | 156 (0.7) |
| Other chemotherapy | 4 (2.9) |  | 1 (0.2) |  | 419 (3.9) |  | 212 (1.0) |
| **Antibody therapy** | | | | | | | |
| Trastuzumab | 39 (28.7) |  | 6 (0.9) |  | 2,739 (25.6) |  | 365 (1.7) |
| Pertuzumab | 0 (0.0) |  | 0 (0.0) |  | 7 (0.1) |  | 0 (0.0) |
| **Endocrine therapy** | | | | | | | |
| Outpatient tamoxifen | 23 (16.9) |  | 108 (17.1) |  | 3,479 (32.6) |  | 8,343 (39.5) |
| Outpatient GNRH | 12 (8.8) |  | 5 (0.8) |  | 495 (4.6) |  | 175 (0.8) |
| Outpatient AI | 84 (61.8) |  | 433 (68.5) |  | 4,711 (44.1) |  | 9,456 (44.7) |
| **Radiotherapy** | | | | | | | |
| Not recorded | 27 (19.9) |  | 414 (65.5) |  | 1,785 (16.7) |  | 9,705 (45.9) |
| Right breast | 53 (39.0) |  | 109 (17.2) |  | 4,418 (41.3) |  | 5,589 (26.4) |
| Left breast | 56 (41.2) |  | 109 (17.2) |  | 4,484 (42.0) |  | 5,841 (27.6) |

AI = aromatase inhibitors; COPD = chronic obstructive pulmonary disease; CKD = chronic kidney disease; CAD = coronary artery disease; ER = oestrogen receptor; GNRH = Gonadotropin-releasing hormone; HER2 = Human Epidermal Growth Factor Receptor 2; IQR = interquartile range; n/a = not applicable; PR = progesterone receptor; SD = standard deviation; TNM = UICC TNM Classification of Malignant Tumours; VTE = venous thromboembolism.

## Supplementary Table 8 Patient and tumour characteristics of patients with and without prior CVD, except for hypertension, that received/did not receive anthracyclines. (Please note that patients with hypertension as well as other CVDs are still included.)

|  | **Breast cancer patients with a history of CVD exc. hypertension at diagnosis** (N=8,337) | | |  | **Breast cancer patients with a history of CVD exc. hypertension** (N=24,253) | | |
| --- | --- | --- | --- | --- | --- | --- | --- |
|  | **Anthracyclines**  (N=1,612, 19.3%) |  | **No anthracyclines**  (N=6,725, 80.7%) |  | **Anthracyclines**  (N=9,211, 37.9%) |  | **No anthracyclines**  (N=15,042, 62.0%) |
| **Type of cardiovascular disease, *n (%)*** | | | | |  |  |  |
| CAD | 198 (12.3) |  | 1,661 (24.7) |  | 0 (0.0) |  | 0 (0.0) |
| Heart failure | 42 (2.6) |  | 1,110 (16.5) |  | 0 (0.0) |  | 0 (0.0) |
| Hypertension | 448 (27.8) |  | 3,431 (51.0) |  | 611 (6.6) |  | 1,966 (13.1) |
| Stroke | 131 (8.1) |  | 1,009 (15.0) |  | 0 (0.0) |  | 0 (0.0) |
| VTE | 136 (8.4) |  | 632 (9.4) |  | 0 (0.0) |  | 0 (0.0) |
| **Demographics** |  |  |  |  |  |  |  |
| **Age at diagnosis (years)** |  |  |  |  |  |  |  |
| Mean (SD) | 60.2 (10.7) |  | 74.2 (11.9) |  | 54.3 (11.5) |  | 64.3 (12.9) |
| Median (IQR) | 62.0 (52.0, 68.0) |  | 75.0 (67.0, 83.0) |  | 54.0 (46.0, 63.0) |  | 65.0 (55.0, 73.0) |
| **Age group at diagnosis (years), *n (%)*** |  |  |  |  |  |  |  |
| 18 to 39 | 68 (4.2) |  | 26 (0.4) |  | 983 (10.7) |  | 326 (2.2) |
| 40 to 59 | 597 (37.0) |  | 708 (10.5) |  | 4,892 (53.1) |  | 4,806 (32.0) |
| 60 to 79 | 934 (57.9) |  | 3,461 (51.5) |  | 3,314 (36.0) |  | 7,920 (52.7) |
| 80 plus | 13 (0.8) |  | 2,530 (37.6) |  | 22 (0.2) |  | 1,990 (13.2) |
| **Year of breast cancer diagnosis, *n (%)*** |  |  |  |  |  |  |  |
| 2010 to 2011 | 326 (20.2) |  | 1,770 (26.3) |  | 2,186 (23.7) |  | 4,629 (30.8) |
| 2012 to 2013 | 552 (34.2) |  | 2,202 (32.7) |  | 3,316 (36.0) |  | 4,549 (30.2) |
| 2014 to 2015 | 734 (45.5) |  | 2,753 (40.9) |  | 3,709 (40.3) |  | 5,864 (39.0) |
| **Region of Sweden, *n (%)*** |  |  |  |  |  |  |  |
| North | 143 (8.9) |  | 503 (7.5) |  | 772 (8.4) |  | 1,071 (7.1) |
| South | 339 (21.0) |  | 1,262 (18.8) |  | 1,706 (18.5) |  | 2,704 (18.0) |
| Southeast | 186 (11.5) |  | 663 (9.9) |  | 943 (10.2) |  | 1,401 (9.3) |
| Stockholm/Gotland | 406 (25.2) |  | 1,380 (20.5) |  | 2,453 (26.6) |  | 2,902 (19.3) |
| Uppsala/Örebro | 282 (17.5) |  | 1,479 (22.0) |  | 1,633 (17.7) |  | 3,532 (23.5) |
| West | 256 (15.9) |  | 1,438 (21.4) |  | 1,704 (18.5) |  | 3,432 (22.8) |
| **Civil status, *n (%)*** |  |  |  |  |  |  |  |
| Unmarried | 227 (14.1) |  | 571 (8.5) |  | 2,153 (23.4) |  | 2,352 (15.6) |
| Married | 908 (56.3) |  | 2,746 (40.8) |  | 5,048 (54.8) |  | 7,694 (51.2) |
| Divorced/separated | 346 (21.5) |  | 1,233 (18.3) |  | 1,581 (17.2) |  | 2,759 (18.3) |
| Widow | 131 (8.1) |  | 2,175 (32.3) |  | 426 (4.6) |  | 2,234 (14.9) |
| Unknown | 227 (14.1) |  | 571 (8.5) |  | 3 (0.0) |  | 3 (0.0) |
| **Level of education, *n (%)*** |  |  |  |  |  |  |  |
| Compulsory education or less | 308 (19.1) |  | 2,644 (39.3) |  | 1,274 (13.8) |  | 3,672 (24.4) |
| Upper secondary | 738 (45.8) |  | 2,519 (37.5) |  | 3,945 (42.8) |  | 6,192 (41.2) |
| College/ University/ Research | 558 (34.6) |  | 1,476 (21.9) |  | 3,944 (42.8) |  | 5,036 (33.5) |
| Unknown | 8 (0.5) |  | 86 (1.3) |  | 48 (0.5) |  | 142 (0.9) |
| **Disposable income SEK, *N (%)*** |  |  |  |  |  |  |  |
| 1 to 1,000 | 150 (9.3) |  | 944 (14.0) |  | 666 (7.2) |  | 1,606 (10.7) |
| 1,001 to 2,000 | 687 (42.6) |  | 4,290 (63.8) |  | 3,002 (32.6) |  | 7,005 (46.6) |
| 2,001 to 3,000 | 500 (31.0) |  | 939 (14.0) |  | 3,439 (37.3) |  | 4,024 (26.8) |
| > 3,000 | 270 (16.7) |  | 539 (8.0) |  | 1,992 (21.6) |  | 2,295 (15.3) |
| Unknown | 5 (0.3) |  | 13 (0.2) |  | 112 (1.2) |  | 112 (0.7) |
| **Tumour characteristics, *n (%)*** | | | | |  |  |  |
| **TNM stage** |  |  |  |  |  |  |  |
| Stage 1 | 656 (40.7) |  | 3,570 (53.1) |  | 3,796 (41.2) |  | 9,943 (66.1) |
| Stage 2 | 861 (53.4) |  | 2,860 (42.5) |  | 4,846 (52.6) |  | 4,695 (31.2) |
| Stage 3 | 95 (5.9) |  | 295 (4.4) |  | 569 (6.2) |  | 404 (2.7) |
| **Histological grade** |  |  |  |  |  |  |  |
| Grade 1 | 58 (3.6) |  | 1,236 (18.4) |  | 354 (3.8) |  | 3,593 (23.9) |
| Grade 2 | 527 (32.7) |  | 2,864 (42.6) |  | 2,991 (32.5) |  | 7,645 (50.8) |
| Grade 3 | 802 (49.8) |  | 1,256 (18.7) |  | 4,295 (46.6) |  | 2,405 (16.0) |
| Unknown | 225 (14.0) |  | 1,369 (20.4) |  | 1,571 (17.1) |  | 1,399 (9.3) |
| **HER2 status** |  |  |  |  |  |  |  |
| Negative | 1,130 (70.1) |  | 5,137 (76.4) |  | 6,602 (71.7) |  | 12,283 (81.7) |
| Positive | 421 (26.1) |  | 430 (6.4) |  | 2,296 (24.9) |  | 819 (5.4) |
| Unknown/ unrecorded | 61 (3.8) |  | 1,158 (17.2) |  | 313 (3.4) |  | 1,940 (12.9) |
| **ER status** |  |  |  |  |  |  |  |
| Negative | 1,124 (69.7) |  | 5,371 (79.9) |  | 6,710 (72.8) |  | 12,813 (85.2) |
| Positive | 470 (29.2) |  | 557 (8.3) |  | 2,389 (25.9) |  | 907 (6.0) |
| Unknown | 18 (1.1) |  | 797 (11.9) |  | 112 (1.2) |  | 1,322 (8.8) |
| **PR status** |  |  |  |  |  |  |  |
| Negative | 897 (55.6) |  | 4,591 (68.3) |  | 5,471 (59.4) |  | 11,063 (73.5) |
| Positive | 691 (42.9) |  | 1,326 (19.7) |  | 3,618 (39.3) |  | 2,639 (17.5) |
| Unknown | 24 (1.5) |  | 808 (12.0) |  | 122 (1.3) |  | 1,340 (8.9) |
| **Menopausal status** |  |  |  |  |  |  |  |
| Pre-menopausal | 324 (20.1) |  | 290 (4.3) |  | 3,444 (37.4) |  | 2,531 (16.8) |
| Post-menopausal | 1,142 (70.8) |  | 6,151 (91.5) |  | 4,927 (53.5) |  | 11,356 (75.5) |
| Unknown | 146 (9.1) |  | 284 (1.9) |  | 840 (9.1) |  | 1155 (7.7) |
| **Comorbidities at breast cancer diagnosis, *n (%)*** |  |  |  |  |  |  |  |
| Diabetes | 152 (9.4) |  | 1,066 (15.9) |  | 395 (4.3) |  | 887 (5.9) |
| CKD | 9 (0.6) |  | 171 (2.5) |  | 10 (0.1) |  | 40 (0.3) |
| COPD | 50 (3.1) |  | 490 (7.3) |  | 86 (0.9) |  | 314 (2.1) |
| **Breast cancer treatment in the year after diagnosis, *n (%)*** |  |  |  |  |  |  |  |
| **Surgery** |  |  |  |  |  |  |  |
| Primary operation | 1,390 (86.2) |  | 5,529 (82.2) |  | 7,675 (83.3) |  | 14,151 (94.1) |
| Pre-op oncological or conservative treatment | 221 (13.7) |  | 739 (11.0) |  | 1,523 (16.5) |  | 604 (4.0) |
| No surgery | 1 (0.1) |  | 455 (6.8) |  | 13 (0.1) |  | 284 (1.9) |
| Unknown | 0 (0.0) |  | 2 (0.0) |  | 0 (0.0) |  | 3 (0.0) |
| **Chemotherapy** |  |  |  |  |  |  |  |
| Anthracyclines | 1,612 (100.0) |  | 0 (0.0) |  | 9,211 (100.0) |  | 0 (0.0) |
| Docetaxel | 380 (23.6) |  | 24 (0.4) |  | 2,222 (24.1) |  | 66 (0.4) |
| Paclitaxel | 171 (10.6) |  | 49 (0.7) |  | 723 (7.8) |  | 110 (0.7) |
| Other chemotherapy | 69 (4.3) |  | 59 (0.9) |  | 354 (3.8) |  | 154 (1.0) |
| **Antibody therapy** | | | | | | | |
| Trastuzumab | 418 (25.9) |  | 90 (1.3) |  | 2,360 (25.6) |  | 281 (1.9) |
| Pertuzumab | 1 (0.1) |  | 0 (0.0) |  | 6 (0.1) |  | 0 (0.0) |
| **Endocrine therapy** | | | | | | | |
| Outpatient tamoxifen | 341 (21.2) |  | 1,865 (27.7) |  | 3,161 (34.3) |  | 6,586 (43.8) |
| Outpatient GNRH | 55 (3.4) |  | 32 (0.5) |  | 452 (4.9) |  | 148 (1.0) |
| Outpatient AI | 847 (52.5) |  | 3,919 (58.3) |  | 3,948 (42.9) |  | 5,970 (39.7) |
| **Radiotherapy** | | | | | | | |
| Not recorded | 308 (19.1) |  | 3,917 (58.2) |  | 1,504 (16.3) |  | 6,202 (41.2) |
| Right breast | 642 (39.8) |  | 1,384 (20.6) |  | 3,829 (41.6) |  | 4,314 (28.7) |
| Left breast | 662 (41.1) |  | 1,424 (21.2) |  | 3,878 (42.1) |  | 4,526 (30.1) |

AI = aromatase inhibitors; COPD = chronic obstructive pulmonary disease; CKD = chronic kidney disease; CAD = coronary artery disease; ER = oestrogen receptor; GNRH = Gonadotropin-releasing hormone; HER2 = Human Epidermal Growth Factor Receptor 2; IQR = interquartile range; n/a = not applicable; PR = progesterone receptor; SD = standard deviation; TNM = UICC TNM Classification of Malignant Tumours; VTE = venous thromboembolism.

## Supplementary Table 9 Characteristics of patients with and without prior CVD that received/did not receive anthracyclines, stratified by neo-adjuvant and adjuvant setting.

|  | **Breast cancer patients with a history of CVD at diagnosis*** (N=10,702) | | | |  | **Breast cancer patients with no history of CVD at diagnosis*** (N=21,888) | | | |
| --- | --- | --- | --- | --- | --- | --- | --- | --- | --- |
|  | **No anthracyclines**  (N=8,533, 79.7%) | **Anthracyclines, any setting †**  (N=2,169, 20.3%) | **Anthracyclines, neo-adjuvant setting †**  (N=285, 13.1%) | **Anthracyclines, adjuvant**  **setting †**  (N=1,870, 86.2%) |  | **No anthracyclines**  (N=13,234, 60.5%) | **Anthracyclines, any setting †**  (N=8,654, 39.5%) | **Anthracyclines, neo-adjuvant setting †**  (N=1,430, 16.5%) | **Anthracyclines, adjuvant**  **setting †**  (N=7,203, 83.2%) |
| **Type of cardiovascular disease, *n (%)*** | | | | | |  |  |  |  |
| CAD | 1,661 (19.5) | 198 (9.1) | 25 (8.8) | 173 (9.3) |  | 0 (0.0) | 0 (0.0) | 0 (0.0) | 0 (0.0) |
| Heart failure | 1,110 (13.0) | 42 (1.9) | 6 (2.1) | 36 (1.9) |  | 0 (0.0) | 0 (0.0) | 0 (0.0) | 0 (0.0) |
| Hypertension | 5,397 (63.2) | 1,059 (48.8) | 128 (44.9) | 923 (49.4) |  | 0 (0.0) | 0 (0.0) | 0 (0.0) | 0 (0.0) |
| Stroke | 1,009 (11.8) | 131 (6.0) | 10 (3.5) | 117 (6.3) |  | 0 (0.0) | 0 (0.0) | 0 (0.0) | 0 (0.0) |
| **Demographics** |  |  |  |  |  |  |  |  |  |
| **Age at diagnosis (years)** |  |  |  |  |  |  |  |  |  |
| Mean (SD) | 73.8 (11.6) | 61.1 (10.2) | 58.8 (11.4) | 61.5 (10.0) |  | 63.2 (12.8) | 53.7 (11.4) | 50.7 (11.6) | 54.3 (11.2) |
| Median (IQR) | 74.0 (67.0, 83.0) | 63.0 (54.0, 69.0) | 61.0 (50.0, 67.0) | 63.0 (55.0, 69.0) |  | 64.0 (53.0, 72.0) | 54.0 (45.0, 63.0) | 50.0 (42.0, 59.0) | 54.0 (46.0, 63.0) |
| **Age group at diagnosis (years), *n (%)*** |  |  |  |  |  |  |  |  |  |
| 18 to 39 | 26 (0.3) | 75 (3.5) | 19 (6.7) | 56 (3.0) |  | 326 (2.5) | 976 (11.3) | 262 (18.3) | 710 (9.9) |
| 40 to 59 | 884 (10.4) | 740 (34.1) | 113 (39.6) | 623 (33.3) |  | 4,630 (35.0) | 4,749 (54.9) | 820 (57.3) | 3,920 (54.4) |
| 60 to 79 | 4,609 (54.0) | 1,335 (61.5) | 149 (52.3) | 1,176 (62.9) |  | 6,772 (51.2) | 2,913 (33.7) | 344 (24.1) | 2,561 (35.6) |
| 80 plus | 3,014 (35.3) | 19 (0.9) | 4 (1.4) | 15 (0.8) |  | 1,506 (11.4) | 16 (0.2) | 4 (0.3) | 12 (0.2) |
| **Year of breast cancer diagnosis, *n (%)*** |  |  |  |  |  |  |  |  |  |
| 2010 to 2011 | 2,215 (26.0) | 444 (20.5) | 44 (15.4) | 393 (21.0) |  | 4,184 (31.6) | 2,068 (23.9) | 274 (19.2) | 1,789 (24.8) |
| 2012 to 2013 | 2,780 (32.6) | 753 (34.7) | 70 (24.6) | 678 (36.3) |  | 3,971 (30.0) | 3,115 (36.0) | 440 (30.8) | 2,664 (37.0) |
| 2014 to 2015 | 3,538 (41.5) | 972 (44.8) | 171 (60.0) | 799 (42.7) |  | 5,079 (38.4) | 3,471 (40.1) | 716 (50.1) | 2,750 (38.2) |
| **Region of Sweden, *n (%)*** |  |  |  |  |  |  |  |  |  |
| North | 644 (7.5) | 206 (9.5) | 20 (7.0) | 184 (9.8) |  | 930 (7.0) | 709 (8.2) | 92 (6.4) | 612 (8.5) |
| South | 1,604 (18.8) | 427 (19.7) | 79 (27.7) | 349 (18.7) |  | 2,362 (17.8) | 1,618 (18.7) | 337 (23.6) | 1,279 (17.8) |
| Southeast | 834 (9.8) | 243 (11.2) | 30 (10.5) | 212 (11.3) |  | 1,230 (9.3) | 886 (10.2) | 106 (7.4) | 767 (10.6) |
| Stockholm/Gotland | 1,720 (20.2) | 546 (25.2) | 91 (31.9) | 454 (24.3) |  | 2,562 (19.4) | 2,313 (26.7) | 552 (38.6) | 1,768 (24.5) |
| Uppsala/Örebro | 1,887 (22.1) | 386 (17.8) | 26 (9.1) | 353 (18.9) |  | 3,124 (23.6) | 1,529 (17.7) | 160 (11.2) | 1,359 (18.9) |
| West | 1,844 (21.6) | 361 (16.6) | 39 (13.7) | 318 (17.0) |  | 3,026 (22.9) | 1,599 (18.5) | 183 (12.8) | 1,418 (19.7) |
| **Civil status, *n (%)*** |  |  |  |  |  |  |  |  |  |
| Unmarried | 743 (8.7) | 303 (14.0) | 38 (13.3) | 262 (14.0) |  | 2,180 (16.5) | 2,077 (24.0) | 428 (29.9) | 1,643 (22.8) |
| Married | 3,601 (42.2) | 1,227 (56.6) | 173 (60.7) | 1,051 (56.2) |  | 6,839 (51.7) | 4,729 (54.6) | 702 (49.1) | 4,016 (55.8) |
| Divorced/separated | 1,540 (18.0) | 455 (21.0) | 53 (18.6) | 397 (21.2) |  | 2,452 (18.5) | 1,472 (17.0) | 249 (17.4) | 1,219 (16.9) |
| Widow | 2,649 (31.0) | 184 (8.5) | 21 (7.4) | 160 (8.6) |  | 1,760 (13.3) | 373 (4.3) | 50 (3.5) | 323 (4.5) |
| Unknown | 743 (8.7) | 303 (14.0) | 38 (13.3) | 262 (14.0) |  | 3 (0.0) | 3 (0.0) | 1 (0.1) | 2 (0.0) |
| **Level of education, *n (%)*** |  |  |  |  |  |  |  |  |  |
| Compulsory education or less | 3,269 (38.3) | 409 (18.9) | 54 (18.9) | 351 (18.8) |  | 3,047 (23.0) | 1,173 (13.6) | 192 (13.4) | 979 (13.6) |
| Upper secondary | 3,243 (38.0) | 1,006 (46.4) | 117 (41.1) | 882 (47.2) |  | 5,468 (41.3) | 3,677 (42.5) | 580 (40.6) | 3,088 (42.9) |
| College/ University/ Research | 1,915 (22.4) | 740 (34.1) | 111 (38.9) | 628 (33.6) |  | 4,597 (34.7) | 3,762 (43.5) | 648 (45.3) | 3,104 (43.1) |
| Unknown | 106 (1.2) | 14 (0.6) | 3 (1.1) | 9 (0.5) |  | 122 (0.9) | 42 (0.5) | 10 (0.7) | 32 (0.4) |
| **Disposable income SEK, *n (%)*** |  |  |  |  |  |  |  |  |  |
| 1 to 1,000 | 1,214 (14.2) | 207 (9.5) | 25 (8.8) | 179 (9.6) |  | 1,336 (10.1) | 609 (7.0) | 126 (8.8) | 481 (6.7) |
| 1,001 to 2,000 | 5,380 (63.0) | 953 (43.9) | 118 (41.4) | 829 (44.3) |  | 5,915 (44.7) | 2,736 (31.6) | 405 (28.3) | 2,324 (32.3) |
| 2,001 to 3,000 | 1,226 (14.4) | 649 (29.9) | 100 (35.1) | 547 (29.3) |  | 3,737 (28.2) | 3,290 (38.0) | 522 (36.5) | 2,757 (38.3) |
| > 3,000 | 697 (8.2) | 351 (16.2) | 41 (14.4) | 307 (16.4) |  | 2,137 (16.1) | 1,911 (22.1) | 356 (24.9) | 1,557 (21.6) |
| Unknown | 16 (0.2) | 9 (0.4) | 1 (0.4) | 8 (0.4) |  | 109 (0.8) | 108 (1.2) | 21 (1.5) | 84 (1.2) |
| **Tumour characteristics, *n (%)*** | | | | | |  |  |  |  |
| **TNM stage** |  |  |  |  |  |  |  |  |  |
| Stage 1 | 4,635 (54.3) | 874 (40.3) | 9 (3.2) | 859 (45.9) |  | 8,878 (67.1) | 3,578 (41.3) | 53 (3.7) | 3,509 (48.7) |
| Stage 2 | 3,533 (41.4) | 1,164 (53.7) | 188 (66.0) | 968 (51.8) |  | 4,022 (30.4) | 4,543 (52.5) | 982 (68.7) | 3,556 (49.4) |
| Stage 3 | 365 (4.3) | 131 (6.0) | 88 (30.9) | 43 (2.3) |  | 334 (2.5) | 533 (6.2) | 395 (27.6) | 138 (1.9) |
| **Histological grade** |  |  |  |  |  |  |  |  |  |
| Grade 1 | 1,573 (18.4) | 75 (3.5) | 2 (0.7) | 70 (3.7) |  | 3,256 (24.6) | 337 (3.9) | 2 (0.1) | 333 (4.6) |
| Grade 2 | 3,773 (44.2) | 712 (32.8) | 5 (1.8) | 704 (37.6) |  | 6,736 (50.9) | 2,806 (32.4) | 28 (2.0) | 2,767 (38.4) |
| Grade 3 | 1,580 (18.5) | 1,079 (49.7) | 2 (0.7) | 1,071 (57.3) |  | 2,081 (15.7) | 4,018 (46.4) | 30 (2.1) | 3,973 (55.2) |
| Unknown | 1,607 (18.8) | 303 (14.0) | 276 (96.8) | 25 (1.3) |  | 1,161 (8.8) | 1,493 (17.3) | 1,370 (95.8) | 130 (1.8) |
| **HER2 status** |  |  |  |  |  |  |  |  |  |
| Negative | 6,580 (77.1) | 1,535 (70.8) | 183 (64.2) | 1,346 (72.0) |  | 10,840 (81.9) | 6,197 (71.6) | 891 (62.3) | 5,289 (73.4) |
| Positive | 544 (6.4) | 556 (25.6) | 75 (26.3) | 477 (25.5) |  | 705 (5.3) | 2,161 (25.0) | 403 (28.2) | 1,757 (24.4) |
| Unknown/unrecorded | 1,409 (16.5) | 78 (3.6) | 27 (9.5) | 47 (2.5) |  | 1,689 (12.8) | 296 (3.4) | 136 (9.5) | 157 (2.2) |
| **ER status** |  |  |  |  |  |  |  |  |  |
| Negative | 6,887 (80.7) | 1,527 (70.4) | 170 (59.6) | 1,347 (72.0) |  | 11,297 (85.4) | 6,307 (72.9) | 903 (63.1) | 5,387 (74.8) |
| Positive | 685 (8.0) | 621 (28.6) | 100 (35.1) | 520 (27.8) |  | 779 (5.9) | 2,238 (25.9) | 440 (30.8) | 1,797 (24.9) |
| Unknown | 961 (11.3) | 21 (1.0) | 15 (5.3) | 3 (0.2) |  | 1,158 (8.8) | 109 (1.3) | 87 (6.1) | 19 (0.3) |
| **PR status** |  |  |  |  |  |  |  |  |  |
| Negative | 5,852 (68.6) | 1,219 (56.2) | 120 (42.1) | 1,093 (58.4) |  | 9,802 (74.1) | 5,149 (59.5) | 679 (47.5) | 4,457 (61.9) |
| Positive | 1,708 (20.0) | 922 (42.5) | 148 (51.9) | 769 (41.1) |  | 2,257 (17.1) | 3,387 (39.1) | 661 (46.2) | 2,721 (37.8) |
| Unknown | 973 (11.4) | 28 (1.3) | 17 (6.0) | 8 (0.4) |  | 1,175 (8.9) | 118 (1.4) | 90 (6.3) | 25 (0.3) |
| **Menopausal status** |  |  |  |  |  |  |  |  |  |
| Pre-menopausal | 334 (3.9) | 364 (16.8) | 71 (24.9) | 292 (15.6) |  | 2,487 (18.8) | 3,404 (39.3) | 703 (49.2) | 2,690 (37.3) |
| Post-menopausal | 7,825 (91.7) | 1,611 (74.3) | 183 (64.2) | 1,418 (75.8) |  | 9,682 (73.2) | 4,458 (51.5) | 617 (43.1) | 3,830 (53.2) |
| Unknown | 374 (4.4) | 194 (8.9) | 31 (10.9) | 160 (8.5) |  | 1065 (8.0) | 792 (9.2) | 110 (7.7) | 683 (9.5) |
| **Comorbidities at breast cancer diagnosis, *n (%)*** |  |  |  |  |  |  |  |  |  |
| Diabetes | 1,390 (16.3) | 270 (12.4) | 43 (15.1) | 225 (12.0) |  | 563 (4.3) | 277 (3.2) | 46 (3.2) | 229 (3.2) |
| CKD | 196 (2.3) | 12 (0.6) | 0 (0.0) | 12 (0.6) |  | 15 (0.1) | 7 (0.1) | 2 (0.1) | 5 (0.1) |
| COPD | 588 (6.9) | 67 (3.1) | 8 (2.8) | 59 (3.2) |  | 216 (1.6) | 69 (0.8) | 10 (0.7) | 60 (0.8) |
| **Breast cancer treatment in the year after diagnosis, *n (%)*** |  |  |  |  |  |  |  |  |  |
| **Surgery** |  |  |  |  |  |  |  |  |  |
| Primary operation | 7,144 (83.7) | 1,870 (86.2) | 3 (1.1) | 1,855 (99.2) |  | 12,536 (94.7) | 7,195 (83.1) | 24 (1.7) | 7,141 (99.1) |
| Pre-op oncological or conservative treatment | 876 (10.3) | 298 (13.7) | 281 (98.6) | 15 (0.8) |  | 467 (3.5) | 1,446 (16.7) | 1,394 (97.5) | 62 (0.9) |
| No surgery | 510 (6.0) | 1 (0.0) | 1 (0.4) | 0 (0.0) |  | 229 (1.7) | 13 (0.2) | 12 (0.8) | 0 (0.0) |
| Unknown | 3 (0.0) | 0 (0.0) | 0 (0.0) | 0 (0.0) |  | 2 (0.0) | 0 (0.0) | 0 (0.0) | 0 (0.0) |
| **Chemotherapy** |  |  |  |  |  |  |  |  |  |
| Anthracyclines | 0 (0.0) | 2,169 (100.0) | 285 (100.0) | 1,870 (100.0) |  | 0 (0.0) | 8,654 (100.0) | 1,430 (100.0) | 7,203 (100.0) |
| Docetaxel | 30 (0.4) | 508 (23.4) | 102 (35.8) | 400 (21.4) |  | 60 (0.5) | 2,094 (24.2) | 539 (37.7) | 1,554 (21.6) |
| Paclitaxel | 58 (0.7) | 227 (10.5) | 38 (13.3) | 188 (10.1) |  | 101 (0.8) | 667 (7.7) | 96 (6.7) | 571 (7.9) |
| Other chemotherapy | 77 (0.9) | 86 (4.0) | 28 (9.8) | 58 (3.1) |  | 136 (1.0) | 337 (3.9) | 131 (9.2) | 203 (2.8) |
| **Antibody therapy** | | | | | | | | | |
| Trastuzumab | 119 (1.4) | 556 (25.6) | 85 (29.8) | 465 (24.9) |  | 252 (1.9) | 2,222 (25.7) | 474 (33.1) | 1,749 (24.3) |
| Pertuzumab | 0 (0.0) | 2 (0.1) | 2 (0.7) | 0 (0.0) |  | 0 (0.0) | 5 (0.1) | 3 (0.2) | 2 (0.0) |
| **Endocrine therapy** | | | | | | | | | |
| Outpatient tamoxifen | 2,527 (29.6) | 408 (18.8) | 53 (18.6) | 354 (18.9) |  | 5,924 (44.8) | 3,094 (35.8) | 512 (35.8) | 2,572 (35.7) |
| Outpatient GNRH | 36 (0.4) | 55 (2.5) | 12 (4.2) | 43 (2.3) |  | 144 (1.1) | 452 (5.2) | 130 (9.1) | 321 (4.5) |
| Outpatient AI | 4,861 (57.0) | 1,196 (55.1) | 127 (44.6) | 1,061 (56.7) |  | 5,028 (38.0) | 3,599 (41.6) | 485 (33.9) | 3,107 (43.1) |
| **Radiotherapy** | | | | | | | | | |
| Not recorded | 4,780 (56.0) | 390 (18.0) | 23 (8.1) | 362 (19.4) |  | 5,339 (40.3) | 1,422 (16.4) | 96 (6.7) | 1,315 (18.3) |
| Right breast | 1,847 (21.6) | 857 (39.5) | 129 (45.3) | 725 (38.8) |  | 3,851 (29.1) | 3,614 (41.8) | 659 (46.1) | 2,945 (40.9) |
| Left breast | 1,906 (22.3) | 922 (42.5) | 133 (46.7) | 783 (41.9) |  | 4,044 (30.6) | 3,618 (41.8) | 675 (47.2) | 2,943 (40.9) |

AI = aromatase inhibitors; COPD = chronic obstructive pulmonary disease; CKD = chronic kidney disease; CAD = coronary artery disease; ER = oestrogen receptor; GNRH = Gonadotropin-releasing hormone; HER2 = Human Epidermal Growth Factor Receptor 2; IQR = interquartile range; n/a = not applicable; PR = progesterone receptor; SD = standard deviation; TNM = UICC TNM Classification of Malignant Tumours.

* CVD history defined as having one or more of the following conditions at breast cancer diagnosis: coronary heart disease (CAD), heart failure, primary hypertension and stroke.

† In patients with CVD: information on whether the chemotherapy was administered in the neoadjuvant or adjuvant setting was missing for 14 patients due to missing surgery date. Three patients had chemotherapy both in the neoadjuvant and adjuvant setting; these were included in both analysis.

## Supplementary Table 10 Characteristics of patients with and without prior CVD that received/did not receive anthracyclines, stratified by breast cancer subtype.

|  | **Breast cancer patients with a history of CVD at diagnosis*** (N=10,702) | | | |  | **Breast cancer patients with no history of CVD at diagnosis*** (N=21,888) | | | |
| --- | --- | --- | --- | --- | --- | --- | --- | --- | --- |
|  | **No anthracyclines**  (N=8,533, 79.7%) | **Anthracyclines, luminal †**  (N=1,149) | **Anthracyclines, HER2+ †**  (N=556) | **Anthracyclines, Triple negative †**  (N=386) |  | **No anthracyclines**  (N=13,234, 60.5%) | **Anthracyclines, luminal †**  (N=4,805) | **Anthracyclines, HER2+ †**  (N=2,161) | **Anthracyclines, Triple negative †**  (N=1,392) |
| **Type of cardiovascular disease, *n (%)*** | | | | |  |  |  |  |  |
| CAD | 1,661 (19.5) | 88 (7.6) | 51 (9.2) | 50 (13.0) |  | 0 (0.0) | 0 (0.0) | 0 (0.0) | 0 (0.0) |
| Heart failure | 1,110 | 17 (1.5) | 14 (2.5) | 7 (1.8) |  | 0 (0.0) | 0 (0.0) | 0 (0.0) | 0 (0.0) |
| Hypertension | 1,059 (48.8) | 576 (50.1) | 254 (45.7) | 201 (52.1) |  | 0 (0.0) | 0 (0.0) | 0 (0.0) | 0 (0.0) |
| Stroke | 198 (9.1) | 70 (6.1) | 28 (5.0) | 26 (6.7) |  | 0 (0.0) | 0 (0.0) | 0 (0.0) | 0 (0.0) |
| **Demographics** |  |  |  |  |  |  |  |  |  |
| **Age at diagnosis (years)** |  |  |  |  |  |  |  |  |  |
| Mean (SD) | 73.8 (11.6) | 60.9 (10.2) | 61.2 (9.9) | 61.8 (10.9) |  | 63.2 (12.8) | 53.9 (10.9) | 53.6 (11.6) | 53.3 (12.4) |
| Median (IQR) | 74 (67-83) | 63 (54-68) | 63 (55-68) | 64 (55-70) |  | 64 (53-72) | 53 (46-63) | 54 (45-62) | 54 (44-63) |
| **Age group at diagnosis (years), *n (%)*** |  |  |  |  |  |  |  |  |  |
| 18 to 39 | 20 (0.3) | 32 (2.8) | 19 (3.4) | 20 (5.2) |  | 326 (2.5) | 441 (9.2) | 271 (12.5) | 223 (16.0) |
| 40 to 59 | 884 (10.4) | 400 (34.8) | 189 (34.0) | 122 (31.6) |  | 4,630 (35.0) | 2,728 (56.8) | 1,186 (54.9) | 680 (48.9) |
| 60 to 79 | 4,609 (54.0) | 707 (61.5) | 343 (61.7) | 240 (62.2) |  | 6,772 (51.2) | 1,633 (34.0) | 696 (32.2) | 486 (34.9) |
| 80 plus | 3,014 (35.3) | 10 (0.9) | 5 (0.9) | 4 (1.0) |  | 1,506 (11.4) | 3 (0.1) | 8 (0.4) | 3 (0.2) |
| **Year of breast cancer diagnosis, *n (%)*** |  |  |  |  |  |  |  |  |  |
| 2010 to 2011 | 2,215 (26.0) | 226 (19.7) | 125 (22.5) | 75 (19.4) |  | 4,184 (31.6) | 1,121 (23.3) | 500 (23.1) | 353 (25.3) |
| 2012 to 2013 | 2,780 (32.6) | 420 (36.6) | 172 (30.9) | 137 (35.5) |  | 3,971 (30.0) | 1,763 (36.7) | 780 (36.1) | 451 (32.4) |
| 2014 to 2015 | 3,538 (41.5) | 503 (43.8) | 259 (46.6) | 174 (45.1) |  | 5,079 (38.4) | 1,921 (40.0) | 881 (40.8) | 589 (42.3) |
| **Region of Sweden, *n (%)*** |  |  |  |  |  |  |  |  |  |
| North | 644 (7.55) | 119 (10.4) | 39 (7.0) | 42 (10.9) |  | 930 (7.0) | 398 (8.3) | 173 (8.0) | 122 (8.8) |
| South | 1,604 (18.8) | 207 (18.0) | 106 (19.1) | 87 (22.5) |  | 2,362 (17.9) | 863 (18.0) | 390 (18.1) | 241 (17.3) |
| Southeast | 834 (9.8) | 139 (12.1) | 59 (10.6) | 36 (9.3) |  | 1,230 (9.3) | 498 (10.4) | 230 (10.6) | 131 (9.4) |
| Stockholm/Gotland | 1,720 (20.2) | 322 (28.0) | 124 (22.3) | 82 (21.2) |  | 2,562 (19.4) | 1,398 (20.1) | 535 (24.8) | 312 (22.4) |
| Uppsala/Örebro | 1,887 (22.1) | 191 (16.6) | 114 (20.5) | 69 (17.9) |  | 3,124 (23.6) | 842 (17.5) | 406 (18.8) | 255 (18.3) |
| West | 1,844 (21.6) | 171 (14.9) | 114 (20.5) | 70 (18.1) |  | 3,026 (22.9) | 806 (16.8) | 427 (19.8) | 331 (23.8) |
| **Civil status, *n (%)*** |  |  |  |  |  |  |  |  |  |
| Unmarried | 743 (8.7) | 164 (14.3) | 72 (13.0) | 49 (12.7) |  | 2,180 (16.5) | 1,115 (23.2) | 540 (25.0) | 357 (25.7) |
| Married | 3,601 (42.2) | 649 (56.5) | 325 (58.5) | 210 (54.4) |  | 6,839 (51.7) | 2,692 (56.0) | 1,146 (53.0) | 730 (52.4) |
| Divorced/separated | 1,540 (18.1) | 256 (22.3) | 102 (18.4) | 83 (21.5) |  | 2,452 (18.5) | 795 (16.6) | 374 (17.3) | 253 (18.2) |
| Widow | 2, 649 (31.0) | 80 (7.0) | 57 (10.3) | 44 (11.4) |  | 1,760 (13.3) | 201 (4.2) | 101 (4.7) | 51 (3.7) |
| Unknown | 0 (0.0) | 0 (0.0) | 0 (0.0) | 0 (0.0) |  | 3 (0.0) | 2 (0.0) | 0 (0.0) | 1 (0.1) |
| **Level of education, *n (%)*** |  |  |  |  |  |  |  |  |  |
| Compulsory education or less | 3,269 (38.3) | 206 (17.9) | 109 (19.6) | 80 (20.7) |  | 3,047 (23.0) | 640 (13.3) | 289 (13.4) | 193 (13.9) |
| Upper secondary | 3,243 (38.0) | 533 (46.4) | 267 (48.0) | 173 (44.8) |  | 5,468 (41.3) | 2,000 (41.6) | 944 (43.7) | 614 (44.1) |
| College/ University/ Research | 1,915 (22.4) | 405 (35.3) | 178 (32.0) | 127 (32.9) |  | 4,597 (34.7) | 2,143 (44.6) | 918 (42.5) | 576 (41.4) |
| Unknown | 106 (1.2) | 5 (0.4) | 2 (0.4) | 6 (1.6) |  | 122 (0.9) | 22 (0.5) | 10 (0.5) | 9 (0.7) |
| **Disposable income SEK, *n (%)*** |  |  |  |  |  |  |  |  |  |
| 1 to 1,000 | 1,214 (14.2) | 110 (9.6) | 46 (8.3) | 47 (12.2) |  | 1,336 (10.1) | 333 (6.9) | 141 (6.5) | 108 (7.8) |
| 1,001 to 2,000 | 5,380 (63.1) | 488 (42.5) | 249 (44.8) | 183 (47.4) |  | 5,915 (44.7) | 1,472 (30.6) | 678 (31.4) | 479 (34.4) |
| 2,001 to 3,000 | 1,226 (14.4) | 351 (30.6) | 161 (29.0) | 108 (28.0) |  | 3,737 (28.2) | 1,853 (38.6) | 825 (38.2) | 511 (36.7) |
| > 3,000 | 697 (8.2) | 196 (17.1) | 96 (17.3) | 47 (12.2) |  | 2,137 (16.2) | 1,089 (22.7) | 488 (22.6) | 276 (19.8) |
| Unknown | 16 (0.2) | 4 (0.4) | 4 (0.7) | 1 (0.3) |  | 109 (0.8) | 58 (1.21) | 29 (1.3) | 18 (1.3) |
| **Tumour characteristics, *n (%)*** |  |  |  |  |  |  |  |  |  |
| **TNM stage** |  |  |  |  |  |  |  |  |  |
| Stage 1 | 4,635 (54.3) | 463 (40.3) | 234 (42.1) | 153 (39.6) |  | 8,878 (67.1) | 2,017 (42.0) | 904 (41.8) | 585 (42.0) |
| Stage 2 | 3,533 (41.4) | 614 (53.4) | 297 (53.4) | 206 (53.4) |  | 4,022 (30.4) | 2,526 (52.6) | 1,107 (51.2) | 738 (53.0) |
| Stage 3 | 365 (4.3) | 72 (6.3) | 25 (4.5) | 27 (7.0) |  | 334 (2.5) | 262 (5.5) | 150 (6.9) | 69 (5.0) |
| **Histological grade** |  |  |  |  |  |  |  |  |  |
| Grade 1 | 1,573 (18.4) | 58 (5.1) | 14 (2.5) | 3 (0.8) |  | 3,256 (24.6) | 284 (5.9) | 40 (1.9) | 6 (0.4) |
| Grade 2 | 3,773 (44.2) | 497 (43.3) | 143 (25.7) | 50 (13.0) |  | 6,736 (50.9) | 2,074 (43.2) | 528 (24.4) | 141 (10.1) |
| Grade 3 | 1,580 (18.5) | 463 (40.3) | 314 (56.5) | 274 (71.0) |  | 2,081 (15.7) | 1,803 (37.5) | 1,149 (53.2) | 979 (70.3) |
| Unknown | 1,607 (18.8) | 131 (11.4) | 85 (15.3) | 59 (15.3) |  | 1,161 (8.8) | 644 (13.4) | 444 (20.6) | 266 (19.1) |
| **Menopausal status** |  |  |  |  |  |  |  |  |  |
| Pre-menopausal | 334 (3.9) | 208 (18.1) | 88 (15.8) | 57 (14.8) |  | 2,487 (18.8) | 1,926 (40.1) | 820 (38.0) | 534 (38.4) |
| Post-menopausal | 7,825 (91.7) | 838 (72.9) | 418 (75.2) | 293 (75.9) |  | 9,682 (73.2) | 2,455 (51.1) | 1,123 (52.0) | 742 (53.3) |
| Unknown | 374 (4.38)_ | 103 (9.0) | 50 (9.0) | 36 (9.33) |  | 1,065 (8.1) | 424 (8.8) | 218 (10.1) | 116 (8.3) |

AI = aromatase inhibitors; COPD = chronic obstructive pulmonary disease; CKD = chronic kidney disease; CAD = coronary artery disease; ER = oestrogen receptor; GNRH = Gonadotropin-releasing hormone; HER2 = Human Epidermal Growth Factor Receptor 2; IQR = interquartile range; n/a = not applicable; PR = progesterone receptor; SD = standard deviation; TNM = UICC TNM Classification of Malignant Tumours.

* CVD history defined as having one or more of the following conditions at breast cancer diagnosis: coronary heart disease (CAD), heart failure, primary hypertension and stroke.

## Supplementary Table 11 Patient and tumour characteristics of patients with and without prior coronary artery disease (CAD) that received/did not receive trastuzumab.

|  | **HER2+ breast cancer patients  with a history of CAD at diagnosis** (N=173) | | |  | **HER2+ breast cancer patients  with no CAD history at diagnosis** (N=3,793) | | |
| --- | --- | --- | --- | --- | --- | --- | --- |
|  | **Trastuzumab**  (N=64, 37.0%) |  | **No trastuzumab**  (N=109, 63.0%) |  | **Trastuzumab**  (N=2,845, 75.0%) |  | **No trastuzumab**  (N=948, 25.0%) |
| **Type of cardiovascular disease, *n (%)*** | | | | |  |  |  |
| CAD | 64 (100.0) |  | 109 (100.0) |  | 0 (0.0) |  | 0 (0.0) |
| Heart failure | 7 (10.9) |  | 26 (23.9) |  | 15 (0.5) |  | 37 (3.9) |
| Hypertension | 37 (57.8) |  | 69 (63.3) |  | 265 (9.3) |  | 257 (27.1) |
| Stroke | 3 (4.7) |  | 14 (12.8) |  | 34 (1.2) |  | 54 (5.7) |
| VTE | 1 (1.6) |  | 7 (6.4) |  | 41 (1.4) |  | 30 (3.2) |
| **Demographics** |  |  |  |  |  |  |  |
| **Age at diagnosis (years)** |  |  |  |  |  |  |  |
| Mean (SD) | 67.8 (8.5) |  | 78.6 (9.7) |  | 55.7 (12.1) |  | 67.2 (15.4) |
| Median (IQR) | 68.5 (62.0, 73.5) |  | 79.0 (71.0, 87.0) |  | 56.0 (47.0, 65.0) |  | 68.5 (56.0, 80.0) |
| **Age group at diagnosis (years), *n (%)*** | |  |  |  |  |  |  |
| 18 to 39 | 0 (0.0) |  | 0 (0.0) |  | 298 (10.5) |  | 46 (4.9) |
| 40 to 59 | 10 (15.6) |  | 2 (1.8) |  | 1,399 (49.2) |  | 250 (26.4) |
| 60 to 79 | 50 (78.1) |  | 54 (49.5) |  | 1,111 (39.1) |  | 399 (42.1) |
| 80 plus | 4 (6.3) |  | 53 (48.6) |  | 37 (1.3) |  | 253 (26.7) |
| **Year of breast cancer diagnosis, *n (%)*** |  |  |  |  |  |  |  |
| 2010 to 2011 | 10 (15.6) |  | 40 (36.7) |  | 664 (23.3) |  | 383 (40.4) |
| 2012 to 2013 | 22 (34.4) |  | 28 (25.7) |  | 986 (34.7) |  | 220 (23.2) |
| 2014 to 2015 | 32 (50.0) |  | 41 (37.6) |  | 1,195 (42.0) |  | 345 (36.4) |
| **Region of Sweden, *n (%)*** |  |  |  |  |  |  |  |
| North | 6 (9.4) |  | 12 (11.0) |  | 245 (8.6) |  | 59 (6.2) |
| South | 12 (18.8) |  | 20 (18.3) |  | 527 (18.5) |  | 131 (13.8) |
| Southeast | 9 (14.1) |  | 9 (8.3) |  | 294 (10.3) |  | 107 (11.3) |
| Stockholm/Gotland | 16 (25.0) |  | 18 (16.5) |  | 710 (25.0) |  | 113 (11.9) |
| Uppsala/Örebro | 10 (15.6) |  | 32 (29.4) |  | 512 (18.0) |  | 332 (35.0) |
| West | 11 (17.2) |  | 18 (16.5) |  | 557 (19.6) |  | 206 (21.7) |
| **Civil status, *n (%)*** |  |  |  |  |  |  |  |
| Unmarried | 6 (9.4) |  | 10 (9.2) |  | 640 (22.5) |  | 149 (15.7) |
| Married | 31 (48.4) |  | 32 (29.4) |  | 1,539 (54.1) |  | 406 (42.8) |
| Divorced/separated | 15 (23.4) |  | 21 (19.3) |  | 493 (17.3) |  | 169 (17.8) |
| Widow | 12 (18.8) |  | 46 (42.2) |  | 173 (6.1) |  | 224 (23.6) |
| Unknown | 6 (9.4) |  | 10 (9.2) |  | 640 (22.5) |  | 149 (15.7) |
| **Level of education, *n (%)*** |  |  |  |  |  |  |  |
| Compulsory education or less | 21 (32.8) |  | 45 (41.3) |  | 425 (14.9) |  | 294 (31.0) |
| Upper secondary | 26 (40.6) |  | 44 (40.4) |  | 1,248 (43.9) |  | 380 (40.1) |
| College/ University/ Research | 17 (26.6) |  | 19 (17.4) |  | 1,154 (40.6) |  | 271 (28.6) |
| Unknown | 0 (0.0) |  | 1 (0.9) |  | 18 (0.6) |  | 3 (0.3) |
| **Disposable income SEK, *N (%)*** |  |  |  |  |  |  |  |
| 1 to 1,000 | 7 (10.9) |  | 18 (16.5) |  | 206 (7.2) |  | 118 (12.4) |
| 1,001 to 2,000 | 41 (64.1) |  | 75 (68.8) |  | 977 (34.3) |  | 535 (56.4) |
| 2,001 to 3,000 | 10 (15.6) |  | 13 (11.9) |  | 1,024 (36.0) |  | 193 (20.4) |
| > 3,000 | 5 (7.8) |  | 3 (2.8) |  | 606 (21.3) |  | 93 (9.8) |
| Unknown | 1 (1.6) |  | 0 (0.0) |  | 32 (1.1) |  | 9 (0.9) |
| **Tumour characteristics, *n (%)*** | | | | |  |  |  |
| **TNM stage** |  |  |  |  |  |  |  |
| Stage 1 | 18 (28.1) |  | 39 (35.8) |  | 1,174 (41.3) |  | 399 (42.1) |
| Stage 2 | 41 (64.1) |  | 61 (56.0) |  | 1,470 (51.7) |  | 486 (51.3) |
| Stage 3 | 5 (7.8) |  | 9 (8.3) |  | 201 (7.1) |  | 63 (6.6) |
| **Histological grade** |  |  |  |  |  |  |  |
| Grade 1 | 0 (0.0) |  | 3 (2.8) |  | 52 (1.8) |  | 66 (7.0) |
| Grade 2 | 13 (20.3) |  | 31 (28.4) |  | 693 (24.4) |  | 305 (32.2) |
| Grade 3 | 37 (57.8) |  | 58 (53.2) |  | 1,545 (54.3) |  | 476 (50.2) |
| Unknown | 14 (21.9) |  | 17 (15.6) |  | 555 (19.5) |  | 101 (10.7) |
| **ER status** |  |  |  |  |  |  |  |
| Negative | 39 (60.9) |  | 77 (70.6) |  | 1,854 (65.2) |  | 682 (71.9) |
| Positive | 25 (39.1) |  | 32 (29.4) |  | 991 (34.8) |  | 266 (28.1) |
| Unknown | 0 (0.0) |  | 0 (0.0) |  |  |  |  |
| **PR status** |  |  |  |  |  |  |  |
| Negative | 29 (45.3) |  | 51 (46.8) |  | 1,341 (47.1) |  | 500 (52.7) |
| Positive | 35 (54.7) |  | 58 (53.2) |  | 1,504 (52.9) |  | 448 (47.3) |
| Unknown | 0 (0.0) |  | 0 (0.0) |  |  |  |  |
| **Menopausal status** |  |  |  |  |  |  |  |
| Pre-menopausal | 3 (4.7) |  | 1 (0.9) |  | 923 (32.4) |  | 151 (15.9) |
| Post-menopausal | 54 (84.4) |  | 106 (97.2) |  | 1,644 (57.8) |  | 738 (77.8) |
| Uncertain (e.g., hysterectomy) | 3 (4.7) |  | 1 (0.9) |  | 154 (5.4) |  | 35 (3.7) |
| Unknown | 4 (6.3) |  | 1 (0.9) |  | 124 (4.4) |  | 24 (2.5) |
| **Health status at diagnosis** | | | | |  |  |  |
| Diabetes | 20 (31.3) |  | 25 (22.9) |  | 138 (4.9) |  | 100 (10.5) |
| CKD | 5 (7.8) |  | 12 (11.0) |  | 4 (0.1) |  | 8 (0.8) |
| COPD | 0 (0.0) |  | 0 (0.0) |  | 33 (1.2) |  | 34 (3.6) |
| **Breast cancer treatment in the year after diagnosis, *n (%)*** |  |  |  |  |  |  |  |
| **Surgery** |  |  |  |  |  |  |  |
| Primary operation | 50 (78.1) |  | 92 (84.4) |  | 2,301 (80.9) |  | 869 (91.7) |
| Pre-op oncological or conservative treatment | 13 (20.3) |  | 8 (7.3) |  | 540 (19.0) |  | 53 (5.6) |
| No surgery | 1 (1.6) |  | 9 (8.3) |  | 4 (0.1) |  | 25 (2.6) |
| Unknown | 0 (0.0) |  | 0 (0.0) |  | 0 (0.0) |  | 1 (0.1) |
| **Chemotherapy** |  |  |  |  |  |  |  |
| Anthracyclines | 42 (65.6) |  | 9 (8.3) |  | 2,519 (88.5) |  | 147 (15.5) |
| Docetaxel | 12 (18.8) |  | 1 (0.9) |  | 696 (24.5) |  | 31 (3.3) |
| Paclitaxel | 9 (14.1) |  | 2 (1.8) |  | 286 (10.1) |  | 9 (0.9) |
| Other chemotherapy | 10 (15.6) |  | 4 (3.7) |  | 170 (6.0) |  | 15 (1.6) |
| **Antibody therapy** | | | | | | | |
| Trastuzumab | 64 (100.0) |  | 0 (0.0) |  | 2,845 (100.0) |  | 0 (0.0) |
| Pertuzumab | 0 (0.0) |  | 0 (0.0) |  | 6 (0.2) |  | 1 (0.1) |
| **Endocrine therapy** | | | | | | | |
| Outpatient tamoxifen | 5 (7.8) |  | 16 (14.7) |  | 787 (27.7) |  | 217 (22.9) |
| Outpatient GNRH | 0 (0.0) |  | 0 (0.0) |  | 131 (4.6) |  | 11 (1.2) |
| Outpatient AI | 34 (53.1) |  | 67 (61.5) |  | 1,165 (40.9) |  | 444 (46.8) |
| **Radiotherapy** | | | | | | | |
| Not recorded | 23 (35.9) |  | 80 (73.4) |  | 614 (21.6) |  | 586 (61.8) |
| Right breast | 26 (40.6) |  | 14 (12.8) |  | 1,081 (38.0) |  | 186 (19.6) |
| Left breast | 15 (23.4) |  | 15 (13.8) |  | 1,150 (40.4) |  | 176 (18.6) |
|  |  |  |  |  |  |  |  |

AI = aromatase inhibitors; COPD = chronic obstructive pulmonary disease; CKD = chronic kidney disease; CAD = coronary artery disease; ER = oestrogen receptor; GNRH = Gonadotropin-releasing hormone; HER2 = Human Epidermal Growth Factor Receptor 2; IQR = interquartile range; n/a = not applicable; PR = progesterone receptor; SD = standard deviation; TNM = UICC TNM Classification of Malignant Tumours; VTE = venous thromboembolism.

## Supplementary Table 12 Patient and tumour characteristics of patients with and without prior heart failure (HF) that received/did not receive trastuzumab.

|  | **HER2+ breast cancer patients  with HF at diagnosis** (N=85) | | |  | **HER2+ breast cancer patients  with no history of HF at diagnosis** (N=) | | |
| --- | --- | --- | --- | --- | --- | --- | --- |
|  | **Trastuzumab**  (N=22, 25.9%) |  | **No trastuzumab**  (N=63, 74.1%) |  | **Trastuzumab**  (N=, %) |  | **No trastuzumab**  (N=, %) |
| **Type of cardiovascular disease, *n (%)*** | | | | |  |  |  |
| CAD | 7 (31.8) |  | 26 (41.3) |  | 57 (2.0) |  | 83 (8.4) |
| Heart failure | 22 (100.0) |  | 63 (100.0) |  | 0 (0.0) |  | 0 (0.0) |
| Hypertension | 11 (50.0) |  | 41 (65.1) |  | 291 (10.1) |  | 285 (28.7) |
| Stroke | 1 (4.5) |  | 14 (22.2) |  | 36 (1.2) |  | 54 (5.4) |
| VTE | 1 (4.5) |  | 8 (12.7) |  | 41 (1.4) |  | 29 (2.9) |
| **Demographics** |  |  |  |  |  |  |  |
| **Age at diagnosis (years)** |  |  |  |  |  |  |  |
| Mean (SD) | 66.0 (8.6) |  | 80.1 (8.6) |  | 55.9 (12.2) |  | 67.6 (15.3) |
| Median (IQR) | 68.0 (61.0, 72.0) |  | 81.0 (73.0, 86.0) |  | 57.0 (47.0, 65.0) |  | 69.0 (56.0, 80.0) |
| **Age group at diagnosis (years), *n (%)*** | |  |  |  |  |  |  |
| 18 to 39 | 0 (0.0) |  | 0 (0.0) |  | 298 (10.3) |  | 46 (4.6) |
| 40 to 59 | 5 (22.7) |  | 1 (1.6) |  | 1,404 (48.6) |  | 251 (25.3) |
| 60 to 79 | 16 (72.7) |  | 25 (39.7) |  | 1,145 (39.7) |  | 428 (43.1) |
| 80 plus | 1 (4.5) |  | 37 (58.7) |  | 40 (1.4) |  | 269 (27.1) |
| **Year of breast cancer diagnosis, *n (%)*** |  |  |  |  |  |  |  |
| 2010 to 2011 | 4 (18.2) |  | 13 (20.6) |  | 670 (23.2) |  | 410 (41.2) |
| 2012 to 2013 | 8 (36.4) |  | 22 (34.9) |  | 1,000 (34.6) |  | 226 (22.7) |
| 2014 to 2015 | 10 (45.5) |  | 28 (44.4) |  | 1,217 (42.2) |  | 358 (36.0) |
| **Region of Sweden, *n (%)*** |  |  |  |  |  |  |  |
| North | 2 (9.1) |  | 5 (7.9) |  | 249 (8.6) |  | 66 (6.6) |
| South | 4 (18.2) |  | 12 (19.0) |  | 535 (18.5) |  | 139 (14.0) |
| Southeast | 2 (9.1) |  | 5 (7.9) |  | 301 (10.4) |  | 111 (11.2) |
| Stockholm/Gotland | 7 (31.8) |  | 9 (14.3) |  | 719 (24.9) |  | 122 (12.3) |
| Uppsala/Örebro | 3 (13.6) |  | 20 (31.7) |  | 519 (18.0) |  | 344 (34.6) |
| West | 4 (18.2) |  | 12 (19.0) |  | 564 (19.5) |  | 212 (21.3) |
| **Civil status, *n (%)*** |  |  |  |  |  |  |  |
| Unmarried | 2 (9.1) |  | 6 (9.5) |  | 644 (22.3) |  | 153 (15.4) |
| Married | 9 (40.9) |  | 11 (17.5) |  | 1,561 (54.1) |  | 427 (43.0) |
| Divorced/separated | 10 (45.5) |  | 11 (17.5) |  | 498 (17.2) |  | 179 (18.0) |
| Widow | 1 (4.5) |  | 35 (55.6) |  | 184 (6.4) |  | 235 (23.6) |
| Unknown | 2 (9.1) |  | 6 (9.5) |  | 644 (22.3) |  | 153 (15.4) |
| **Level of education, *n (%)*** |  |  |  |  |  |  |  |
| Compulsory education or less | 8 (36.4) |  | 34 (54.0) |  | 438 (15.2) |  | 305 (30.7) |
| Upper secondary | 11 (50.0) |  | 21 (33.3) |  | 1,263 (43.7) |  | 403 (40.5) |
| College/ University/ Research | 3 (13.6) |  | 8 (12.7) |  | 1,168 (40.5) |  | 282 (28.4) |
| Unknown | 0 (0.0) |  | 0 (0.0) |  | 18 (0.6) |  | 4 (0.4) |
| **Disposable income SEK, *N (%)*** |  |  |  |  |  |  |  |
| 1 to 1,000 | 7 (31.8) |  | 8 (12.7) |  | 206 (7.1) |  | 128 (12.9) |
| 1,001 to 2,000 | 11 (50.0) |  | 51 (81.0) |  | 1,007 (34.9) |  | 559 (56.2) |
| 2,001 to 3,000 | 2 (9.1) |  | 3 (4.8) |  | 1,032 (35.7) |  | 203 (20.4) |
| > 3,000 | 2 (9.1) |  | 1 (1.6) |  | 609 (21.1) |  | 95 (9.6) |
| Unknown | 0 (0.0) |  | 0 (0.0) |  | 33 (1.1) |  | 9 (0.9) |
| **Tumour characteristics, *n (%)*** | | | | |  |  |  |
| **TNM stage** |  |  |  |  |  |  |  |
| Stage 1 | 7 (31.8) |  | 20 (31.7) |  | 1,185 (41.0) |  | 418 (42.1) |
| Stage 2 | 13 (59.1) |  | 40 (63.5) |  | 1,498 (51.9) |  | 507 (51.0) |
| Stage 3 | 2 (9.1) |  | 3 (4.8) |  | 204 (7.1) |  | 69 (6.9) |
| **Histological grade** |  |  |  |  |  |  |  |
| Grade 1 | 1 (4.5) |  | 0 (0.0) |  | 51 (1.8) |  | 69 (6.9) |
| Grade 2 | 3 (13.6) |  | 21 (33.3) |  | 703 (24.4) |  | 315 (31.7) |
| Grade 3 | 17 (77.3) |  | 30 (47.6) |  | 1,565 (54.2) |  | 504 (50.7) |
| Unknown | 1 (4.5) |  | 12 (19.0) |  | 568 (19.7) |  | 106 (10.7) |
| **ER status** |  |  |  |  |  |  |  |
| Negative | 11 (50.0) |  | 44 (69.8) |  | 1,882 (65.2) |  | 715 (71.9) |
| Positive | 11 (50.0) |  | 19 (30.2) |  | 1,005 (34.8) |  | 279 (28.1) |
| Unknown | 0 (0.0) |  | 0 (0.0) |  | 0 (0.0) |  | 0 (0.0) |
| **PR status** |  |  |  |  |  |  |  |
| Negative | 8 (36.4) |  | 32 (50.8) |  | 1,362 (47.2) |  | 519 (52.2) |
| Positive | 14 (63.6) |  | 31 (49.2) |  | 1,525 (52.8) |  | 475 (47.8) |
| Unknown | 0 (0.0) |  | 0 (0.0) |  | 0 (0.0) |  | 0 (0.0) |
| **Menopausal status** |  |  |  |  |  |  |  |
| Pre-menopausal | 1 (4.5) |  | 0 (0.0) |  | 925 (32.0) |  | 152 (15.3) |
| Post-menopausal | 18 (81.8) |  | 61 (96.8) |  | 1,680 (58.2) |  | 783 (78.8) |
| Uncertain (e.g., hysterectomy) | 2 (9.1) |  | 2 (3.2) |  | 155 (5.4) |  | 34 (3.4) |
| Unknown | 1 (4.5) |  | 0 (0.0) |  | 127 (4.4) |  | 25 (2.5) |
| **Health status at diagnosis** | | | | |  |  |  |
| Diabetes | 4 (18.2) |  | 15 (23.8) |  | 154 (5.3) |  | 110 (11.1) |
| CKD | 0 (0.0) |  | 2 (3.2) |  | 4 (0.1) |  | 6 (0.6) |
| COPD | 1 (4.5) |  | 11 (17.5) |  | 37 (1.3) |  | 35 (3.5) |
| **Breast cancer treatment in the year after diagnosis, *n (%)*** |  |  |  |  |  |  |  |
| **Surgery** |  |  |  |  |  |  |  |
| Primary operation | 21 (95.5) |  | 51 (81.0) |  | 2,330 (80.7) |  | 910 (91.5) |
| Pre-op oncological or conservative treatment | 1 (4.5) |  | 6 (9.5) |  | 552 (19.1) |  | 55 (5.5) |
| No surgery | 0 (0.0) |  | 6 (9.5) |  | 5 (0.2) |  | 28 (2.8) |
| Unknown | 0 (0.0) |  | 0 (0.0) |  | 0 (0.0) |  | 1 (0.1) |
| **Chemotherapy** |  |  |  |  |  |  |  |
| Anthracyclines | 12 (54.5) |  | 2 (3.2) |  | 2,549 (88.3) |  | 154 (15.5) |
| Docetaxel | 2 (9.1) |  | 0 (0.0) |  | 706 (24.5) |  | 32 (3.2) |
| Paclitaxel | 4 (18.2) |  | 0 (0.0) |  | 291 (10.1) |  | 11 (1.1) |
| Other chemotherapy | 5 (22.7) |  | 1 (1.6) |  | 175 (6.1) |  | 18 (1.8) |
| **Antibody therapy** | | | | | | | |
| Trastuzumab | 22 (100.0) |  | 0 (0.0) |  | 2,887 (100.0) |  | 0 (0.0) |
| Pertuzumab | 0 (0.0) |  | 0 (0.0) |  | 6 (0.2) |  | 1 (0.1) |
| **Endocrine therapy** | | | | | | | |
| Outpatient tamoxifen | 1 (4.5) |  | 6 (9.5) |  | 791 (27.4) |  | 227 (22.8) |
| Outpatient GNRH | 0 (0.0) |  | 0 (0.0) |  | 131 (4.5) |  | 11 (1.1) |
| Outpatient AI | 11 (50.0) |  | 40 (63.5) |  | 1,188 (41.1) |  | 471 (47.4) |
| **Radiotherapy** | | | | | | | |
| Not recorded | 4 (18.2) |  | 46 (73.0) |  | 633 (21.9) |  | 620 (62.4) |
| Right breast | 11 (50.0) |  | 8 (12.7) |  | 1,096 (38.0) |  | 192 (19.3) |
| Left breast | 7 (31.8) |  | 9 (14.3) |  | 1,158 (40.1) |  | 182 (18.3) |
|  |  |  |  |  |  |  |  |

AI = aromatase inhibitors; COPD = chronic obstructive pulmonary disease; CKD = chronic kidney disease; CAD = coronary artery disease; ER = oestrogen receptor; GNRH = Gonadotropin-releasing hormone; HER2 = Human Epidermal Growth Factor Receptor 2; IQR = interquartile range; n/a = not applicable; PR = progesterone receptor; SD = standard deviation; TNM = UICC TNM Classification of Malignant Tumours; VTE = venous thromboembolism.

## Supplementary Table 13 Patient and tumour characteristics of patients with and without prior hypertension that received/did not receive trastuzumab.

|  | **HER2+ breast cancer patients with hypertension at diagnosis** (N=628) | | |  | **HER2+ breast cancer patients without hypertension at diagnosis** (N=3,338) | | |
| --- | --- | --- | --- | --- | --- | --- | --- |
|  | **Trastuzumab**  (N=302, 48.1%) |  | **No trastuzumab**  (N=326, 51.9%) |  | **Trastuzumab**  (N=2,607, 78.1%) |  | **No trastuzumab**  (N=731, 21.9%) |
| **Type of cardiovascular disease, *n (%)*** | | | | |  |  |  |
| CAD | 37 (12.3) |  | 69 (21.2) |  | 27 (1.0) |  | 40 (5.5) |
| Heart failure | 11 (3.6) |  | 41 (12.6) |  | 11 (0.4) |  | 22 (3.0) |
| Hypertension | 302 (100.0) |  | 326 (100.0) |  | 0 (0.0) |  | 0 (0.0) |
| Stroke | 18 (6.0) |  | 51 (15.6) |  | 19 (0.7) |  | 17 (2.3) |
| VTE | 10 (3.3) |  | 22 (6.7) |  | 32 (1.2) |  | 15 (2.1) |
| **Demographics** |  |  |  |  |  |  |  |
| **Age at diagnosis (years)** |  |  |  |  |  |  |  |
| Mean (SD) | 65.6 (9.4) |  | 78.1 (9.1) |  | 54.8 (12.0) |  | 64.0 (15.5) |
| Median (IQR) | 66.0 (60.0, 72.0) |  | 79.0 (72.0, 85.0) |  | 55.0 (46.0, 64.0) |  | 64.0 (52.0, 77.0) |
| **Age group at diagnosis (years), *n (%)*** | |  |  |  |  |  |  |
| 18 to 39 | 4 (1.3) |  | 0 (0.0) |  | 294 (11.3) |  | 46 (6.3) |
| 40 to 59 | 62 (20.5) |  | 11 (3.4) |  | 1,347 (51.7) |  | 241 (33.0) |
| 60 to 79 | 217 (71.9) |  | 156 (47.9) |  | 944 (36.2) |  | 297 (40.6) |
| 80 plus | 19 (6.3) |  | 159 (48.8) |  | 22 (0.8) |  | 147 (20.1) |
| **Year of breast cancer diagnosis, *n (%)*** |  |  |  |  |  |  |  |
| 2010 to 2011 | 65 (21.5) |  | 92 (28.2) |  | 609 (23.4) |  | 331 (45.3) |
| 2012 to 2013 | 91 (30.1) |  | 93 (28.5) |  | 917 (35.2) |  | 155 (21.2) |
| 2014 to 2015 | 146 (48.3) |  | 141 (43.3) |  | 1,081 (41.5) |  | 245 (33.5) |
| **Region of Sweden, *n (%)*** |  |  |  |  |  |  |  |
| North | 27 (8.9) |  | 25 (7.7) |  | 224 (8.6) |  | 46 (6.3) |
| South | 46 (15.2) |  | 57 (17.5) |  | 493 (18.9) |  | 94 (12.9) |
| Southeast | 31 (10.3) |  | 37 (11.3) |  | 272 (10.4) |  | 79 (10.8) |
| Stockholm/Gotland | 82 (27.2) |  | 41 (12.6) |  | 644 (24.7) |  | 90 (12.3) |
| Uppsala/Örebro | 64 (21.2) |  | 106 (32.5) |  | 458 (17.6) |  | 258 (35.3) |
| West | 52 (17.2) |  | 60 (18.4) |  | 516 (19.8) |  | 164 (22.4) |
| **Civil status, *n (%)*** |  |  |  |  |  |  |  |
| Unmarried | 41 (13.6) |  | 24 (7.4) |  | 605 (23.2) |  | 135 (18.5) |
| Married | 165 (54.6) |  | 120 (36.8) |  | 1,405 (53.9) |  | 318 (43.5) |
| Divorced/separated | 48 (15.9) |  | 53 (16.3) |  | 460 (17.6) |  | 137 (18.7) |
| Widow | 48 (15.9) |  | 129 (39.6) |  | 137 (5.3) |  | 141 (19.3) |
| Unknown | 41 (13.6) |  | 24 (7.4) |  | 605 (23.2) |  | 135 (18.5) |
| **Level of education, *n (%)*** |  |  |  |  |  |  |  |
| Compulsory education or less | 79 (26.2) |  | 148 (45.4) |  | 367 (14.1) |  | 191 (26.1) |
| Upper secondary | 143 (47.4) |  | 116 (35.6) |  | 1,131 (43.4) |  | 308 (42.1) |
| College/ University/ Research | 77 (25.5) |  | 60 (18.4) |  | 1,094 (42.0) |  | 230 (31.5) |
| Unknown | 3 (1.0) |  | 2 (0.6) |  | 15 (0.6) |  | 2 (0.3) |
| **Disposable income SEK, *N (%)*** |  |  |  |  |  |  |  |
| 1 to 1,000 | 38 (12.6) |  | 51 (15.6) |  | 175 (6.7) |  | 85 (11.6) |
| 1,001 to 2,000 | 154 (51.0) |  | 235 (72.1) |  | 864 (33.1) |  | 375 (51.3) |
| 2,001 to 3,000 | 68 (22.5) |  | 24 (7.4) |  | 966 (37.1) |  | 182 (24.9) |
| > 3,000 | 40 (13.2) |  | 14 (4.3) |  | 571 (21.9) |  | 82 (11.2) |
| Unknown | 2 (0.7) |  | 2 (0.6) |  | 31 (1.2) |  | 7 (1.0) |
| **Tumour characteristics, *n (%)*** | | | | |  |  |  |
| **TNM stage** |  |  |  |  |  |  |  |
| Stage 1 | 113 (37.4) |  | 120 (36.8) |  | 1,079 (41.4) |  | 318 (43.5) |
| Stage 2 | 167 (55.3) |  | 182 (55.8) |  | 1,344 (51.6) |  | 365 (49.9) |
| Stage 3 | 22 (7.3) |  | 24 (7.4) |  | 184 (7.1) |  | 48 (6.6) |
| **Histological grade** |  |  |  |  |  |  |  |
| Grade 1 | 5 (1.7) |  | 9 (2.8) |  | 47 (1.8) |  | 60 (8.2) |
| Grade 2 | 77 (25.5) |  | 98 (30.1) |  | 629 (24.1) |  | 238 (32.6) |
| Grade 3 | 179 (59.3) |  | 184 (56.4) |  | 1,403 (53.8) |  | 350 (47.9) |
| Unknown | 41 (13.6) |  | 35 (10.7) |  | 528 (20.3) |  | 83 (11.4) |
| **ER status** |  |  |  |  |  |  |  |
| Negative | 188 (62.3) |  | 225 (69.0) |  | 1,705 (65.4) |  | 534 (73.1) |
| Positive | 114 (37.7) |  | 101 (31.0) |  | 902 (34.6) |  | 197 (26.9) |
| Unknown | 0 (0.0) |  | 0 (0.0) |  | 0 (0.0) |  | 0 (0.0) |
| **PR status** |  |  |  |  |  |  |  |
| Negative | 117 (38.7) |  | 160 (49.1) |  | 1,253 (48.1) |  | 391 (53.5) |
| Positive | 185 (61.3) |  | 166 (50.9) |  | 1,354 (51.9) |  | 340 (46.5) |
| Unknown | 0 (0.0) |  | 0 (0.0) |  | 0 (0.0) |  | 0 (0.0) |
| **Menopausal status** |  |  |  |  |  |  |  |
| Pre-menopausal | 17 (5.6) |  | 2 (0.6) |  | 909 (34.9) |  | 150 (20.5) |
| Post-menopausal | 259 (85.8) |  | 314 (96.3) |  | 1,439 (55.2) |  | 530 (72.5) |
| Uncertain (e.g., hysterectomy) | 16 (5.3) |  | 8 (2.5) |  | 141 (5.4) |  | 28 (3.8) |
| Unknown | 10 (3.3) |  | 2 (0.6) |  | 118 (4.5) |  | 23 (3.1) |
| **Health status at diagnosis** | | | | |  |  |  |
| Diabetes | 68 (22.5) |  | 73 (22.4) |  | 90 (3.5) |  | 52 (7.1) |
| CKD | 4 (1.3) |  | 7 (2.1) |  | 0 (0.0) |  | 1 (0.1) |
| COPD | 12 (4.0) |  | 27 (8.3) |  | 26 (1.0) |  | 19 (2.6) |
| **Breast cancer treatment in the year after diagnosis, *n (%)*** |  |  |  |  |  |  |  |
| **Surgery** |  |  |  |  |  |  |  |
| Primary operation | 262 (86.8) |  | 295 (90.5) |  | 2,089 (80.1) |  | 666 (91.1) |
| Pre-op oncological or conservative treatment | 40 (13.2) |  | 18 (5.5) |  | 513 (19.7) |  | 43 (5.9) |
| No surgery | 0 (0.0) |  | 13 (4.0) |  | 5 (0.2) |  | 21 (2.9) |
| Unknown | 0 (0.0) |  | 0 (0.0) |  | 0 (0.0) |  | 1 (0.1) |
| **Chemotherapy** |  |  |  |  |  |  |  |
| Anthracyclines | 234 (77.5) |  | 20 (6.1) |  | 2,327 (89.3) |  | 136 (18.6) |
| Docetaxel | 62 (20.5) |  | 2 (0.6) |  | 646 (24.8) |  | 30 (4.1) |
| Paclitaxel | 50 (16.6) |  | 1 (0.3) |  | 245 (9.4) |  | 10 (1.4) |
| Other chemotherapy | 31 (10.3) |  | 3 (0.9) |  | 149 (5.7) |  | 16 (2.2) |
| **Antibody therapy** | | | | | | | |
| Trastuzumab | 302 (100.0) |  | 0 (0.0) |  | 2,607 (100.0) |  | 0 (0.0) |
| Pertuzumab | 1 (0.3) |  | 0 (0.0) |  | 5 (0.2) |  | 1 (0.1) |
| **Endocrine therapy** | | | | | | | |
| Outpatient tamoxifen | 25 (8.3) |  | 33 (10.1) |  | 767 (29.4) |  | 200 (27.4) |
| Outpatient GNRH | 4 (1.3) |  | 0 (0.0) |  | 127 (4.9) |  | 11 (1.5) |
| Outpatient AI | 160 (53.0) |  | 198 (60.7) |  | 1,039 (39.9) |  | 313 (42.8) |
| **Radiotherapy** | | | | | | | |
| Not recorded | 81 (26.8) |  | 226 (69.3) |  | 556 (21.3) |  | 440 (60.2) |
| Right breast | 107 (35.4) |  | 52 (16.0) |  | 1,000 (38.4) |  | 148 (20.2) |
| Left breast | 114 (37.7) |  | 48 (14.7) |  | 1,051 (40.3) |  | 143 (19.6) |
|  |  |  |  |  |  |  |  |

AI = aromatase inhibitors; COPD = chronic obstructive pulmonary disease; CKD = chronic kidney disease; CAD = coronary artery disease; ER = oestrogen receptor; GNRH = Gonadotropin-releasing hormone; HER2 = Human Epidermal Growth Factor Receptor 2; IQR = interquartile range; n/a = not applicable; PR = progesterone receptor; SD = standard deviation; TNM = UICC TNM Classification of Malignant Tumours; VTE = venous thromboembolism.

## Supplementary Table 14 Patient and tumour characteristics of patients with and without prior stroke that received/did not receive trastuzumab.

|  | **HER2+ breast cancer patients**  **with a history of stroke at diagnosis** (N=105) | | |  | **HER2+ breast cancer patients  without stroke history diagnosis** (N=3,861) | | |
| --- | --- | --- | --- | --- | --- | --- | --- |
|  | **Trastuzumab**  (N=37, 35.2%) |  | **No trastuzumab**  (N=68, 64.8%) |  | **Trastuzumab**  (N=2,872, 74.4%) |  | **No trastuzumab**  (N=989, 25.6%) |
| **Type of cardiovascular disease, *n (%)*** | | | | |  |  |  |
| CAD | 3 (8.1) |  | 14 (20.6) |  | 61 (2.1) |  | 95 (9.6) |
| Heart failure | 1 (2.7) |  | 14 (20.6) |  | 21 (0.7) |  | 49 (5.0) |
| Hypertension | 18 (48.6) |  | 51 (75.0) |  | 284 (9.9) |  | 275 (27.8) |
| Stroke | 37 (100.0) |  | 68 (100.0) |  | 0 (0.0) |  | 0 (0.0) |
| VTE | 1 (2.7) |  | 4 (5.9) |  | 41 (1.4) |  | 33 (3.3) |
| **Demographics** |  |  |  |  |  |  |  |
| **Age at diagnosis (years)** |  |  |  |  |  |  |  |
| Mean (SD) | 65.2 (9.1) |  | 78.5 (9.5) |  | 55.8 (12.2) |  | 67.7 (15.4) |
| Median (IQR) | 65.0 (61.0, 70.0) |  | 80.0 (72.5, 85.0) |  | 57.0 (47.0, 65.0) |  | 69.0 (56.0, 80.0) |
| **Age group at diagnosis (years), *n (%)*** | |  |  |  |  |  |  |
| 18 to 39 | 0 (0.0) |  | 0 (0.0) |  | 298 (10.4) |  | 46 (4.7) |
| 40 to 59 | 7 (18.9) |  | 3 (4.4) |  | 1,402 (48.8) |  | 249 (25.2) |
| 60 to 79 | 29 (78.4) |  | 29 (42.6) |  | 1,132 (39.4) |  | 424 (42.9) |
| 80 plus | 1 (2.7) |  | 36 (52.9) |  | 40 (1.4) |  | 270 (27.3) |
| **Year of breast cancer diagnosis, *n (%)*** |  |  |  |  |  |  |  |
| 2010 to 2011 | 7 (18.9) |  | 14 (20.6) |  | 667 (23.2) |  | 409 (41.4) |
| 2012 to 2013 | 12 (32.4) |  | 22 (32.4) |  | 996 (34.7) |  | 226 (22.9) |
| 2014 to 2015 | 18 (48.6) |  | 32 (47.1) |  | 1,209 (42.1) |  | 354 (35.8) |
| **Region of Sweden, *n (%)*** |  |  |  |  |  |  |  |
| North | 7 (18.9) |  | 6 (8.8) |  | 244 (8.5) |  | 65 (6.6) |
| South | 11 (29.7) |  | 10 (14.7) |  | 528 (18.4) |  | 141 (14.3) |
| Southeast | 2 (5.4) |  | 5 (7.4) |  | 301 (10.5) |  | 111 (11.2) |
| Stockholm/Gotland | 6 (16.2) |  | 10 (14.7) |  | 720 (25.1) |  | 121 (12.2) |
| Uppsala/Örebro | 5 (13.5) |  | 19 (27.9) |  | 517 (18.0) |  | 345 (34.9) |
| West | 6 (16.2) |  | 18 (26.5) |  | 562 (19.6) |  | 206 (20.8) |
| **Civil status, *n (%)*** |  |  |  |  |  |  |  |
| Unmarried | 2 (5.4) |  | 5 (7.4) |  | 644 (22.4) |  | 154 (15.6) |
| Married | 19 (51.4) |  | 21 (30.9) |  | 1,551 (54.0) |  | 417 (42.2) |
| Divorced/separated | 10 (27.0) |  | 10 (14.7) |  | 498 (17.3) |  | 180 (18.2) |
| Widow | 6 (16.2) |  | 32 (47.1) |  | 179 (6.2) |  | 238 (24.1) |
| Unknown | 0 (0.0) |  | 0 (0.0) |  | 644 (22.4) |  | 154 (15.6) |
| **Level of education, *n (%)*** |  |  |  |  |  |  |  |
| Compulsory education or less | 12 (32.4) |  | 39 (57.4) |  | 434 (15.1) |  | 300 (30.3) |
| Upper secondary | 16 (43.2) |  | 21 (30.9) |  | 1,258 (43.8) |  | 403 (40.7) |
| College/ University/ Research | 8 (21.6) |  | 8 (11.8) |  | 1,163 (40.5) |  | 282 (28.5) |
| Unknown | 1 (2.7) |  | 0 (0.0) |  | 17 (0.6) |  | 4 (0.4) |
| **Disposable income SEK, *N (%)*** |  |  |  |  |  |  |  |
| 1 to 1,000 | 5 (13.5) |  | 13 (19.1) |  | 208 (7.2) |  | 123 (12.4) |
| 1,001 to 2,000 | 16 (43.2) |  | 50 (73.5) |  | 1,002 (34.9) |  | 560 (56.6) |
| 2,001 to 3,000 | 11 (29.7) |  | 4 (5.9) |  | 1,023 (35.6) |  | 202 (20.4) |
| > 3,000 | 5 (13.5) |  | 1 (1.5) |  | 606 (21.1) |  | 95 (9.6) |
| Unknown | 0 (0.0) |  | 0 (0.0) |  | 33 (1.1) |  | 9 (0.9) |
| **Tumour characteristics, *n (%)*** | | | | |  |  |  |
| **TNM stage** |  |  |  |  |  |  |  |
| Stage 1 | 12 (32.4) |  | 20 (29.4) |  | 1,180 (41.1) |  | 418 (42.3) |
| Stage 2 | 20 (54.1) |  | 45 (66.2) |  | 1,491 (51.9) |  | 502 (50.8) |
| Stage 3 | 5 (13.5) |  | 3 (4.4) |  | 201 (7.0) |  | 69 (7.0) |
| **Histological grade** |  |  |  |  |  |  |  |
| Grade 1 | 1 (2.7) |  | 2 (2.9) |  | 51 (1.8) |  | 67 (6.8) |
| Grade 2 | 7 (18.9) |  | 18 (26.5) |  | 699 (24.3) |  | 318 (32.2) |
| Grade 3 | 24 (64.9) |  | 36 (52.9) |  | 1,558 (54.2) |  | 498 (50.4) |
| Unknown | 5 (13.5) |  | 12 (17.6) |  | 564 (19.6) |  | 106 (10.7) |
| **ER status** |  |  |  |  |  |  |  |
| Negative | 24 (64.9) |  | 46 (67.6) |  | 1,869 (65.1) |  | 713 (72.1) |
| Positive | 13 (35.1) |  | 22 (32.4) |  | 1,003 (34.9) |  | 276 (27.9) |
| Unknown | 0 (0.0) |  | 0 (0.0) |  | 0 (0.0) |  | 0 (0.0) |
| **PR status** |  |  |  |  |  |  |  |
| Negative | 15 (40.5) |  | 36 (52.9) |  | 1,355 (47.2) |  | 515 (52.1) |
| Positive | 22 (59.5) |  | 32 (47.1) |  | 1,517 (52.8) |  | 474 (47.9) |
| Unknown | 0 (0.0) |  | 0 (0.0) |  | 0 (0.0) |  | 0 (0.0) |
| **Menopausal status** |  |  |  |  |  |  |  |
| Pre-menopausal | 2 (5.4) |  | 1 (1.5) |  | 924 (32.2) |  | 151 (15.3) |
| Post-menopausal | 33 (89.2) |  | 64 (94.1) |  | 1,665 (58.0) |  | 780 (78.9) |
| Uncertain (e.g., hysterectomy) | 2 (5.4) |  | 3 (4.4) |  | 155 (5.4) |  | 33 (3.3) |
| Unknown | 0 (0.0) |  | 0 (0.0) |  | 128 (4.5) |  | 25 (2.5) |
| **Health status at diagnosis** | | | | |  |  |  |
| Diabetes | 11 (29.7) |  | 15 (22.1) |  | 147 (5.1) |  | 110 (11.1) |
| CKD | 1 (2.7) |  | 2 (2.9) |  | 3 (0.1) |  | 6 (0.6) |
| COPD | 1 (2.7) |  | 7 (10.3) |  | 37 (1.3) |  | 39 (3.9) |
| **Breast cancer treatment in the year after diagnosis, *n (%)*** |  |  |  |  |  |  |  |
| **Surgery** |  |  |  |  |  |  |  |
| Primary operation | 32 (86.5) |  | 56 (82.4) |  | 2,319 (80.7) |  | 905 (91.5) |
| Pre-op oncological or conservative treatment | 5 (13.5) |  | 7 (10.3) |  | 548 (19.1) |  | 54 (5.5) |
| No surgery | 0 (0.0) |  | 5 (7.4) |  | 5 (0.2) |  | 29 (2.9) |
| Unknown | 0 (0.0) |  | 0 (0.0) |  | 0 (0.0) |  | 1 (0.1) |
| **Chemotherapy** |  |  |  |  |  |  |  |
| Anthracyclines | 26 (70.3) |  | 2 (2.9) |  | 2,535 (88.3) |  | 154 (15.6) |
| Docetaxel | 6 (16.2) |  | 0 (0.0) |  | 702 (24.4) |  | 32 (3.2) |
| Paclitaxel | 4 (10.8) |  | 1 (1.5) |  | 291 (10.1) |  | 10 (1.0) |
| Other chemotherapy | 4 (10.8) |  | 2 (2.9) |  | 176 (6.1) |  | 17 (1.7) |
| **Antibody therapy** | | | | | | | |
| Trastuzumab | 37 (100.0) |  | 0 (0.0) |  | 2,872 (100.0) |  | 0 (0.0) |
| Pertuzumab | 0 (0.0) |  | 0 (0.0) |  | 6 (0.2) |  | 1 (0.1) |
| **Endocrine therapy** | | | | | | | |
| Outpatient tamoxifen | 3 (8.1) |  | 4 (5.9) |  | 789 (27.5) |  | 229 (23.2) |
| Outpatient GNRH | 0 (0.0) |  | 0 (0.0) |  | 131 (4.6) |  | 11 (1.1) |
| Outpatient AI | 18 (48.6) |  | 43 (63.2) |  | 1,181 (41.1) |  | 468 (47.3) |
| **Radiotherapy** | | | | | | | |
| Not recorded | 7 (18.9) |  | 50 (73.5) |  | 630 (21.9) |  | 616 (62.3) |
| Right breast | 11 (29.7) |  | 9 (13.2) |  | 1,096 (38.2) |  | 191 (19.3) |
| Left breast | 19 (51.4) |  | 9 (13.2) |  | 1,146 (39.9) |  | 182 (18.4) |
|  |  |  |  |  |  |  |  |

AI = aromatase inhibitors; COPD = chronic obstructive pulmonary disease; CKD = chronic kidney disease; CAD = coronary artery disease; ER = oestrogen receptor; GNRH = Gonadotropin-releasing hormone; HER2 = Human Epidermal Growth Factor Receptor 2; IQR = interquartile range; n/a = not applicable; PR = progesterone receptor; SD = standard deviation; TNM = UICC TNM Classification of Malignant Tumours; VTE = venous thromboembolism.

## Supplementary Table 15 Patient and tumour characteristics of patients with and without prior venous thromboembolism (VTE) that received/did not receive trastuzumab.

|  | **HER2+ breast cancer patients  with VTE at diagnosis** (N=79) | | |  | **HER2+ breast cancer patients  without VTE at diagnosis** (N=3,887) | | |
| --- | --- | --- | --- | --- | --- | --- | --- |
|  | **Trastuzumab**  (N=42, 53.2%) |  | **No trastuzumab**  (N=37, 46.8%) |  | **Trastuzumab**  (N=2,867, 73.8%) |  | **No trastuzumab**  (N=1,020, 26.2%) |
| **Type of cardiovascular disease, *n (%)*** | | | | |  |  |  |
| CAD | 1 (2.4) |  | 7 (18.9) |  | 63 (2.2) |  | 102 (10.0) |
| Heart failure | 1 (2.4) |  | 8 (21.6) |  | 21 (0.7) |  | 55 (5.4) |
| Hypertension | 10 (23.8) |  | 22 (59.5) |  | 292 (10.2) |  | 304 (29.8) |
| Stroke | 1 (2.4) |  | 4 (10.8) |  | 36 (1.3) |  | 64 (6.3) |
| VTE | 42 (100.0) |  | 37 (100.0) |  | 0 (0.0) |  | 0 (0.0) |
| **Demographics** |  |  |  |  |  |  |  |
| **Age at diagnosis (years)** |  |  |  |  |  |  |  |
| Mean (SD) | 62.1 (12.7) |  | 81.2 (8.5) |  | 55.8 (12.2) |  | 67.9 (15.3) |
| Median (IQR) | 63.0 (54.0, 72.0) |  | 83.0 (76.0, 88.0) |  | 57.0 (47.0, 65.0) |  | 69.0 (57.0, 80.0) |
| **Age group at diagnosis (years), *n (%)*** | |  |  |  |  |  |  |
| 18 to 39 | 3 (7.1) |  | 0 (0.0) |  | 295 (10.3) |  | 46 (4.5) |
| 40 to 59 | 13 (31.0) |  | 1 (2.7) |  | 1,396 (48.7) |  | 251 (24.6) |
| 60 to 79 | 24 (57.1) |  | 14 (37.8) |  | 1,137 (39.7) |  | 439 (43.0) |
| 80 plus | 2 (4.8) |  | 22 (59.5) |  | 39 (1.4) |  | 284 (27.8) |
| **Year of breast cancer diagnosis, *n (%)*** |  |  |  |  |  |  |  |
| 2010 to 2011 | 5 (11.9) |  | 6 (16.2) |  | 669 (23.3) |  | 417 (40.9) |
| 2012 to 2013 | 14 (33.3) |  | 17 (45.9) |  | 994 (34.7) |  | 231 (22.6) |
| 2014 to 2015 | 23 (54.8) |  | 14 (37.8) |  | 1,204 (42.0) |  | 372 (36.5) |
| **Region of Sweden, *n (%)*** |  |  |  |  |  |  |  |
| North | 3 (7.1) |  | 2 (5.4) |  | 248 (8.7) |  | 69 (6.8) |
| South | 7 (16.7) |  | 6 (16.2) |  | 532 (18.6) |  | 145 (14.2) |
| Southeast | 10 (23.8) |  | 5 (13.5) |  | 293 (10.2) |  | 111 (10.9) |
| Stockholm/Gotland | 9 (21.4) |  | 6 (16.2) |  | 717 (25.0) |  | 125 (12.3) |
| Uppsala/Örebro | 7 (16.7) |  | 9 (24.3) |  | 515 (18.0) |  | 355 (34.8) |
| West | 6 (14.3) |  | 9 (24.3) |  | 562 (19.6) |  | 215 (21.1) |
| **Civil status, *n (%)*** |  |  |  |  |  |  |  |
| Unmarried | 4 (9.5) |  | 2 (5.4) |  | 642 (22.4) |  | 157 (15.4) |
| Married | 27 (64.3) |  | 14 (37.8) |  | 1,543 (53.8) |  | 424 (41.6) |
| Divorced/separated | 7 (16.7) |  | 4 (10.8) |  | 501 (17.5) |  | 186 (18.2) |
| Widow | 4 (9.5) |  | 17 (45.9) |  | 181 (6.3) |  | 253 (24.8) |
| Unknown | 0 (0.0) |  | 0 (0.0) |  | 0 (0.0) |  | 0 (0.0) |
| **Level of education, *n (%)*** |  |  |  |  |  |  |  |
| Compulsory education or less | 9 (21.4) |  | 21 (56.8) |  | 437 (15.2) |  | 318 (31.2) |
| Upper secondary | 23 (54.8) |  | 12 (32.4) |  | 1,251 (43.6) |  | 412 (40.4) |
| College/ University/ Research | 10 (23.8) |  | 4 (10.8) |  | 1,161 (40.5) |  | 286 (28.0) |
| Unknown | 0 (0.0) |  | 0 (0.0) |  | 18 (0.6) |  | 4 (0.4) |
| **Disposable income SEK, *n (%)*** |  |  |  |  |  |  |  |
| 1 to 1,000 | 5 (11.9) |  | 7 (18.9) |  | 208 (7.3) |  | 129 (12.6) |
| 1,001 to 2,000 | 19 (45.2) |  | 26 (70.3) |  | 999 (34.8) |  | 584 (57.3) |
| 2,001 to 3,000 | 14 (33.3) |  | 2 (5.4) |  | 1,020 (35.6) |  | 204 (20.0) |
| > 3,000 | 4 (9.5) |  | 2 (5.4) |  | 607 (21.2) |  | 94 (9.2) |
| Unknown | 0 (0.0) |  | 0 (0.0) |  | 33 (1.2) |  | 9 (0.9) |
| **Tumour characteristics, *n (%)*** | | | | |  |  |  |
| **TNM stage** |  |  |  |  |  |  |  |
| Stage 1 | 21 (50.0) |  | 9 (24.3) |  | 1,171 (40.8) |  | 429 (42.1) |
| Stage 2 | 21 (50.0) |  | 25 (67.6) |  | 1,490 (52.0) |  | 522 (51.2) |
| Stage 3 | 0 (0.0) |  | 3 (8.1) |  | 206 (7.2) |  | 69 (6.8) |
| **Histological grade** |  |  |  |  |  |  |  |
| Grade 1 | 3 (7.1) |  | 1 (2.7) |  | 49 (1.7) |  | 68 (6.7) |
| Grade 2 | 11 (26.2) |  | 8 (21.6) |  | 695 (24.2) |  | 328 (32.2) |
| Grade 3 | 25 (59.5) |  | 24 (64.9) |  | 1,557 (54.3) |  | 510 (50.0) |
| Unknown | 3 (7.1) |  | 4 (10.8) |  | 566 (19.7) |  | 114 (11.2) |
| **ER status** |  |  |  |  |  |  |  |
| Negative | 30 (71.4) |  | 25 (67.6) |  | 1,863 (65.0) |  | 734 (72.0) |
| Positive | 12 (28.6) |  | 12 (32.4) |  | 1,004 (35.0) |  | 286 (28.0) |
| Unknown | 0 (0.0) |  | 0 (0.0) |  | 0 (0.0) |  | 0 (0.0) |
| **PR status** |  |  |  |  |  |  |  |
| Negative | 22 (52.4) |  | 17 (45.9) |  | 1,348 (47.0) |  | 534 (52.4) |
| Positive | 20 (47.6) |  | 20 (54.1) |  | 1,519 (53.0) |  | 486 (47.6) |
| Unknown | 0 (0.0) |  | 0 (0.0) |  | 0 (0.0) |  | 0 (0.0) |
| **Menopausal status** |  |  |  |  |  |  |  |
| Pre-menopausal | 7 (16.7) |  | 0 (0.0) |  | 919 (32.1) |  | 152 (14.9) |
| Post-menopausal | 32 (76.2) |  | 37 (100.0) |  | 1,666 (58.1) |  | 807 (79.1) |
| Uncertain (e.g., hysterectomy) | 3 (7.1) |  | 0 (0.0) |  | 154 (5.4) |  | 36 (3.5) |
| Unknown | 0 (0.0) |  | 0 (0.0) |  | 128 (4.5) |  | 25 (2.5) |
| **Health status at diagnosis** | | | | |  |  |  |
| Diabetes | 3 (7.1) |  | 8 (21.6) |  | 155 (5.4) |  | 117 (11.5) |
| CKD | 0 (0.0) |  | 0 (0.0) |  | 4 (0.1) |  | 8 (0.8) |
| COPD | 0 (0.0) |  | 6 (16.2) |  | 38 (1.3) |  | 40 (3.9) |
| **Breast cancer treatment in the year after diagnosis, *n (%)*** |  |  |  |  |  |  |  |
| **Surgery** |  |  |  |  |  |  |  |
| Primary operation | 39 (92.9) |  | 33 (89.2) |  | 2,312 (80.6) |  | 928 (91.0) |
| Pre-op oncological or conservative treatment | 3 (7.1) |  | 2 (5.4) |  | 550 (19.2) |  | 59 (5.8) |
| No surgery | 0 (0.0) |  | 2 (5.4) |  | 5 (0.2) |  | 32 (3.1) |
| Unknown | 0 (0.0) |  | 0 (0.0) |  | 0 (0.0) |  | 1 (0.1) |
| **Chemotherapy** |  |  |  |  |  |  |  |
| Anthracyclines | 36 (85.7) |  | 2 (5.4) |  | 2,525 (88.1) |  | 154 (15.1) |
| Docetaxel | 9 (21.4) |  | 0 (0.0) |  | 699 (24.4) |  | 32 (3.1) |
| Paclitaxel | 9 (21.4) |  | 0 (0.0) |  | 286 (10.0) |  | 11 (1.1) |
| Other chemotherapy | 1 (2.4) |  | 0 (0.0) |  | 179 (6.2) |  | 19 (1.9) |
| **Antibody therapy** | | | | | | | |
| Trastuzumab | 42 (100.0) |  | 0 (0.0) |  | 2,867 (100.0) |  | 0 (0.0) |
| Pertuzumab | 0 (0.0) |  | 0 (0.0) |  | 6 (0.2) |  | 1 (0.1) |
| **Endocrine therapy** | | | | | | | |
| Outpatient tamoxifen | 6 (14.3) |  | 1 (2.7) |  | 786 (27.4) |  | 232 (22.7) |
| Outpatient GNRH | 3 (7.1) |  | 0 (0.0) |  | 128 (4.5) |  | 11 (1.1) |
| Outpatient AI | 27 (64.3) |  | 22 (59.5) |  | 1,172 (40.9) |  | 489 (47.9) |
| **Radiotherapy** | | | | | | | |
| Not recorded | 14 (33.3) |  | 27 (73.0) |  | 623 (21.7) |  | 639 (62.6) |
| Right breast | 15 (35.7) |  | 4 (10.8) |  | 1,092 (38.1) |  | 196 (19.2) |
| Left breast | 13 (31.0) |  | 6 (16.2) |  | 1,152 (40.2) |  | 185 (18.1) |
|  |  |  |  |  |  |  |  |

AI = aromatase inhibitors; COPD = chronic obstructive pulmonary disease; CKD = chronic kidney disease; CAD = coronary artery disease; ER = oestrogen receptor; GNRH = Gonadotropin-releasing hormone; HER2 = Human Epidermal Growth Factor Receptor 2; IQR = interquartile range; n/a = not applicable; PR = progesterone receptor; SD = standard deviation; TNM = UICC TNM Classification of Malignant Tumours; VTE = venous thromboembolism.

## Supplementary Table 16 Patient and tumour characteristics of patients with and without prior CVD, excluding hypertension, that received/did not receive trastuzumab. (Please note that patients with hypertension as well as other CVDs are still included.)

|  | **HER2+ breast cancer patients  with CVD exc. hypertension** (N=851) | | |  | **HER2+ breast cancer patients  with CVD exc. hypertension** (N=3,115) | | |
| --- | --- | --- | --- | --- | --- | --- | --- |
|  | **Trastuzumab**  (N=474, 55.7%) |  | **No trastuzumab**  (N=377, 44.3%) |  | **Trastuzumab**  (N=2,435, 78.2%) |  | **No trastuzumab**  (N=680, 21.8%) |
| **Type of cardiovascular disease, *n (%)*** | | | | |  |  |  |
| CAD | 64 (13.5) |  | 109 (28.9) |  | 0 (0.0) |  | 0 (0.0) |
| Heart failure | 22 (4.6) |  | 63 (16.7) |  | 0 (0.0) |  | 0 (0.0) |
| Hypertension | 125 (26.4) |  | 228 (60.5) |  | 177 (7.3) |  | 98 (14.4) |
| Stroke | 37 (7.8) |  | 68 (18.0) |  | 0 (0.0) |  | 0 (0.0) |
| **Demographics** |  |  |  |  |  |  |  |
| **Age at diagnosis (years)** |  |  |  |  |  |  |  |
| Mean (SD) | 62.1 (10.8) |  | 76.5 (11.0) |  | 54.7 (12.1) |  | 63.9 (15.5) |
| Median (IQR) | 63.0 (56.0, 70.0) |  | 78.0 (70.0, 85.0) |  | 55.0 (46.0, 64.0) |  | 64.5 (52.0, 77.0) |
| **Age group at diagnosis (years), *n (%)*** | |  |  |  |  |  |  |
| 18 to 39 | 18 (3.8) |  | 1 (0.3) |  | 280 (11.5) |  | 45 (6.6) |
| 40 to 59 | 150 (31.6) |  | 27 (7.2) |  | 1,259 (51.7) |  | 225 (33.1) |
| 60 to 79 | 295 (62.2) |  | 182 (48.3) |  | 866 (35.6) |  | 271 (39.9) |
| 80 plus | 11 (2.3) |  | 167 (44.3) |  | 30 (1.2) |  | 139 (20.4) |
| **Year of breast cancer diagnosis, *n (%)*** |  |  |  |  |  |  |  |
| 2010 to 2011 | 90 (19.0) |  | 121 (32.1) |  | 584 (24.0) |  | 302 (44.4) |
| 2012 to 2013 | 156 (32.9) |  | 108 (28.6) |  | 852 (35.0) |  | 140 (20.6) |
| 2014 to 2015 | 228 (48.1) |  | 148 (39.3) |  | 999 (41.0) |  | 238 (35.0) |
| **Region of Sweden, *n (%)*** |  |  |  |  |  |  |  |
| North | 39 (8.2) |  | 27 (7.2) |  | 212 (8.7) |  | 44 (6.5) |
| South | 95 (20.0) |  | 66 (17.5) |  | 444 (18.2) |  | 85 (12.5) |
| Southeast | 48 (10.1) |  | 37 (9.8) |  | 255 (10.5) |  | 79 (11.6) |
| Stockholm/Gotland | 111 (23.4) |  | 52 (13.8) |  | 615 (25.3) |  | 79 (11.6) |
| Uppsala/Örebro | 87 (18.4) |  | 113 (30.0) |  | 435 (17.9) |  | 251 (36.9) |
| West | 94 (19.8) |  | 82 (21.8) |  | 474 (19.5) |  | 142 (20.9) |
| **Civil status, *n (%)*** |  |  |  |  |  |  |  |
| Unmarried | 59 (12.4) |  | 34 (9.0) |  | 587 (24.1) |  | 125 (18.4) |
| Married | 272 (57.4) |  | 135 (35.8) |  | 1,298 (53.3) |  | 303 (44.6) |
| Divorced/separated | 91 (19.2) |  | 72 (19.1) |  | 417 (17.1) |  | 118 (17.4) |
| Widow | 52 (11.0) |  | 136 (36.1) |  | 133 (5.5) |  | 134 (19.7) |
| Unknown | 59 (12.4) |  | 34 (9.0) |  | 587 (24.1) |  | 125 (18.4) |
| **Level of education, *n (%)*** |  |  |  |  |  |  |  |
| Compulsory education or less | 105 (22.2) |  | 162 (43.0) |  | 341 (14.0) |  | 177 (26.0) |
| Upper secondary | 210 (44.3) |  | 136 (36.1) |  | 1,064 (43.7) |  | 288 (42.4) |
| College/ University/ Research | 157 (33.1) |  | 77 (20.4) |  | 1,014 (41.6) |  | 213 (31.3) |
| Unknown | 2 (0.4) |  | 2 (0.5) |  | 16 (0.7) |  | 2 (0.3) |
| **Disposable income SEK, *n (%)*** |  |  |  |  |  |  |  |
| 1 to 1,000 | 48 (10.1) |  | 56 (14.9) |  | 165 (6.8) |  | 80 (11.8) |
| 1,001 to 2,000 | 211 (44.5) |  | 257 (68.2) |  | 807 (33.1) |  | 353 (51.9) |
| 2,001 to 3,000 | 139 (29.3) |  | 39 (10.3) |  | 895 (36.8) |  | 167 (24.6) |
| > 3,000 | 74 (15.6) |  | 23 (6.1) |  | 537 (22.1) |  | 73 (10.7) |
| Unknown | 2 (0.4) |  | 2 (0.5) |  | 31 (1.3) |  | 7 (1.0) |
| **Tumour characteristics, *n (%)*** | | | | |  |  |  |
| **TNM stage** |  |  |  |  |  |  |  |
| Stage 1 | 196 (41.4) |  | 134 (35.5) |  | 996 (40.9) |  | 304 (44.7) |
| Stage 2 | 249 (52.5) |  | 217 (57.6) |  | 1,262 (51.8) |  | 330 (48.5) |
| Stage 3 | 29 (6.1) |  | 26 (6.9) |  | 177 (7.3) |  | 46 (6.8) |
| **Histological grade** |  |  |  |  |  |  |  |
| Grade 1 | 11 (2.3) |  | 11 (2.9) |  | 41 (1.7) |  | 58 (8.5) |
| Grade 2 | 113 (23.8) |  | 116 (30.8) |  | 593 (24.4) |  | 220 (32.4) |
| Grade 3 | 276 (58.2) |  | 205 (54.4) |  | 1,306 (53.6) |  | 329 (48.4) |
| Unknown | 74 (15.6) |  | 45 (11.9) |  | 495 (20.3) |  | 73 (10.7) |
| **ER status** |  |  |  |  |  |  |  |
| Negative | 301 (63.5) |  | 262 (69.5) |  | 1,592 (65.4) |  | 497 (73.1) |
| Positive | 173 (36.5) |  | 115 (30.5) |  | 843 (34.6) |  | 183 (26.9) |
| Unknown | 0 (0.0) |  | 0 (0.0) |  | 0 (0.0) |  | 0 (0.0) |
| **PR status** |  |  |  |  |  |  |  |
| Negative | 211 (44.5) |  | 181 (48.0) |  | 1,159 (47.6) |  | 370 (54.4) |
| Positive | 263 (55.5) |  | 196 (52.0) |  | 1,276 (52.4) |  | 310 (45.6) |
| Unknown | 0 (0.0) |  | 0 (0.0) |  | 0 (0.0) |  | 0 (0.0) |
| **Menopausal status** |  |  |  |  |  |  |  |
| Pre-menopausal | 77 (16.2) |  | 15 (4.0) |  | 849 (34.9) |  | 137 (20.1) |
| Post-menopausal | 358 (75.5) |  | 351 (93.1) |  | 1,340 (55.0) |  | 493 (72.5) |
| Unknown | 39 (8.3) |  | 11 (2.9) |  | 246 (10.1) |  | 50 (7.4) |
| **Health status at diagnosis** | | | | |  |  |  |
| Diabetes | 54 (11.4) |  | 69 (18.3) |  | 104 (4.3) |  | 56 (8.2) |
| CKD | 1 (0.2) |  | 5 (1.3) |  | 3 (0.1) |  | 3 (0.4) |
| COPD | 10 (2.1) |  | 34 (9.0) |  | 28 (1.1) |  | 12 (1.8) |
| **Breast cancer treatment in the year after diagnosis, *n (%)*** |  |  |  |  |  |  |  |
| **Surgery** |  |  |  |  |  |  |  |
| Primary operation | 403 (85.0) |  | 336 (89.1) |  | 1,948 (80.0) |  | 625 (91.9) |
| Pre-op oncological or conservative treatment | 70 (14.8) |  | 22 (5.8) |  | 483 (19.8) |  | 39 (5.7) |
| No surgery | 1 (0.2) |  | 19 (5.0) |  | 4 (0.2) |  | 15 (2.2) |
| Unknown | 0 (0.0) |  | 0 (0.0) |  | 0 (0.0) |  | 1 (0.1) |
| **Chemotherapy** |  |  |  |  |  |  |  |
| Anthracyclines | 389 (82.1) |  | 32 (8.5) |  | 2,172 (89.2) |  | 124 (18.2) |
| Docetaxel | 107 (22.6) |  | 5 (1.3) |  | 601 (24.7) |  | 27 (4.0) |
| Paclitaxel | 67 (14.1) |  | 3 (0.8) |  | 228 (9.4) |  | 8 (1.2) |
| Other chemotherapy | 42 (8.9) |  | 6 (1.6) |  | 138 (5.7) |  | 13 (1.9) |
| **Antibody therapy** | | | | | | | |
| Trastuzumab | 474 (100.0) |  | 0 (0.0) |  | 2,435 (100.0) |  | 0 (0.0) |
| Pertuzumab | 1 (0.2) |  | 0 (0.0) |  | 5 (0.2) |  | 1 (0.1) |
| **Endocrine therapy** | | | | | | | |
| Outpatient tamoxifen | 71 (15.0) |  | 54 (14.3) |  | 721 (29.6) |  | 179 (26.3) |
| Outpatient GNRH | 8 (1.7) |  | 0 (0.0) |  | 123 (5.1) |  | 11 (1.6) |
| Outpatient AI | 238 (50.2) |  | 221 (58.6) |  | 961 (39.5) |  | 290 (42.6) |
| **Radiotherapy** | | | | | | | |
| Not recorded | 122 (25.7) |  | 256 (67.9) |  | 515 (21.1) |  | 410 (60.3) |
| Right breast | 179 (37.8) |  | 58 (15.4) |  | 928 (38.1) |  | 142 (20.9) |
| Left breast | 173 (36.5) |  | 63 (16.7) |  | 992 (40.7) |  | 128 (18.8) |
|  |  |  |  |  |  |  |  |

AI = aromatase inhibitors; COPD = chronic obstructive pulmonary disease; CKD = chronic kidney disease; CAD = coronary artery disease; ER = oestrogen receptor; GNRH = Gonadotropin-releasing hormone; HER2 = Human Epidermal Growth Factor Receptor 2; IQR = interquartile range; n/a = not applicable; PR = progesterone receptor; SD = standard deviation; TNM = UICC TNM Classification of Malignant Tumours; VTE = venous thromboembolism.

## Supplementary Table 17 Patient and tumour characteristics of the 3,966 patients with HER2+ tumours, and without prior CVD that received/did not receive trastuzumab, stratified by neo-adjuvant and adjuvant setting.

|  | **HER2+ breast cancer patients with CVD* at diagnosis (N=1,100)** | | | |  | **HER2+ breast cancer patients without CVD* at diagnosis (N=2,866)** | | | |
| --- | --- | --- | --- | --- | --- | --- | --- | --- | --- |
|  | **No trastuzumab**  **(N=470, 42.7%)** | **Trastuzumab, any setting**  **(N=630, 57.3%)** | **Trastuzumab, neo-adjuvant setting †**  **(N=81)** | **Trastuzumab, adjuvant**  **setting**  **(N=623)** |  | **No trastuzumab**  **(N=587, 20.5%)** | **Trastuzumab, any setting**  **(N=2,279, 79.5%)** | **Trastuzumab, neo-adjuvant setting**  **(N=422)** | **Trastuzumab, adjuvant**  **setting**  **(N=2,253)** |
| **Type of cardiovascular disease, *n (%)*** |  |  |  |  |  |  |  |  |  |
| CAD | 109 (23.2) | 64 (10.2) | 10 (12.3) | 62 (10.0) |  | 0 (0.0) | 0 (0.0) | 0 (0.0) | 0 (0.0) |
| Heart failure | 63 (13.4) | 22 (3.5) | 1 (1.2) | 22 (3.5) |  | 0 (0.0) | 0 (0.0) | 0 (0.0) | 0 (0.0) |
| Hypertension | 326 (69.4) | 302 (47.9) | 31 (38.3) | 301 (48.3) |  | 0 (0.0) | 0 (0.0) | 0 (0.0) | 0 (0.0) |
| Stroke | 68 (14.5) | 37 (5.9) | 4 (4.9) | 37 (5.9) |  | 0 (0.0) | 0 (0.0) | 0 (0.0) | 0 (0.0) |
| **Demographics** |  |  |  |  |  |  |  |  |  |
| **Age at diagnosis (years)** |  |  |  |  |  |  |  |  |  |
| Mean (SD) | 76.5 (10.9) | 62.6 (10.4) | 59.3 (12.4) | 62.7 (10.3) |  | 61.9 (15.3) | 54.1 (12.0) | 50.0 (12.0) | 54.1 (12.0) |
| Median (IQR) | 78.0 (70.0, 84.0) | 64.0 (56.0, 70.0) | 62.0 (51.0, 69.0) | 64.0 (57.0, 70.0) |  | 62.0 (50.0, 72.0) | 54.0 (46.0, 63.0) | 49.0 (41.0, 58.0) | 54.0 (46.0, 63.0) |
| **Age group at diagnosis (years), *n (%)*** |  |  |  |  |  |  |  |  |  |
| 18 to 39 | 1 (0.2) | 19 (3.0) | 8 (9.9) | 18 (2.9) |  | 45 (7.7) | 279 (12.2) | 85 (20.1) | 276 (12.3) |
| 40 to 59 | 33 (7.0) | 192 (30.5) | 30 (37.0) | 188 (30.2) |  | 219 (37.3) | 1,217 (53.4) | 248 (58.8) | 1,201 (53.3) |
| 60 to 79 | 228 (48.5) | 398 (63.2) | 40 (49.4) | 396 (63.6) |  | 225 (38.3) | 763 (33.5) | 86 (20.4) | 758 (33.6) |
| 80 plus | 208 (44.3) | 21 (3.3) | 3 (3.7) | 21 (3.4) |  | 98 (16.7) | 20 (0.9) | 3 (0.7) | 18 (0.8) |
| **Year of breast cancer diagnosis, *n (%)*** |  |  |  |  |  |  |  |  |  |
| 2010 to 2011 | 152 (32.3) | 131 (20.8) | 9 (11.1) | 129 (20.7) |  | 271 (46.2) | 543 (23.8) | 70 (16.6) | 539 (23.9) |
| 2012 to 2013 | 129 (27.4) | 199 (31.6) | 16 (19.8) | 198 (31.8) |  | 119 (20.3) | 809 (35.5) | 121 (28.7) | 800 (35.5) |
| 2014 to 2015 | 189 (40.2) | 300 (47.6) | 56 (69.1) | 296 (47.5) |  | 197 (33.6) | 927 (40.7) | 231 (54.7) | 914 (40.6) |
| **Region of Sweden, *n (%)*** |  |  |  |  |  |  |  |  |  |
| North | 34 (7.2) | 48 (7.6) | 7 (8.6) | 45 (7.2) |  | 37 (6.3) | 203 (8.9) | 32 (7.6) | 201 (8.9) |
| South | 80 (17.0) | 118 (18.7) | 24 (29.6) | 116 (18.6) |  | 71 (12.1) | 421 (18.5) | 85 (20.1) | 418 (18.6) |
| Southeast | 46 (9.8) | 62 (9.8) | 6 (7.4) | 61 (9.8) |  | 70 (11.9) | 241 (10.6) | 32 (7.6) | 240 (10.7) |
| Stockholm/Gotland | 58 (12.3) | 155 (24.6) | 27 (33.3) | 155 (24.9) |  | 73 (12.4) | 571 (25.1) | 174 (41.2) | 568 (25.2) |
| Uppsala/Örebro | 154 (32.8) | 120 (19.0) | 9 (11.1) | 119 (19.1) |  | 210 (35.8) | 402 (17.6) | 42 (10.0) | 392 (17.4) |
| West | 98 (20.9) | 127 (20.2) | 8 (9.9) | 127 (20.4) |  | 126 (21.5) | 441 (19.4) | 57 (13.5) | 434 (19.3) |
| **Civil status, *n (%)*** |  |  |  |  |  |  |  |  |  |
| Unmarried | 39 (8.3) | 85 (13.5) | 14 (17.3) | 84 (13.5) |  | 120 (20.4) | 561 (24.6) | 134 (31.8) | 557 (24.7) |
| Married | 170 (36.2) | 357 (56.7) | 47 (58.0) | 351 (56.3) |  | 268 (45.7) | 1,213 (53.2) | 199 (47.2) | 1,204 (53.4) |
| Divorced/separated | 87 (18.5) | 116 (18.4) | 11 (13.6) | 116 (18.6) |  | 103 (17.5) | 392 (17.2) | 75 (17.8) | 380 (16.9) |
| Widow | 174 (37.0) | 72 (11.4) | 9 (11.1) | 72 (11.6) |  | 96 (16.4) | 113 (5.0) | 14 (3.3) | 112 (5.0) |
| Unknown | 39 (8.3) | 85 (13.5) | 14 (17.3) | 84 (13.5) |  | 0 (0.0) | 0 (0.0) | 0 (0.0) | 0 (0.0) |
| **Level of education, *n (%)*** |  |  |  |  |  |  |  |  |  |
| Compulsory education or less | 202 (43.0) | 134 (21.3) | 16 (19.8) | 133 (21.3) |  | 137 (23.3) | 312 (13.7) | 35 (8.3) | 307 (13.6) |
| Upper secondary | 166 (35.3) | 296 (47.0) | 37 (45.7) | 291 (46.7) |  | 258 (44.0) | 978 (42.9) | 183 (43.4) | 964 (42.8) |
| College/ University/ Research | 100 (21.3) | 195 (31.0) | 27 (33.3) | 194 (31.1) |  | 190 (32.4) | 976 (42.8) | 201 (47.6) | 969 (43.0) |
| Unknown | 2 (0.4) | 5 (0.8) | 1 (1.2) | 5 (0.8) |  | 2 (0.3) | 13 (0.6) | 3 (0.7) | 13 (0.6) |
| **Disposable income SEK, *n (%)*** |  |  |  |  |  |  |  |  |  |
| 1 to 1,000 | 71 (15.1) | 62 (9.8) | 7 (8.6) | 62 (10.0) |  | 65 (11.1) | 151 (6.6) | 33 (7.8) | 147 (6.5) |
| 1,001 to 2,000 | 322 (68.5) | 287 (45.6) | 34 (42.0) | 285 (45.7) |  | 288 (49.1) | 731 (32.1) | 110 (26.1) | 719 (31.9) |
| 2,001 to 3,000 | 49 (10.4) | 177 (28.1) | 30 (37.0) | 172 (27.6) |  | 157 (26.7) | 857 (37.6) | 159 (37.7) | 851 (37.8) |
| > 3,000 | 25 (5.3) | 100 (15.9) | 10 (12.3) | 100 (16.1) |  | 71 (12.1) | 511 (22.4) | 114 (27.0) | 507 (22.5) |
| Unknown | 3 (0.6) | 4 (0.6) | 0 (0.0) | 4 (0.6) |  | 6 (1.0) | 29 (1.3) | 6 (1.4) | 29 (1.3) |
| **Tumour characteristics, *n (%)*** |  |  |  |  |  |  |  |  |  |
| **TNM stage** |  |  |  |  |  |  |  |  |  |
| Stage 1 | 172 (36.6) | 254 (40.3) | 2 (2.5) | 254 (40.8) |  | 266 (45.3) | 938 (41.2) | 15 (3.6) | 937 (41.6) |
| Stage 2 | 263 (56.0) | 336 (53.3) | 52 (64.2) | 331 (53.1) |  | 284 (48.4) | 1,175 (51.6) | 286 (67.8) | 1,159 (51.4) |
| Stage 3 | 35 (7.4) | 40 (6.3) | 27 (33.3) | 38 (6.1) |  | 37 (6.3) | 166 (7.3) | 121 (28.7) | 157 (7.0) |
| **Histological grade** |  |  |  |  |  |  |  |  |  |
| Grade 1 | 13 (2.8) | 13 (2.1) | 1 (1.2) | 13 (2.1) |  | 56 (9.5) | 39 (1.7) | 1 (0.2) | 39 (1.7) |
| Grade 2 | 148 (31.5) | 153 (24.3) | 1 (1.2) | 153 (24.6) |  | 188 (32.0) | 553 (24.3) | 3 (0.7) | 553 (24.5) |
| Grade 3 | 257 (54.7) | 365 (57.9) | 0 (0.0) | 365 (58.6) |  | 277 (47.2) | 1,217 (53.4) | 8 (1.9) | 1,217 (54.0) |
| Unknown | 52 (11.1) | 99 (15.7) | 79 (97.5) | 92 (14.8) |  | 66 (11.2) | 470 (20.6) | 410 (97.2) | 444 (19.7) |
| **ER status** |  |  |  |  |  |  |  |  |  |
| Negative | 329 (70.0) | 395 (62.7) | 45 (55.6) | 391 (62.8) |  | 430 (73.3) | 1,498 (65.7) | 248 (58.8) | 1,484 (65.9) |
| Positive | 141 (30.0) | 235 (37.3) | 36 (44.4) | 232 (37.2) |  | 157 (26.7) | 781 (34.3) | 174 (41.2) | 769 (34.1) |
| Unknown | 0 (0.0) | 0 (0.0) | 0 (0.0) | 0 (0.0) |  | 0 (0.0) | 0 (0.0) | 0 (0.0) | 0 (0.0) |
| **PR status** |  |  |  |  |  |  |  |  |  |
| Negative | 233 (49.6) | 266 (42.2) | 34 (42.0) | 262 (42.1) |  | 318 (54.2) | 1,104 (48.4) | 184 (43.6) | 1,097 (48.7) |
| Positive | 237 (50.4) | 364 (57.8) | 47 (58.0) | 361 (57.9) |  | 269 (45.8) | 1,175 (51.6) | 238 (56.4) | 1,156 (51.3) |
| Unknown | 0 (0.0) | 0 (0.0) | 0 (0.0) | 0 (0.0) |  | 0 (0.0) | 0 (0.0) | 0 (0.0) | 0 (0.0) |
| **Menopausal status** |  |  |  |  |  |  |  |  |  |
| Pre-menopausal | 17 (3.6) | 84 (13.3) | 23 (28.4) | 81 (13.0) |  | 135 (23.0) | 842 (36.9) | 222 (52.6) | 835 (37.1) |
| Post-menopausal | 437 (93.0) | 489 (77.6) | 47 (58.0) | 486 (78.0) |  | 407 (69.3) | 1,209 (53.0) | 168 (39.8) | 1,193 (53.0) |
| Unknown | 16 (3.4) | 57 (9.1) | 11 (13.6) | 56 (9.0) |  | 45 (7.6) | 228 (10.0) | 32 (7.6) | 225 (9.9) |
| **Comorbidities at breast cancer diagnosis, *n (%)*** |  |  |  |  |  |  |  |  |  |
| Diabetes | 90 (19.1) | 87 (13.8) | 9 (11.1) | 85 (13.6) |  | 35 (6.0) | 71 (3.1) | 10 (2.4) | 70 (3.1) |
| CKD | 8 (1.7) | 4 (0.6) | 0 (0.0) | 4 (0.6) |  | 0 (0.0) | 0 (0.0) | 0 (0.0) | 0 (0.0) |
| COPD | 38 (8.1) | 17 (2.7) | 1 (1.2) | 17 (2.7) |  | 8 (1.4) | 21 (0.9) | 4 (0.9) | 21 (0.9) |
| **Breast cancer treatment in the year after diagnosis, *n (%)*** |  |  |  |  |  |  |  |  |  |
| **Surgery** |  |  |  |  |  |  |  |  |  |
| Primary operation | 422 (89.8) | 534 (84.8) | 1 (1.2) | 534 (85.7) |  | 539 (91.8) | 1,817 (79.7) | 3 (0.7) | 1,817 (80.6) |
| Pre-op oncological or conservative treatment | 27 (5.7) | 95 (15.1) | 79 (97.5) | 89 (14.3) |  | 34 (5.8) | 458 (20.1) | 416 (98.6) | 434 (19.3) |
| No surgery | 21 (4.5) | 1 (0.2) | 1 (1.2) | 0 (0.0) |  | 13 (2.2) | 4 (0.2) | 3 (0.7) | 2 (0.1) |
| Unknown | 427 (89.9) | 0 (0.0) | 0 (0.0) | 0 (0.0) |  | 1 (0.2) | 0 (0.0) | 0 (0.0) | 0 (0.0) |
| **Chemotherapy** |  |  |  |  |  |  |  |  |  |
| Anthracyclines | 37 (7.9) | 519 (82.4) | 70 (86.4) | 515 (82.7) |  | 119 (20.3) | 2,042 (89.6) | 384 (91.0) | 2,020 (89.7) |
| Docetaxel | 6 (1.3) | 145 (23.0) | 34 (42.0) | 142 (22.8) |  | 26 (4.4) | 563 (24.7) | 168 (39.8) | 556 (24.7) |
| Paclitaxel | 3 (0.6) | 88 (14.0) | 9 (11.1) | 88 (14.1) |  | 8 (1.4) | 207 (9.1) | 36 (8.5) | 203 (9.0) |
| Other chemotherapy | 7 (1.5) | 55 (8.7) | 9 (11.1) | 53 (8.5) |  | 12 (2.0) | 125 (5.5) | 24 (5.7) | 123 (5.5) |
| **Antibody therapy** |  |  |  |  |  |  |  |  |  |
| Trastuzumab | 0 (0.0) | 630 (100.0) | 81 (100.0) | 623 (100.0) |  | 0 (0.0) | 2,279 (100.0) | 422 (100.0) | 2,253 (100.0) |
| Pertuzumab | 0 (0.0) | 2 (0.3) | 1 (1.2) | 2 (0.3) |  | 1 (0.2) | 4 (0.2) | 3 (0.7) | 4 (0.2) |
| **Endocrine therapy** |  |  |  |  |  |  |  |  |  |
| Outpatient tamoxifen | 64 (13.6) | 85 (13.5) | 16 (19.8) | 82 (13.2) |  | 169 (28.8) | 707 (31.0) | 141 (33.4) | 702 (31.2) |
| Outpatient GNRH | 0 (0.0) | 9 (1.4) | 2 (2.5) | 9 (1.4) |  | 11 (1.9) | 122 (5.4) | 43 (10.2) | 122 (5.4) |
| Outpatient AI | 278 (59.1) | 313 (49.7) | 30 (37.0) | 313 (50.2) |  | 233 (39.7) | 886 (38.9) | 123 (29.1) | 877 (38.9) |
| **Radiotherapy** |  |  |  |  |  |  |  |  |  |
| Not recorded | 319 (67.9) | 155 (24.6) | 4 (4.9) | 153 (24.6) |  | 347 (59.1) | 482 (21.1) | 31 (7.3) | 472 (20.9) |
| Right breast | 71 (15.1) | 234 (37.1) | 40 (49.4) | 230 (36.9) |  | 129 (22.0) | 873 (38.3) | 196 (46.4) | 867 (38.5) |
| Left breast | 80 (17.0) | 241 (38.3) | 37 (45.7) | 240 (38.5) |  | 111 (18.9) | 924 (40.5) | 195 (46.2) | 914 (40.6) |
|  |  |  |  |  |  |  |  |  |  |

## Supplementary Figure 1 Selection of study population.

97,442 women aged ≥18 years, the National Quality Register for Breast Cancer, 2008-19

81,305 women with a **malignant** breast tumour

*No obvious primary tumour*

(women = 12,300)

*Tumours in situ*

(women = 4,972)

*Total exclusions*

(women = 17,223)

79,507 women with unilateral malignant breast cancer

*Synchronous bilateral breast cancer (left and right sided tumours diagnosed within 6 months)*

(women = 1,760)

*Second tumour diagnosed more than 6 months*

(women = 38)

*Total exclusions*

(women = 1,798)

*Immigrated/emigrated in year prior to diagnosis* (women = 143)

*Last migration status prior to diagnosis = emigration* (women= 5)

*Total exclusions*

(women= 148)

**32,590 women with unilateral primary non-metastatic malignant breast cancer diagnosed between 2010 to 2015, in women with no prior malignancies, and follow-up time available**

34,560 women with unilateral non-metastatic malignant breast cancer **diagnosed from 2010 to 2015**

*Breast cancer diagnosed in period
other than 2010-2015*

(women = 37,332)

72,369 women with unilateral **non-metastatic** malignant breast cancer

*Metastatic breast cancer*

(women = 2,374)

*Remote metastases could not be assessed*

(women = 4,764)

*Total exclusions*

(women = 7,138)

*Prior malignant tumour at another site*

(women = 1,345)

32,738 women with unilateral non-metastatic malignant breast cancer diagnosed in women from 2010 to 2015, with **no prior malignancies**

## Supplementary Figure 2 Association between cardiovascular diseases and anthracyclines use stratified by neo-adjuvant and adjuvant setting.

Min. adjusted = Prevalence ratios adjusted for age (5-year age groups).

Fully adjusted = Prevalence ratios adjusted for age (5-year age groups), region, socio-economic variables (education, civil status, and disposable income), comorbidities (chronic kidney disease, chronic obstructive pulmonary disease and diabetes), and tumour-related variables (stage at diagnosis, HER2 receptor status for anthracyclines, tumour grade and diagnostic subtype).

* Crude prevalences are shown.

CVD = cardiovascular disease, includes coronary heart disease (CAD), heart failure, primary hypertension and stroke. CAD = coronary artery disease; CI = confidence interval; Ref. = reference; VTE = venous thromboembolism.

## Supplementary Figure 3 Association between cardiovascular diseases and trastuzumab use stratified by neo-adjuvant and adjuvant setting.

Min. adjusted = Prevalence ratios adjusted for age (5-year age groups).

Fully adjusted = Prevalence ratios adjusted for age (5-year age groups), region, socio-economic variables (education, civil status, and disposable income), comorbidities (chronic kidney disease, chronic obstructive pulmonary disease and diabetes), and tumour-related variables (stage at diagnosis, HER2 receptor status for anthracyclines, tumour grade and diagnostic subtype).

* Crude prevalences are shown.

CVD = cardiovascular disease, includes coronary heart disease (CAD), heart failure, primary hypertension and stroke; CAD = coronary artery disease; CI = confidence interval; Ref. = reference; VTE = venous thromboembolism.
